# DEK oncoprotein participates in heterochromatin replication via SUMO-dependent nuclear bodies

**DOI:** 10.1242/jcs.261329

**Published:** 2023-12-15

**Authors:** Agnieszka Pierzynska-Mach, Christina Czada, Christopher Vogel, Eva Gwosch, Xenia Osswald, Denis Bartoschek, Alberto Diaspro, Ferdinand Kappes, Elisa Ferrando-May

**Affiliations:** ^1^ Nanoscopy & NIC@IIT, Istituto Italiano di Tecnologia, Genoa 16152, Italy; ^2^ Department of Biology, Bioimaging Center, University of Konstanz, Konstanz 78464, Germany; ^3^ DIFILAB, Department of Physics, University of Genoa, Genoa 16146, Italy; ^4^ Duke Kunshan University, Division of Natural and Applied Sciences, Kunshan 215316, People's Republic of China; ^5^ German Cancer Research Center, Heidelberg 69120, Germany

**Keywords:** Oncogene, Breast cancer, Replication stress, Histone modification, Superresolution microscopy, siRNA screen

## Abstract

The correct inheritance of chromatin structure is key for maintaining genome function and cell identity and preventing cellular transformation. DEK, a conserved non-histone chromatin protein, has recognized tumor-promoting properties, its overexpression being associated with poor prognosis in various cancer types. At the cellular level, DEK displays pleiotropic functions, influencing differentiation, apoptosis and stemness, but a characteristic oncogenic mechanism has remained elusive. Here, we report the identification of DEK bodies, focal assemblies of DEK that regularly occur at specific, yet unidentified, sites of heterochromatin replication exclusively in late S-phase. In these bodies, DEK localizes in direct proximity to active replisomes in agreement with a function in the early maturation of heterochromatin. A high-throughput siRNA screen, supported by mutational and biochemical analyses, identifies SUMO as one regulator of DEK body formation, linking DEK to the complex SUMO protein network that controls chromatin states and cell fate. This work combines and refines our previous data on DEK as a factor essential for heterochromatin integrity and facilitating replication under stress, and delineates an avenue of further study for unraveling the contribution of DEK to cancer development.

## Teaser

**Highlighted Article:** The human oncoprotein DEK forms nuclear bodies that are regulated by the SUMO pathway and localize to active sites of constitutive heterochromatin replication.

## INTRODUCTION

Replication of genomes is a fundamental process requiring meticulously orchestrated, yet adaptable mechanisms in space and time. The replication machinery encounters a range of different chromatin structures, some of which are prone to delaying, impairing or even stalling replication fork progression – a state referred to as DNA replication stress (Maya-Mendoza et al., 2018) and which is often encountered in cancer cells (Boyer et al., 2016; Saxena and Zou, 2022). Overcoming such obstacles requires the coordinated action of a number of protective chromatin factors, often governed by targeted post-translational modifications, with poly(ADP)ribosylation and SUMOylation playing prominent roles (Abbas, 2021; Stewart-Morgan et al., 2020). Active DNA replication forks, as visualized by incorporation of DNA precursors (BrdU, EdU or other thymidine analogs) or via marking DNA replication factors (e.g. proliferating cell nuclear antigen, PCNA) (Chagin et al., 2016; Jackson and Pombo, 1998) appear as a characteristic pattern of nuclear foci that changes over time and typically reflects the chromatin structure being replicated (Aladjem, 2007; Casas-Delucchi et al., 2012; Heinz et al., 2018; O'Keefe et al., 1992; Schwaiger et al., 2009). In general, euchromatin undergoes DNA replication during early S-phase, facultative heterochromatin during middle S-phase, and constitutive heterochromatin during late S-phase (Chadwick and Willard, 2004; Fragkos et al., 2015; Ryba et al., 2010; Su et al., 2020). However, some ‘hard-to-replicate’ DNA structures, for example, the inactivated X chromosome, and telomeres, centromeres and certain DNA secondary structures, such as G-quadruplexes, four-way junction DNA and others, pose a particular challenge to the DNA replication machinery, and, in turn, require fine-tuned action of additional accessory factors (Mirkin and Mirkin, 2007).

We and others have identified the DEK oncogene, a unique and multifunctional non-histone chromosomal protein, as a supportive factor for DNA replication, particularly in scenarios where the replication machinery is under stress (Deutzmann et al., 2015; Ganz et al., 2019). Although ‘isolation of proteins on nascent DNA’ (iPOND) studies support the idea that there is no direct association between DEK and the replisome, DEK is enriched in maturing chromatin (Alabert et al., 2014; Aranda et al., 2014; Garcia et al., 2017; Lossaint et al., 2013; Ribeyre et al., 2016; Sirbu et al., 2013), in line with its post-translational modification-dependent function as a histone chaperone and its role in the maintenance of chromatin structure (Alexiadis et al., 2000; Böhm et al., 2005; Cavellán et al., 2006; Gamble and Fisher, 2007; Guo et al., 2021a; Hollenbach et al., 2002; Kappes et al., 2001, 2004a,b, 2011a; Privette Vinnedge et al., 2013; Saha et al., 2013; Sawatsubashi et al., 2010; Tabbert et al., 2006; Waidmann et al., 2014; Waldmann et al., 2002, 2003). Under moderate to severe replication stress, DEK is highly enriched at maturing chromatin, suggesting a protective role at the replication fork. Our previous research has demonstrated that downregulation of DEK in cells under moderate short-term replication stress via aphidicolin and camptothecin treatment results in the aggravation of replication stress phenotypes. Replication fork progression, proliferative capability and clonogenic survival were impaired, and cells accumulated replication-born DNA damage and transmitted it to daughter cells (Deutzmann et al., 2015). Such fork-protective properties of DEK are modulated by PARP1 and PARP2 activity depending on the type and extent of replication stress (Ganz et al., 2019). At the same time, DEK appears to also safeguard DNA replication under steady-state, unchallenged conditions, as DNA fiber assays showed a mild decrease in fork speed when DEK expression is downregulated in untreated cells (Deutzmann et al., 2015). Collectively, these findings suggest that DEK is not a direct component of the replisome but binds in the vicinity of the fork to facilitate fork progression by supporting chromatin maturation. Evidence for a role of DEK in DNA replication via its properties as a chromatin architectural protein comes also from *in vitro* studies – experiments with SV40 mini-chromosomes have shown that DEK introduces positive supercoils to chromatin and reduces the replication efficiency of chromatin in a reconstituted system (Alexiadis et al., 2000; Waldmann et al., 2002). Furthermore, DEK preferentially associates with four-way junctions over duplex DNA and with supercoiled over linear DNA, both structures that can induce replication stress per se or that appear as a consequence of replication stress (Böhm et al., 2005; Waldmann et al., 2003).

Under steady-state conditions DEK associates with chromatin throughout the cell cycle (Kappes et al., 2001; Matrka et al., 2015), typically observed as pan-nuclear punctate pattern in immunofluorescence images and as further supported by the requirement for medium to high salt concentrations for its extraction from nuclei preparations (Kappes et al., 2001, 2004a, 2008; Özçelik et al., 2022; Saha et al., 2013). This is in line with its pleiotropic involvement in multiple nuclear processes, ranging from transcriptional regulation, the DNA damage response and repair, apoptosis, cell stemness and chromatin maintenance (Privette Vinnedge et al., 2013; Waldmann et al., 2004). More detailed analyses have revealed that DEK can associate both with euchromatin and heterochromatin (Capitano et al., 2022; Gamble and Fisher, 2007; Guo et al., 2021a; Hollenbach et al., 2002; Hu et al., 2005; Ivanauskiene et al., 2014; Kappes et al., 2001, 2004a,b, 2008, 2011b; Karam et al., 2014; Privette Vinnedge et al., 2013; Saha et al., 2013; Sandén and Gullberg, 2015; Sandén et al., 2014; Sawatsubashi et al., 2010; Waidmann et al., 2014; Zhou et al., 2022). However, qualitative and quantitative differences in the specific chromatin distribution of DEK among cell types and conditions are evident. Although DEK was identified as a suppressor of variegation [Su(var)], thus as a factor important for maintenance of heterochromatin (Capitano et al., 2019; Kappes et al., 2011a; Saha et al., 2013), other studies show preferential association with euchromatin (Hu et al., 2007; Ivanauskiene et al., 2014; Sandén et al., 2014; Yang et al., 2022), pinpointing that cell-type-specific post-translational modifications on DEK are critical for its specific chromatin association. Indeed, DEK is targeted by a plethora of post-translational modifications, with biological effects for acetylation, phosphorylation and poly(ADP)ribosylation having been reported (Cleary et al., 2005; Fahrer et al., 2010; Kappes et al., 2004a,b, 2008; Mor-Vaknin et al., 2011; Tabbert et al., 2006). Moreover, DEK contains an RNA-binding motif and is found associated with cellular RNA (Baltz et al., 2012; Beckmann et al., 2015; Castello et al., 2012; Guo et al., 2021a,b). These data delineate DEK as a multifaceted protein subject to complex regulation and impacting multiple nuclear processes.

To deepen our insight into the functional mechanisms of DEK, in particular related to DNA replication, we investigated the dynamic localization of DEK in the nucleus throughout the cell cycle by live and high-resolution microscopy including optical nanoscopy (Diaspro and Bianchini, 2020). We discovered that, in late S-phase, DEK consistently forms discrete foci, which we termed DEK bodies, that locate in close proximity of active heterochromatic replication sites. We then conducted an imaging-based siRNA screen to identify cellular regulators involved in the formation of DEK bodies. Besides methyltransferases, acetylases and proteins involved in checkpoint regulation, we surprisingly identified SUMO and ubiquitin enzymes among the candidates. Driven by this finding, we show that DEK is targeted by SUMOylation *in vitro* and in cells, and that SUMO modifications are required, at least in part, for the establishment of DEK bodies. Our data refine our previous knowledge about the involvement of DEK in DNA replication and put forward DEK as a cofactor in the duplication of specific heterochromatic regions, including pericentric chromatin. These findings provide further insights into the post-translational-dependent mechanisms by which DEK contributes to heterochromatin formation and maintenance with consequences for a variety of cellular processes.

## RESULTS

### A subpopulation of DEK molecules assembles into nuclear bodies at sites of late DNA replication

**Fig. 1.**
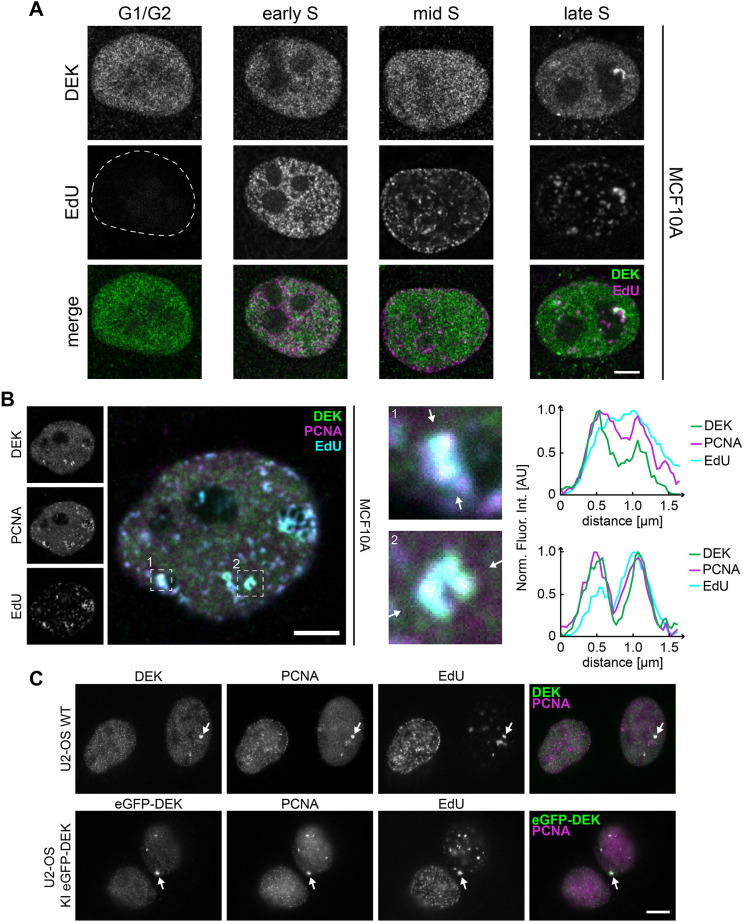
**DEK forms distinct foci, called DEK bodies, which colocalize with nascent DNA markers.** (A) Representative confocal images of MCF10A cells. Nascent DNA was pulse-labeled with EdU for 25 min followed by detection via click chemistry and immunofluorescence staining using DEK-specific antibodies (Santa Cruz Biotechnology). The mean±s.d. number of DEK foci per cell in S-phase was 6.7±2.2 (*n*=124 cells; 11 biological replicates). Magenta, EdU; green; DEK. Dashed line indicates the edge of the nucleus. (B) Immunofluorescence and colocalization analysis of the DEK, EdU and PCNA distribution pattern in late S-phase MCF10A cells as described in A (maximum projection). Dashed boxes indicate two selected ROIs. The intensity profiles are measured along the lines shown in the enlarged ROIs. The fluorescence intensity was normalized to 1. Arrows point to sites of DEK, EdU and PCNA colocalization. AU, arbitrary units. (C) Representative confocal fluorescence images of immunolabelled U2-OS wild-type cells (WT; upper row) and U2-OS KI eGFP–DEK cells (bottom row). Endogenous DEK (green, K-877 Ab) and PCNA (magenta) were visualized by specific antibodies. In each image one prominent DEK body is marked by a white arrow. Images in B and C representative of 124 and 14 experimental repeats, respectively. Scale bars: 5 µm.

To obtain precise insights into the distribution of DEK on chromatin in the context of DNA replication, we first investigated its subnuclear localization throughout the cell cycle along with EdU or PCNA as replication fork markers. Confocal imaging of endogenous DEK in EdU-labeled MCF10A cells (Fig. 1A) revealed an archetypical, mostly uniform and punctuate nuclear pattern of DEK in G1/G2 and early to mid S-phase, in full agreement with previous observations as outlined above. In late S-phase, however, previously undescribed, distinct focal assemblies of DEK became visible, which located in spatial proximity or within DNA replication foci as assessed by EdU pulse labeling and by PCNA immunofluorescence (Fig. 1A,B). We made the same observation in wild-type U2-OS cells (Fig. 1C, upper row) and primary BJ-5ta cells (Fig. S1A–C), where S-phase was assessed by PCNA staining. Interestingly, in contrast to immortalized MCF10A cells, fewer DEK foci were formed in transformed U2-OS osteosarcoma cells and no or very few foci occurred under identical conditions in MCF7 or MDA-MB-231 metastatic breast cancer cells (Fig. S1D,E). This might suggest general cell-type-dependent differences in DEK foci formation. Focal accumulations of DEK in late S-phase were also observed with a previously established U2-OS cell line carrying a TALEN-mediated knock-in of eGFP–DEK (denoted U2-OS KI eGFP-DEK cells; Ganz et al., 2019) (Fig. 1C, lower panel; Fig. S2A,B) and when DEK was expressed transiently in U2-OS cells (Fig. S2C). Such accumulations were not detected in cells only expressing eGFP (Fig. S2D). Thus, formation of late replication-associated foci is a specific feature of DEK, and is possibly regulated by cellular state, for which the U2-OS KI eGFP-DEK cell line is a valid reporter. We henceforth term these foci ‘DEK bodies’.

**Fig. 2.**
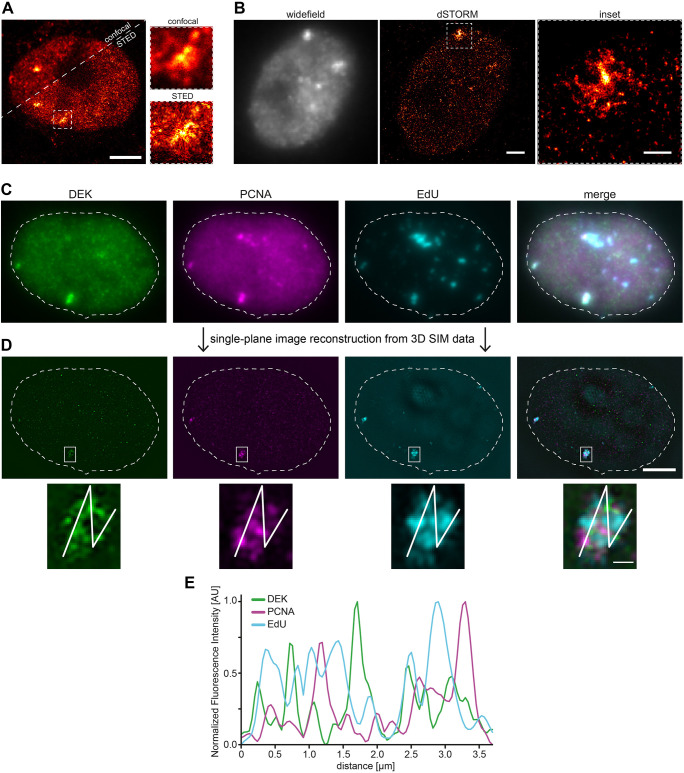
**DEK bodies localize in the vicinity of the replication fork.** (A) Representative confocal and STED super-resolution images of an MCF10A cell nucleus in late S-phase labeled with anti-DEK antibodies (Santa Cruz Biotechnology). The STED image reveals an intricate granular internal structure of DEK bodies (insets). Scale bar: 5 µm. (B) dSTORM images of an MCF10A cell nucleus in late S-phase labeled with anti-DEK antibodies (Santa Cruz Biotechnology). Left, widefield conventional image of DEK immunolabelling. Middle, dSTORM image reconstructed from single-molecule localization microscopy acquisition. Scale bar: 2 µm. Right, magnified image of the ROI shown in the middle containing a DEK body. For A and B, representative results are from 11 and four cells, respectively, and two biological replicates. Scale bar: 0.5 µm. (C) Pseudo-widefield representation of 20 slices (0.125 µm step size in z) of a representative U2-OS nucleus in late S-phase imaged in 3D-SIM mode. U2-OS cells were pulse-labelled with EdU for 10 min. DEK (green, K-877 antibody) and PCNA (magenta) were visualized via indirect immunofluorescence microscopy and EdU (cyan) using click chemistry. Pseudo-widefield images were generated with Fiji software. (D) Single Z-slice from a middle section of the super-resolved image stack after reconstruction and registration. Scale bar: 5 µm. The magnified inset shows one DEK-positive large replication focus. Scale bar: 0.5 µm. Dashed lines indicate the edge of the nucleus. (E) Fluorescence intensity profiles of DEK, PCNA and EdU. Interpolated intensity profiles were calculated along the line shown in D and normalized to the minimum and maximum values. AU, arbitrary units. For C and D, representative results are from 11 cells from two biological replicates.

To get deeper structural insight into DEK bodies and their spatial relation to active replication sites, we turned to multiple super-resolution imaging techniques available to us. We visualized firstly the granular internal structure of endogenous DEK bodies in MCF10A cells by stimulated emission depletion (STED) and single-molecule localization (SML) microscopy (Fig. 2A,B). Secondly, we investigated the organization of nascent DNA and replication forks within DEK bodies of wild-type U2-OS cells by labeling with EdU and PCNA-specific antibodies followed by 3D structured illumination microscopy (3D-SIM). The data show that DEK, PCNA and EdU signals do not colocalize but are juxtaposed within DEK bodies (Fig. 2C,D). Intensity line plots (Fig. 2E) highlight the close spatial proximity of the signals, which was also observed in primary BJ-5ta foreskin fibroblasts (Fig. S1). This result is in line with previous data showing that DEK is not part of the replisome but binds to nascent chromatin in the vicinity of the replication fork (Ganz et al., 2019).

### DEK bodies assemble at replicating heterochromatic regions

**Fig. 3.**
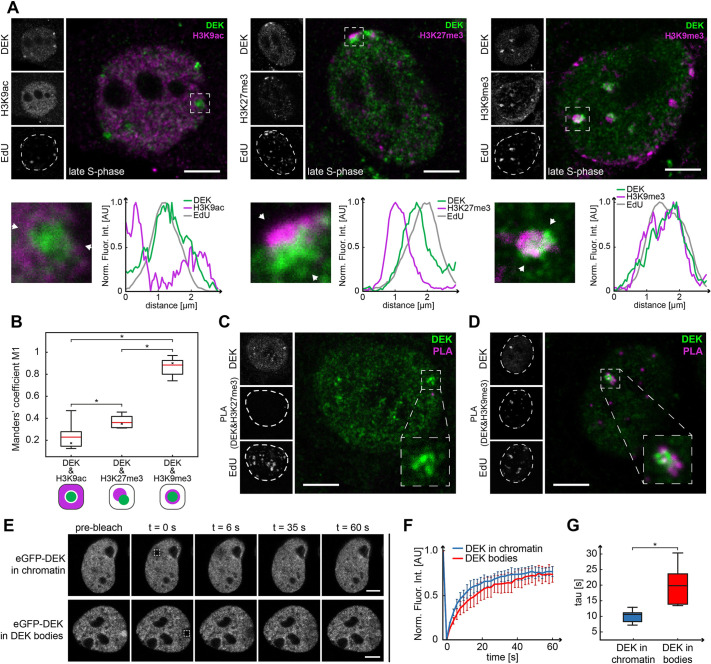
**DEK bodies position within constitutive heterochromatin regions in late S-phase.** (A) Representative confocal images of late S-phase MCF10A cell nuclei labeled with EdU and antibodies specific for DEK (Santa Cruz Biotechnology) and for three post-translational histone modifications (left, H3K9ac; center, H3K27me3; right; H3K9me3). ROIs containing DEK bodies are marked by a dashed box and displayed as magnified images below. The fluorescence intensity profiles were calculated along the lines delimited by the white arrowheads and normalized to 1. AU, arbitrary units. Dashed lines in EdU images indicate the edge of the nucleus. Scale bars: 5 µm. (B) Analysis of colocalization of DEK bodies (29 for DEK and H3K9ac, 37 for DEK and H3K27me3, and 35 for DEK and H3K9me3) and chromatin marks using Manders' coefficients. In the box plots, the red line indicates the mean value, the asterisk marks the median, the box boundaries represent the interquartile range, and the whiskers above and below the box indicate minimum and maximum values. Diagrams on the bottom schematically represent localization pattern. Differences between conditions were calculated by an unpaired two-tailed *t*-test after establishing the variance differences by *F*-test, with the application of post-hoc Bonferroni correction: *P=0.0078368 (H3K9ac and H3K27me3), *P=4.37904×10^−7^ (H3K9ac and H3K9me3), *P=9.19623×10^−8^ (H3K27me3 and H3K9me3). (C) Confocal image of a late S-phase MCF10A cell nucleus after PLA with antibodies specific for DEK (Santa Cruz Biotechnology) and H3K9me3. The PLA positive signal (magenta) indicates direct interaction. Analysis of 46 DEK bodies in 26 cells from two biological replicates indicated that 86% of DEK bodies showed positive PLA signal. Scale bar: 5 µm. (D) Confocal image of a late S-phase MCF10A cell nucleus after PLA with antibodies specific for DEK (Santa Cruz Biotechnology) and H3K27me3. Analysis of 13 DEK bodies in five cells from two biological replicates indicated that 0% of DEK bodies showed positive PLA signal. Dashed lines in PLA and EdU images indicate the edge of the nucleus. Scale bar: 5 µm. (E) FRAP analysis of mobility of eGFP–DEK that was transiently expressed in MCF10A cells in bulk chromatin and in DEK bodies. The bleached ROIs (2 µm×2 µm) are indicated by dashed squares and were selected based on location. Fluorescence recovery was monitored for 3 min. Scale bar: 5 µm. (F) DEK displays a lower mobility in DEK bodies than in bulk chromatin. Normalized FRAP recovery curves based on the data from E. Data points show the average of six experiments for DEK bodies and 14 experiments for DEK in chromatin. Error bars show the s.e.m. (G) Halftimes of recovery for eGFP–DEK in chromatin (τ½=10.6±3.1 s; mean±s.d.) and in DEK bodies (τ½=17.1±7 ; mean±s.d.) calculated from the FRAP data in F. In the box plots, the line indicates the median, the box boundaries represent the interquartile range, and the whiskers indicate minimum and maximum values. *P=0.009 (unpaired two-tailed *t*-test).

The occurrence of DEK in a subpopulation of PCNA- and EdU-positive nuclear bodies in late S-phase let us hypothesize that DEK accumulates at sites of replicating heterochromatin. To test this hypothesis, we conducted immunofluorescence analyses of three well-established chromatin markers in S-phase cells – H3K9ac for euchromatin (Wang et al., 2008), H3K27me3 for facultative heterochromatin (Trojer and Reinberg, 2007), and H3K9me3 for constitutive heterochromatin (Nakayama et al., 2001) (Fig. 3A,B; Fig. S3A–C). We observed that within DEK bodies, DEK-specific signals had little if any overlap with H3K9ac-labeled chromatin, were spatially juxtaposed to H3K27me3 signals and colocalized with H3K9me3 (Fig. 3A), strongly suggesting that DEK bodies represent regions of replicating constitutive heterochromatin. This conclusion was corroborated by Manders' coefficient analysis – a variation of the Pearson's correlation coefficient – yielding the highest overlap for DEK and H3K9me3. The values for the coefficient M_1_ indicating the fraction of DEK bodies containing the different modified histones were as follows: M_1(DEK&H3K9me3)_=0.87±0.09, M_1(DEK&H3K9ac)_=0.22±0.12; and M_1(DEK&H3K27me3)_=0.37±0.06 (mean±s.d.). The analysis was performed on 21 DEK bodies in three independent experiments (Fig. 3B). Further evidence was obtained with a proximity ligation assay (PLA), which confirmed that there was direct interaction of DEK with H3K9me3 in 86% of DEK bodies, but not with H3K27me3 (Fig. 3C,D). Fluorescence recovery after photobleaching (FRAP) in DEK bodies that were selected based on localization at the nuclear periphery (see below) furthermore showed a reduced mobility of DEK in the bodies with respect to the bulk chromatin (Fig. 3E–G). In line with these data, DEK bodies were also predominantly associated with heterochromatin, in particular with H3K9me3, in U2-OS wild type and U2-OS KI eGFP-DEK cells (Fig. S4).

Finally, we made a qualitative assessment of the localization of DEK bodies with respect to known heterochromatic compartments such as centromeres, the Barr body and the nuclear periphery (Fig. S5). Maximum intensity projections of confocal *Z*-stacks revealed that the epigenetic mark for centromeres, the histone H3 variant CENP-A, reproducibly decorates DEK bodies from both sides (Fig. S5A). We did not observe spatial proximity of DEK and CENP-A outside of late S-phase (Fig. S5B). A staining pattern of similar symmetry was observed for *Xist* RNA, a selective marker for the Barr body (Cerase et al., 2015) (Fig. S5C). No such pattern was observed in male BPH-1 cells (data not shown). Similarly, we found DEK bodies in close proximity to the nuclear periphery and lamin-A-positive nuclear invaginations only in S-phase (Fig. S5D,E). Furthermore, DEK bodies corresponded to regions of dense chromatin, as shown by DNA staining with ToPro3 (Fig. S5F). Altogether, our collective microscopy data support a role for DEK bodies in the replication of heterochromatic chromatin regions in late S-phase.

### DEK body dynamics is affected by DNA replication inhibitors

**Fig. 4.**
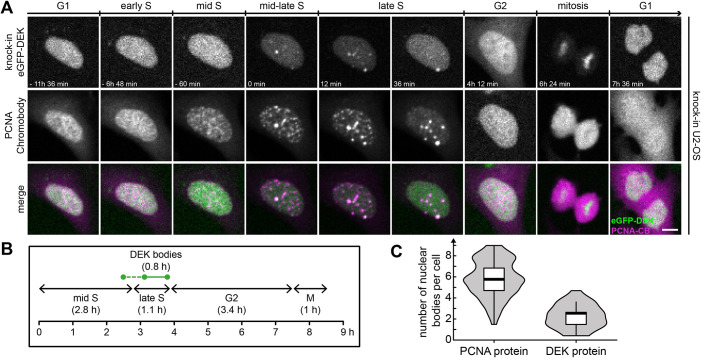
**DEK bodies form during late S-phase of the cell cycle.** (A) Time-lapse fluorescence microscopy of U2-OS KI eGFP–DEK cells transiently expressing mRFP–PCNA. Images were acquired at a spinning disk confocal microscope. The figure shows a representative image sequence. Magenta, PCNA chromobody; green, eGFP–DEK. Scale bar: 5 µm. (B) Mean durations of cell cycle phases and mean DEK body lifetime. A total of 56 cells were scored manually from two independent experiments. Mid S, late S, G2 and M-phases were identified according to the PCNA localization pattern. Only images with clearly detectable DEK bodies were scored as positive. DEK bodies appear reproducibly in late S-phase (continuous green line), and sporadically also in mid-S-phase (dashed green line). (C) Quantification of PCNA body and DEK body numbers in late S-phase using the same data set as in (B) (see text for details). Only PCNA or DEK bodies were included in the evaluation that persisted for at least two consecutive frames. The violin plot shows the density distribution of the data, the black line indicates the median, the box the interquartile range (IQR) and the whiskers 1.5× the IQR value from the 25th and 75th percentiles.

Having assessed that DEK bodies are implicated in the replication of heterochromatin in fixed cells, we were interested in exploring and manipulating their dynamics in living cells. To this end, we transiently expressed mRFP–PCNA in U2-OS KI eGFP-DEK cells via a PCNA chromobody plasmid and performed time-lapse imaging experiments using a spinning disk confocal microscope (Fig. 4A). DEK body formation was evaluated manually and occurred reproducibly in every cell in late S-phase. 2.7±1.0 DEK bodies (mean±s.d.) formed per cell and persisted for 48±30 min (mean±s.d.) until G2 entry (Fig. 4B,C). 78±15% (mean±s.d.) of DEK bodies colocalized with PCNA-positive replication foci, whereas 11±4% of the latter contained DEK (DEK bodies, *n*=37; PCNA foci, *n*=254; seven cells from two independent experiments were analyzed). A similar tendency was observed when assessing colocalization of DEK bodies with EdU. 90±18% of DEK bodies colocalized with EdU-positive regions, whereas only 17±8% EdU regions colocalized with DEK bodies (38 DEK bodies and 218 EdU regions analyzed in 13 cells in two independent experiments). A similar behavior was observed also in MCF10A cells transiently expressing fluorescent fusions of DEK and PCNA, with a higher number of DEK bodies (6.7±2.2) that persisted for a longer time (189±38 min). 82±18% of DEK bodies colocalized with PCNA-marked replication foci in these cells (*n*=19 DEK bodies) (Fig. S5G).

**Fig. 5.**
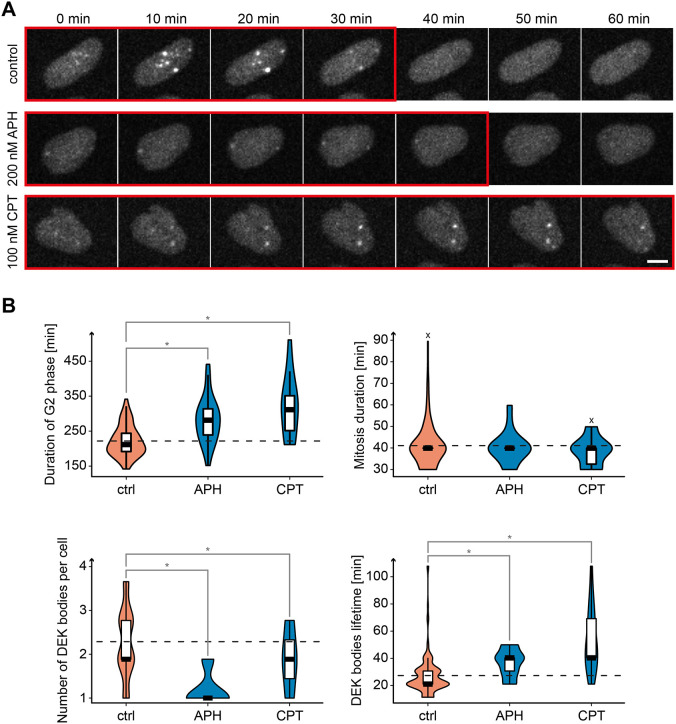
**Formation of DEK bodies is dependent on DNA replication.** (A) Time-lapse fluorescence microscopy of U2-OS KI eGFP-DEK cells treated for 1 h with either 200 nM APH or 100 nM CPT, or left untreated (control). Images were acquired for 24 h with a spinning disk confocal microscope. A representative time series is shown for each treatment condition. Frames with DEK bodies are highlighted by a red outline. Scale bar: 10 µm. (B) Quantification of G2-phase and mitosis duration, DEK body number and lifetime in time series data as in A. The violin plots show the density distribution of the data points, the black line indicates the median, the box the interquartile range (IQR) and the whiskers 1.5× the IQR value from the 25th and 75th percentiles. (×) The control and CPT data sets contained one outlier each, which was omitted from the quantification. Control, 87 cells from seven replicates; CPT, 15 cells from four replicates; APH, 28 cells from four replicates. Data analysis was carried out by performing single-factor ANOVA. The statistical significance of the difference between control, APH-treated and CPT-treated cells was calculated using an unpaired two-tailed Student's *t*-test with a post-hoc test (Bonferroni correction) after assessing the variance (*F*-test). *P<0.017.

To determine whether impairment of DNA replication would affect DEK body formation, we employed aphidicolin (200 nM) and camptothecin (100 nM), two well-established replication fork inhibitors acting by different mechanisms, which at these low doses slow down but do not arrest replication (Fig. 5A) (Deutzmann et al., 2015; Vesela et al., 2017). The treatments did not affect viability, but triggered accumulation of cells in G2, followed by normal mitoses (Fig. 5B, upper panels). Of the two substances, aphidicolin showed the most prominent effect reducing DEK body number by 47%, and prolonging DEK body lifetime by 28%. Camptothecin had a similar but more moderate effect (Fig. 5B, lower panels). This response strongly suggests that DEK bodies are implicated in the replication of DNA structures particularly susceptible to aphidicolin, such as common fragile sites, which in turn can coincide with highly repetitive heterochromatic regions like centromeres or ribosomal DNA (Lukusa and Fryns, 2008), in line with our data on the colocalization of DEK bodies with heterochromatin histone marks.

### Downregulation of the SUMO pathway increases DEK body number in an siRNA screen for DEK body regulators

As our time-lapse microscopy experiments could measure the effect of mild replication stress on DEK body formation, we chose to perform an unbiased imaging-based approach to identify DEK body regulatory factors. To this end, we set up a high-throughput siRNA screen targeting genes from a published DEK interactome (Smith et al., 2018), DEK-related factors derived from the literature and genes selected from a commercial library targeting the DNA damage response that responded to the gene ontology terms ‘DNA replication’ and ‘S-phase’. The final library consisted of 332 genes (678 siRNAs total, at least two siRNAs per gene; Table S1).

**Fig. 6.**
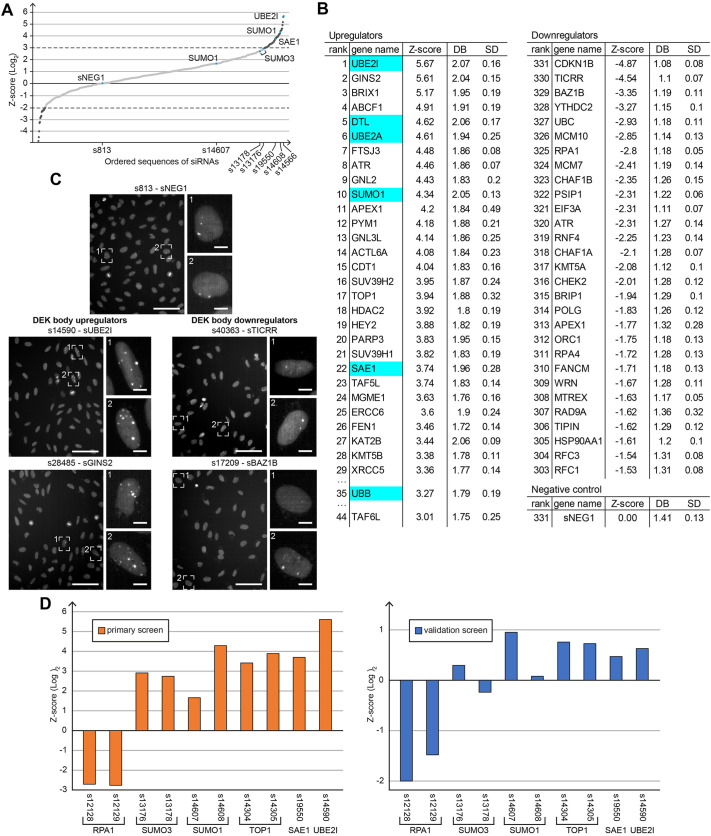
**An siRNA screen identifies positive and negative regulators of DEK body formation.** (A) Plot of the log2-transformed ranked mean *Z*-score data of the primary screen. The screen was performed on four field of view per well, with two wells per siRNA (for each of two replicates), yielding 16 *Z*-scores for each siRNA. Dark gray dots, top scorer (3≤*Z*-score≤−2); blue dots, target genes included in the pilot screen. sNEG1, negative control siRNA. (B) List of the strongest DEK body upregulating and downregulating genes based on the *Z*-score of the corresponding siRNAs. For the upregulators, ranks 30–34 and 36–43 are not shown for better readability (for a complete list see Table S1). Mean number of DEK bodies (DB) and standard deviation (SD) are shown. Highlighted are genes belonging to the SUMO pathway. (C) Representative widefield images from the primary screen showing U2-OS KI eGFP-DEK transfected with a negative control siRNA sNEG1 (s813) and siRNAs targeting the two strongest DEK body upregulating (left) and downregulating (right) genes. Magnified insets with DEK body-positive nuclei are shown. Scale bars: 100 µm (main images); 10 µm (magnifications). (D) Comparison of *Z*-score data from the primary and the secondary (validation) screen (for a complete list see Table S3). The log2-transformed mean *Z*-score for the indicated siRNAs is shown. The primary screen was performed in duplicate, the validation screen in triplicate. Knockdown efficiency of SUMO1 and SUMO 3 siRNAs was confirmed by western blotting (Fig. S7A,B).

Experimental conditions and a suitable image analysis pipeline using CellProfiler software were established in two pilot screens including aphidicolin and camptothecin as positive controls (see Material and Methods, Table S2 and Fig. S6). The primary screen targeting the chosen gene panel was performed in duplicate and a *Z*-score of the log-transformed mean number of DEK bodies per cell was calculated for each siRNA (Fig. 6A). This resulted in 51 candidate siRNAs enhancing (upregulators) and 29 candidates reducing DEK body numbers (downregulators) corresponding to 44 and 29 different genes, respectively (Fig. 6B,C). Gene ontology (GO) analysis of the upregulators revealed significant enrichment for siRNAs targeting histone acetyl- and methyl-transferases and SUMO or ubiquitin pathway proteins. Acetyltransferases KAT2B, TAF5L and TAF6L are part of PCAF, the p300/CBP-associated factor complex, whereas ACTL6A is part of NuA4 and several SWI/SNF-like nucleosome remodeling complexes. The histone deacetylase HDAC2, a member of the family that catalyzes the reverse reaction, and the methyltransferases SUV39H1 and SUV39H2, which are responsible for H3K9me3, were also among the hits. By contrast, most downregulators could defined as cell cycle regulators, such as proteins involved in G1/S transition [the CDK2–cyclinE inhibitor CDKN1B, the MCM helicase component MCM7, the pre-initiation complex (pre-IC) component TICRR and the ssDNA binder RPA1], or G2/M checkpoint proteins like CHEK2, BRIP1 and ORC1. Notably, UBC is, as is the DEK body upregulator UBB, a ubiquitin precursor. Another interesting downregulator is RNF4, as it connects the ubiquitylation and SUMOylation pathways. Secondly, from the ubiquitin modification pathway, siRNAs targeting ubiquitin precursor UBB, the E2 conjugating enzyme UBE2A and DTL, a substrate-specific adaptor of the E3 ligase CUL4A-DDB1, were found as top hits. Of note, UBC, another ubiquitin precursor, appeared as a downregulator. From the SUMOylation pathway, small ubiquitin-related modifier 1 (SUMO1), E1 activating enzyme SAE1 and E2 enzyme UBE2I were among the top hits. Interestingly, two SUMO3 siRNAs (rank 54 and 62) were also among the major upregulators. From seven SUMO-related siRNAs included in this screen (SUMO1–SUMO4, SAE1, UBE2I and SENP3), only three (SUMO2, SUMO4 and SENP3) were not among the top 10% scoring siRNAs.

The number of DEK bodies per cell was also the most sensitive and robust readout for DEK body formation in the automated approach, reproducing the results from the manual experiments (Fig. S6D). To further confirm the identified hits, we conducted a low-throughput secondary screen with 28 siRNAs (Fig. 6D; Table S3). In general, the values for the *Z*-score obtained in this secondary screen were smaller and more variable, most likely due to the different, less automated imaging system and the manual dispensing of transfection reagents. Still, the effect of up and downregulation of DEK body number was largely confirmed (Fig. 6D). Taken together, downregulation of post-translational modification pathways, especially lysine acetylation and SUMOylation, seemed to upregulate DEK bodies. On the other hand, knockdown of proteins involved in cell cycle transitions led to a decreased DEK body count. Importantly the secondary screen validated four top DEK body upregulators from the primary screen belonging to the SUMO pathway, among them the E1 activating enzyme SAE1 and the E2 conjugating enzyme UBE2I. Our screening approach thus determined that DEK body formation is subject to regulation by multiple post-translational modifications, with a major involvement of the SUMO and ubiquitin pathway.

### DEK is a SUMO target and SUMOylation is required for the formation of DEK bodies

**Fig. 7.**
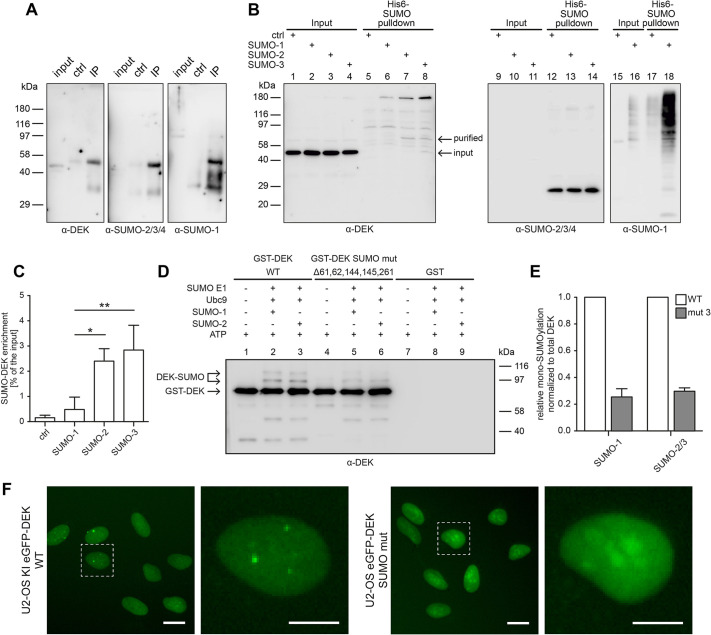
**DEK is SUMOylated in cells and SUMOylation is involved in the formation of DEK bodies.** (A) Immunoblot analysis of co-immunoprecipitations (IP) performed using polyclonal rabbit anti-DEK antibody (K-877 Ab) from U2-OS whole-cell lysates. For the control samples rabbit IgG Isotype control was used. Immunoblot detection was achieved using a Veriblot for IP secondary antibodies, only recognizing native antibodies therefore not detecting denatured heavy and light immunoglobulin chains. (B) Immunoblot analysis of His–SUMO pulldown experiments from HeLa cells expressing 6×His–SUMO1, 6×His–SUMO2, 6×His–SUMO3 or control cells after nickel affinity chromatography. Input lane: 1% of total cell lysate. Purified proteins were analyzed with DEK (K877 Ab) and SUMO-specific antibodies. SUMO-2/3/4, SUMO2, SUMO3 and SUMO4. Images in A and B representative of three experimental repeats. (C) Densitometric quantification of mono-SUMOylated DEK signals from B (left panel). DEK signals in pulldown samples were normalized to the corresponding input samples. Input was set to 100% and the ratio of indicated pulldown to input signal was calculated. Shown are mean values of three experiments, error bars represent the standard deviation. **P<0.01, *P<0.05 (one-way ANOVA with Bonferroni post-test). (D) *In vitro* SUMOylation assay. Immunoblot analysis of *in vitro* SUMOylation reactions containing recombinant GST-tagged DEK wild-type (WT), mutant, or GST only proteins as indicated and using DEK-specific antibodies for detection. (E) Densitometric quantification of mono-SUMOylated DEK from *in vitro* samples as in D. SUMO-2/3, SUMO2 and SUMO3. Shown is the mean of three experiments. Error bars indicate the s.d. (F) Representative still images from a widefield time-lapse microscopy movie of U2-OS KI eGFP-DEK or a cell line stably expressing a wild-type or a SUMOylation-deficient DEK mutant [DEKmut (Δ61,62,144,145,261)] cells. Representative of two experimental repeats. Scale bars: 20 µm.

As both screening approaches showed that downregulation of the SUMOylation pathway resulted in the upregulation of the number of DEK bodies in U2-OS KI eGFP-DEK cells, we hypothesized that DEK may be a target of SUMOylation and that, in turn, its SUMOylation state, potentially in conjunction with other post-translational modifications, may modulate the ability to form DEK bodies in late S-phase. Indeed, the DEK protein sequence contains a SUMOylation consensus motif ψ-K-X-E/D (Zhao et al., 2014) at lysine 261 (AKRE, http://jassa.fr) and DEK has been shown to be SUMOylated under stress conditions in a mass spectrometry study of H_2_O_2_-treated SH-SY5Y neuroblastoma cells (Grant, 2010). To test whether DEK can be SUMOylated under steady-state conditions, we focused on SUMO1 and SUMO3, as both were strong hits in the screens. We first conducted co-immunoprecipitations using anti-DEK antibodies, showing direct interaction of DEK with SUMO1 and an antibody that detects SUMO2 and SUMO3 (SUMO2/3) (Fig. 7A). To narrow down a specific SUMO paralog targeting DEK, we used HeLa cells stably expressing 6×His-SUMO1, 6×His–SUMO2 or 6×His–SUMO3 allowing for subsequent nickel affinity purification of SUMOylated factors. This approach revealed modification of DEK by all SUMO forms, yet with the strongest activity for SUMO3 (which also generates multimers) (Fig. 7B,C). As we could confirm the existence of SUMOylated DEK in cells under physiological conditions, we were next interested to map the specific SUMOylation sites in the DEK primary sequence. As indicated above, DEK harbors one SUMOylation consensus motif at position lysine 261. However, mutation of this site did not yield a substantial reduction in SUMOylation in *in vitro* SUMOylation assays using recombinant GST-tagged DEK as acceptor (Fig. S7C). Therefore, we screened the DEK sequence for additional potential SUMOylation sites using the prediction server at http://jassa.fr/, which revealed amino acids 61, 62, 144 and 145 as putative SUMO attachment sites. Interestingly, mutation of these sites individually, as was the case with 261, did not result in a substantial reduction of SUMOylation activity on DEK (Fig. S7C). However, DEK molecules carrying mutations at all five sites showed an 80% reduction in SUMOylation (Fig. 7D,E), highlighting that most of the potential SUMOylation sites were captured by our mutational approach. Given that we had created a DEK mutant that is substantially less susceptible to SUMOylation under our experimental conditions, we then investigated the ability of such a mutant to form DEK bodies in cells. Therefore, we established a stable cell line expressing this mutant as eGFP fusion and monitored the occurrence of DEK bodies as described above. No DEK body formation was observed in cells expressing SUMO-less DEK (GST-DEK SUMO mut; Fig. 7F, Movies 1 and 2). Interestingly, this mutant retained full DNA binding activity *in vitro* as shown by electrophoretic mobility shift assay (EMSA) analysis but displayed an altered chromatin association behavior in cell fractionation experiments (Fig. S8A). Non-SUMOylated DEK seems to bear other, yet to be identified, post-translational modifications as indicated by the appearance of high molecular mass species in cytosolic fractions of cells expressing this mutant (Fig. S8B). Together, our results uncover a yet unrecognized link between SUMO and DEK, and points at a complex role of SUMOylation in the regulation of DEK bodies, where reducing the cellular capacity for SUMO modification increases the number of DEK bodies, whereas non-SUMOylatable DEK appears not to form bodies. Given that DEK is an established target of various post-translational modifications, that multiple post-translational modification pathways were identified in our screen for DEK body regulators and that absence of SUMO alters the modification and chromatin binding pattern of DEK, we propose that SUMO is a novel player in the network of DEK modifiers involved in regulating its function during DNA replication. The mode of action warrants further investigation.

## DISCUSSION

### DEK bodies reflect the formation of transient DEK multimers in the nucleus during late DNA replication

We report on the discovery of DEK bodies, DNA replication-dependent focal assemblies of the oncoprotein DEK in the cell nucleus. DEK bodies were observed in every cell during mid-late S-phase in primary and immortalized cells, and to a lesser extent in transformed cell lines. This finding was somewhat surprising as the large majority of immunofluorescence studies have described DEK as a factor dispersed in an uniform punctuate pattern in the nucleus (Hu et al., 2007; Kappes et al., 2001; Matrka et al., 2015). Intriguingly, the existence of non-covalent DEK multimers was proposed in the past based on biochemical analyses of DEK–chromatin complexes (Kappes et al., 2004b), but had eluded demonstration in live cells so far. We interpret DEK bodies as the cellular correlate of these non-covalent multimers, whose detection by microscopy was most likely facilitated by fusion of DEK to GFP. This reporter has a propensity to form aggregates at high concentrations (Stepanenko et al., 2021) and we assume that, in our setting, it contributed to slow down the dynamics of DEK bodies and made them amenable to live-cell microscopy imaging. The focused inspection of high resolution immunofluorescence images revealed that DEK bodies are distinct sites of active replication containing endogenous DEK in close spatial proximity to nascent DNA and the replication processivity factor PCNA, consistent with previous iPOND analyses (Alabert et al., 2014; Aranda et al., 2014; Garcia et al., 2017; Lossaint et al., 2013; Ribeyre et al., 2016; Sirbu et al., 2013). The number and persistence of DEK bodies varied substantially between MCF10-A and U2-OS cells, with the former showing a more pronounced expression of these bodies. Unfortunately, a direct quantitative comparison of DEK bodies between these two cell lines is not appropriate as DEK was expressed endogenously in U2-OS cells and transiently in MFC-10A cells, and imaging was performed with different microscopes in different laboratories. However, in both cases, DEK bodies were sparingly observed in mid S-phase, probably reflecting cell-to-cell variability in replication timing (Zhang et al., 2017).

Previous data suggesting that DEK may occur in defined nuclear substructures included coimmunoprecipitation and spurious colocalization with PML (Ivanauskiene et al., 2014), and the redistribution to interchromatin granule clusters in the presence of deacetylase inhibitors (Clarey, 2005; McGarvey et al., 2000), but no link to the cell cycle or DNA replication was made in these studies.

In general, the formation of nuclear bodies has emerged as an organizing principle for the orchestration of genome-associated functions in time and space. Different nuclear bodies have been described in which proteins, often together with RNAs, are sequestered and released according to demand, including the nucleolus, nuclear speckles and paraspeckles, PMLs, Cajal bodies, 53BP1 bodies and other bodies (Dundr, 2012). Post-translational modifications, as well as weak intermolecular interactions mediated by intrinsically disordered protein domains, seem to play a fundamental role in the genesis and dynamic composition of these membrane-less nuclear condensates. It is thus not surprising that DEK, a DNA and RNA-binding protein carrying several post-translational modifications and an intrinsically disordered region (amino acids 170–270) is prone to forming nuclear bodies.

### DEK bodies form at replicating heterochromatic regions

Our data provide strong evidence that DEK bodies are sites of heterochromatin replication. These include their narrow window of appearance during late S-phase, the association with the heterochromatin histone marker H3K9me3, the high level of chromatin compaction and the reduced mobility of DEK in bodies, and the local proximity to the Barr body and centromeric chromatin marked by CENP-A. The lack of colocalization between CENP-A and DEK reported previously (Ivanauskiene et al., 2014) was most likely due to the fact that endogenous DEK bodies are transient structures visible only in late S-phase, and they might have thus escaped detection.

The reproducibility with which DEK bodies form in consecutive cell cycles supports the notion that they reflect a conserved and necessary passage in genome duplication. The striking appearance of the centromeric and *Xist*-specific signals as dots decorating DEK bodies (Fig. S5A,C) are suggestive of newly replicated chromosomes tethered to these bodies at regions rich in the secondary structures that are preferred DEK substrates *in vitro*, and that are difficult to replicate and prone to breakage. In line with this observation, downregulation of DEK resulted in the formation of anaphase bridges, the appearance of DNA breaks on mitotic chromosomes and of 53BP1 bodies in daughter cells (Deutzmann et al., 2015). Although we described how DEK affects DNA replication globally in the Deutzmann et al. (2015) study, the results obtained here suggest a more specialized function of the protein, at specific genomic regions including the centromere and possibly other constitutive heterochromatic regions. These findings might explain the relatively mild effects of DEK downregulation on replication fork progression, as assessed by bulk fiber assays. The assessment of the influence of DEK on the processivity of the replication fork therefore awaits DNA combing experiments coupled with the detection of heterochromatic fibers.

### DEK bodies are novel elements of a multilayered signaling system regulating heterochromatin replication that involves SUMO and other post-translational modifications

siRNAs that upregulate the DEK body numbers show a remarkable enrichment in proteins involved in post-translational modification pathways. The histone acetyltransferases (HATs) ACTL6A, KAT2B, TAF5L and TAF6L, the histone deacetylase HDAC2, the histone methyltransferases SUV39H1 and SUV39H2, and SUMOylation-related proteins UBE2I, SUMO1 and SAE1 are among the top hits. Acetylation of DEK by KAT2B (a PCAF component) has been shown previously (Cleary et al., 2005). In that study, overexpression of KAT2B led to an accumulation of DEK in interchromatin granule clusters (IGCs), which are nuclear domains enriched in pre-mRNA splicing factors. Pharmacological inhibition of KAT2B in turn, blocked the relocation of DEK into IGCs, which is opposite to the effect of downregulation of KAT2B on DEK bodies. This finding further underscores that DEK bodies are distinct from ICGs, which are more abundant (20–30 clusters per cell) and are stable during the whole of interphase (Mintz et al., 1999).

Here, we focused on SUMOylation as a post-translational modification that has recently emerged as a major regulator of heterochromatin formation and replication. We substantiate the result from the siRNA screen by showing that DEK is modified by SUMO moieties in cells, and that SUMO-less DEK does not form bodies. However, as creating SUMO-less DEK required mutation of five amino acids we cannot exclude at this moment that additional post-translational modifications that contribute to the inability of this DEK mutant to form bodies might be affected as well. Still, some hypotheses can be formulated based on the current knowledge about the multilayered regulation of DNA replication through an intricated network of post-translational modifications (see Abbas, 2021). Recent data show, for example, that SUV39H1 promotes SUMOylation and *de novo* targeting of HP1α to pericentric heterochromatin, independently of its methyltransferase activity. The latter is required in a second step to maintain and propagate heterochromatin (Maison et al., 2016). This knowledge aligns well with our screening data, where downregulation of both SUV39H1 and SUMO increase the number of DEK bodies, potentially suggesting that DEK could play a role in ‘seeding’ constitutive heterochromatin after DNA replication (Maison et al., 2016). Our data also support and refine the conclusions from our previous study showing that DEK is essential for heterochromatin integrity, and has a Su(var)-like activity by enhancing HP1α binding to H3K9me3 (Kappes et al., 2011a).

Heterochromatin preferentially harbors repetitive sequences causing non-B DNA secondary structures. Their replication requires specialized polymerases that can sustain fork progression in the presence of difficult-to-replicate regions, but on the other hand lack proofreading activity and are therefore error-prone and mutagenic (reviewed in Tsao and Eckert, 2018). The switch from replicative polymerase to these specialized, or translesion polymerases, at the replisome is regulated by ubiquitylation and SUMOylation (Despras et al., 2016; Mailand et al., 2013). In particular, SUMOylation is required on the one hand, for driving specialized polymerases to nascent DNA while, on the other hand, enabling discharge from the replisome after bypass of the problematic site to limit replication errors and prevent mutagenesis, as shown for Pol η (Despras et al., 2016; Guérillon et al., 2020). In this context, it can be hypothesized that DEK in bodies participates in the maturation of nascent heterochromatin after replication involving specialized polymerases and that this step might require SUMOylation of DEK. Secondly, the increase in the number of DEK bodies observed after downregulation of the SUMO pathway could be a consequence of impaired turnover of these specialized replisomes and the replacement by proof-reading replicative polymerases. Interestingly, it has also been reported that Polη is SUMOylated at multiple sites which can substitute for each other, so that simultaneous mutation of all SUMOylation sites is necessary to achieve dysregulation of the Polη dynamics (Guérillon et al., 2020).

At present, we have no straightforward interpretation for the reduction in DEK bodies that seems to result from increased DNA replication stress, as highlighted by the lack of DEK bodies in highly cancerous MDA-MB-231 cells and in the presence of aphidicolin and, to a lesser extent, camptothecin. We speculate that, under these conditions, replisomes at difficult-to-replicate-regions would be more likely to collapse leading to DNA breakage before chromatin maturation, thus impairing DEK body assembly. Accordingly, we found increased γH2AX foci after DEK downregulation in a previous study (Kappes et al., 2008). The propensity for DNA strand breaking is a well-established feature of difficult-to-replicate-regions such a common fragile sites (CFS), and their stability has been shown to depend on specialized polymerases (Bergoglio et al., 2013; Bhat et al., 2013).

In conclusion, the link established here between DEK and the establishment of constitutive heterochromatin during replication sheds new light on the mechanisms of action of this so far enigmatic oncoprotein. Some of its pleiotropic effects might be associated with the seeding of (constitutive) heterochromatin, a process that broadly impacts the epigenetic landscape of the cell and its transcriptional program and is essential for maintaining genomic stability (Penagos-Puig and Furlan-Magaril, 2020). We expect these effects to be regulated by the concerted actions of multiple post-translational modification pathways and provide evidence here for SUMOylation as one prominent example awaiting further investigation.

## MATERIALS AND METHODS

### Cell culture

Mammary epithelial, non-transformed cell line MCF10A (ATCC CRL-10317) was grown in Dulbecco's modified Eagle's medium:nutrient mixture F-12 (DMEM:F-12; 1:1) medium (Gibco, 11330057) supplemented with 5% horse serum (HS, Thermo Fisher Scientific, 16050122), 2 mM L-glutamine (Sigma-Aldrich) and 1% penicillin-streptomycin (Sigma-Aldrich, G6784), 10 µg/ml insulin (Sigma-Aldrich, I9278), 0.5 µg/ml hydrocortisone (Sigma-Aldrich, H0888), and 20 ng/ml of human epidermal growth factor (hEGF, Sigma-Aldrich, E9644) added freshly before use. MCF7 cells and MDA-MB-231 cells (ATTC HTB-26) were grown in DMEM (Gibco, 11330057) supplemented with 10% fetal bovine serum (FBS, Euroclone, ECS0180L), 1% penicillin-streptomycin (Sigma-Aldrich, G6784) and 2 mM L-glutamine. Cells were grown on 10 cm^2^ dishes (about 10–12 cell passages) at 37°C in 5% CO_2_. For any experiments on fixed cells, cells were plated on glass coverslips coated with 0.5% (w/v) pork gelatine (Sigma-Aldrich, G2500) dissolved in phosphate-buffered saline (PBS) and autoclaved. U2-OS osteosarcoma (a kind gift from G. Marra, University of Zurich, Switzerland) and U2-OS KI eGFP-DEK cells (described in Ganz et al., 2019) were cultured in McCoy's 5a modified medium (Gibco, Life Technologies) supplemented with 10% FBS (Capricorn Scientific), 100 U/ml penicillin and 100 µg/ml streptomycin (both Gibco, Life Technologies). For selection after transfection, Geneticin (Thermo Fisher Scientific) was added at concentrations of 200 µg/ml for U2OS KI eGFP-DEK WT, eGFP-DEK SUMOmut Δ61,62, eGFP-DEK SUMOmut Δ261 and eGFP-DEK SUMOmut Δ61,62,144,145,261 cells. During all experiments using siRNA-mediated protein downregulation penicillin and streptomycin were omitted from the medium. HeLa S3 cervical adenocarcinoma cells and HeLa 6×His-SUMO cells (a kind gift from Ronald T. Hay, University of Dundee, UK) were cultured in DMEM (Gibco, Life Technologies) supplemented with 10% FBS (Capricorn Scientific), 100 U/ml penicillin, 100 μg/ml streptomycin (both Gibco, Life Technologies) and 6 mM L-glutamine (Gibco, Life Technologies). hTERT immortalized BJ-5ta foreskin fibroblasts were cultured in a 4:1 mixture of DMEM and Medium 199 (Gibco, Life Technologies) supplemented with 10% FBS, 4 mM L-glutamine and 10 µg/ml hygromycin B (Calbiochem).

### Transfection and cell sorting

For detection of endogenous PCNA, U2-OS KI eGFP-DEK or MCF10A cells were transfected with a PCNA-specific chromobody expression vector (pCCC-TagRFP, Chromotek) using Effectene (Qiagen U2-OS KI eGFP-DEK) or Lipofectamine 3000 (Invitrogen) according to the manufacturer's instructions. For U2-OS KI eGFP-DEK cells, the medium was exchanged 4 h after transfection and the cells were incubated for a further 20 h before imaging. For MCF10A cells, the medium was exchanged about 12 h post-transfection, and experiments were carried out between 24 h and 48 h after transfection. For the generation of U2-OS cell lines stably expressing eGFP–DEK mutants, pEGFP-N1-hDEK plasmids encoding the mutated DEK versions were transfected into U2-OS DEK knockout cells using Lipofectamine 3000 reagent (Invitrogen). At 3–9 days after transfection (depending on cell growth and viability) selection was carried out with geneticin at a final concentration of 200 µg/ml. To create cell lines with a uniform GFP fluorescent signal, the stable transfected cell lines were bulk sorted for a moderate fluorescence signal using a FACSAria IIIu Cell Sorter (BD Biosciences).

### Immunochemical methods

All antibodies used in this study are listed in Table S4.

### Confocal and widefield microscopy

#### Immunofluorescence and nascent DNA staining

For detection of DNA synthesized during DNA replication in MCF10A cells, a DNA precursor (EdU, 10 µM) was added to the cell culture medium for 25 min, the cell cultures were washed three times with pre-warmed PBS, and fixed with formaldehyde (3.7%, methanol-free). After the subsequent blocking with the blocking buffer [composed of 0.1% Triton X-100 and 3% (w/v) of bovine serum albumin (BSA)] for 1 h at room temperature (RT), EdU was detected following the manufacturer instructions (Click-iT EdU Imaging Kit or Click-iT EdU PLUS Imaging Kit, Molecular Probes with Alexa Fluor 647 or Alexa Fluor 594 azide dye). Primary antibodies were diluted in the blocking buffer and the samples were incubated for 2 h at RT and overnight at 4°C, rinsed with the blocking buffer again and incubated with the secondary antibodies for 1 h at RT, diluted in PBS. After the incubation with secondary antibodies, cells were rinsed three times with PBS, and incubated for 20 min at RT with TO-PRO™-3 iodide (Invitrogen, 1:2000) when necessary, and again rinsed three times with PBS. The samples were mounted in ProLong™ Diamond Antifade Mountant (Invitrogen, P36961). Confocal images depicting MCF10A cells were acquired with a Leica TCS SP5 confocal laser-scanning microscope, using an HCX PL APO 100×/1.40/0.70 oil immersion objective lens (Leica Microsystems, Mannheim, Germany). Excitation was provided with a white laser, enabling the best condition as function of the utilized dye, namely 488 nm (for Alexa Fluor 488), 532 nm (for Atto 532), 554 nm (for Quasar 579), 594 nm (for Alexa Fluor 594) and 647 nm (for Alexa Fluor 647 and Atto 647N), with relevant emission detection bands of 495–525 nm (for Alexa Fluor 488), 540–620 nm (for Atto 532), 560–625 nm (for Quasar 570), 655–725 nm (for Alexa Fluor 647 and Atto 647N). Signals in different channels were detected with photomultipliers (PMT) or Hybrid Detectors (HyD). The typical pixel size is 40 nm. Live-cell imaging of MCF10A cells was performed with a NIKON A1R confocal microscope equipped with a stage top incubator and a hardware autofocus. Images were acquired in 1024×1024 pixel format, with a pixel size of ∼85 nm, without averaging. Images were acquired every 5 min or 10 min over 10–12 h. Excitation was provided with a CW Diode Laser (Coherent, Cube 488-50) at 488 nm for eGFP (DEK–eGFP) and a CW Diode Pumped Solid State Laser (DPSS, Melles Griot, 85YCA) at 561 nm for RFP (pCellCycleChromobody^®^ TagRFP). Widefield images of MCF10A cells were acquired on an AxioObserver or a CellObserver HS microscope (Zeiss) equipped with a PlanApochromat 40×/1.40 oil objective, a HXP 120 mercury arc lamp and an Axiocam MRm CCD camera (1300×1030 pixels). Fluorescence signals were detected with 493/517 nm (GFP and Alexa Fluor 488), 590/612 nm (Alexa Fluor 568) and 640/690 nm (Alexa Fluor 647) filter sets.

For immunofluorescence and nascent DNA staining of U2-OS cells, 5×10^4^–6×10^4^ wild-type or GFP–DEK U2-OS cells were incubated in 12-well plates containing coverslips for 24–36 h at 37°C, 5% CO_2_. If replication foci were visualized, cells were treated prior to fixation directly in the incubator with a 20× working solution of EdU (200 µM; final concentration 10 µM) for 10 min. If not stated otherwise, the following steps were carried out at RT and coverslips were washed at least twice with PBS between each step. For immunofluorescence detection, cells were fixed with 4% paraformaldehyde (PFA) in PBS (20 min). Following, the coverslips were transferred into a staining rack and superfluous PFA was quenched with 50 mM NH_4_C in PBS (10 min). In the case of staining for DEK and PCNA, cells were permeabilized in methanol (5 min, −20°C) instead. Afterwards, cells were incubated in 1% BSA in PBS for 30 min to reduce unspecific binding before incubation with the primary antibody dilution (diluent 1% BSA in PBS) in a dark, humid chamber (overnight, 4°C). After washing with PBS for three times, cells were incubated with the appropriate secondary antibody (diluted in 1% BSA in PBS) in the dark chamber (1 h). If applicable, EdU was detected using the Click-it EdU Imaging Kit with Alexa Fluor 647 azide according to the manufacturer's instructions. Stained coverslips were mounted on microscopy slides using Aqua Polymount and hardened overnight at room temperature or for at least 2 days at 4°C. Coverslips were stored at 4°C. For experiments without simultaneous PCNA chromobody visualization, 7×10^4^ U2-OS GFP–DEK cells were seeded in 8-well ibidi µ-slides 24–36 h prior to imaging and incubated at 37°C under 5% CO_2_. For all time-lapse experiments, standard McCoy's 5a medium was exchanged with imaging medium (Phenol Red-free McCoy's 5a supplemented with 10% FBS, 100 U/ml penicillin and 100 μg/ml streptomycin) prior to imaging. If applicable, imaging medium was also supplemented with the indicated concentration of replication stress drug. If the brightfield channel with differential interference contrast (DIC) slider was used, the standard lid was replaced with a special DIC lid. The cells were then equilibrated in a pre-warmed CellObserver HSl microscope (Zeiss) equipped with a CSU-X1 spinning disc unit (Yokogawa) and a full incubator for 1 h at 37°C, with 5% CO_2_ and a humid atmosphere. Images were acquired in spinning disc mode with a PlanApochromat 20×/0.8 air objective and an Evolve EMCCD camera (Photometrics, 512×512 pixels). For brightfield illumination, a HXP 120 mercury arc lamp was used, for GFP–DEK a 488 nm OPSL laser was used and for TagRFP-chromobody a 561 nm diode laser were used. The laser intensities in the focal plane were adjusted to previously determined values before each experiment with an X-Cite EXFO power meter (488 µW/cm^2^ for 561 nm, 131 µW/cm^2^ for 488 nm), while exposure time and EM gain were set to 150 ms and 700, respectively. For the time-lapse experiment, several imaging positions per well were set, the focus adjusted manually, and images acquired every 10 min for 24–48 h. Cell cycle phase durations of the time lapses were analyzed manually frame by frame using Fiji software.

### 3D structured illumination microscopy

1.3×10^5^ U2-OS wild type or GFP–DEK or 2×10^5^ BJ-5ta cells were incubated in six-well plates containing high precision coverslips (18×18 mm, #1.5) for 18–24 h at 37°C and 5% CO_2_ and processed as described above. If replication foci were visualized, cells were treated prior to fixation with EdU for 10 min as described above. Resulting coverslips were mounted on microscopy slides using 7 µl of Vectashield H-1000, sealed with nail polish and stored at 4°C. Superresolution images were acquired on a DeltaVision OMXv4 Blaze microscope (GE Healthcare) equipped with a PlanApochromat 60×/1.42 oil objective and 4 liquid cooled sCMOS cameras (5.5 megapixels, PCO). For widefield imaging a 6-color LED solid state illuminator and 488/525 nm (GFP, AlexaFluor 488), 568/590 nm (Alexa Fluor 568) and 642/675 nm (Alexa Fluor 647) filter sets were used. For SI imaging 488, 568 and 642 nm diode lasers were used. In a typical experiment, first a stitched low-resolution overview image using the GFP–DEK and the PCNA or the EdU channel was generated in widefield mode. Once a DEK body-positive cell was identified, a *Z*-stack with at least 20 slices (0.125 µm step size) was acquired at maximum resolution in SI mode. Images were reconstructed and registered using softWoRx v6.5.2 (Cytiva). Pseudo-widefield images were generated with Fiji v1.51n (Schindelin et al., 2012) and the SIMcheck plugin v1 (Ball et al., 2015). Line profiles were measured using the ‘Plot Profile’ command in Fiji software, exported as CSV files and visualized with the programming language R (v.3.5.0; https://www.r-project.org/) with the graphical integrated development environment (IDE) RStudio (v1.1.453) and the ggplot2 package (Wickham, 2016).

### RNA-FISH

*Xist* RNA was detected with Stellaris^®^ RNA FISH (Human XIST with Quasar^®^ 570 Dye, Biosearch Technologies, SMF-2038-1) according to the manufacturer's instructions. After the hybridization step, Click-iT EdU detection was performed with the use of Alexa Fluor 647 azide. The samples were washed with PBS and mounted in ProLong™ Diamond Antifade Mountant.

### Proximity ligation assay

Proximity ligation assay (PLA), including controls, was performed in MCF10A cells as described previously (Pierzynska-Mach et al., 2023) and using the DuoLink^®^ *in situ* Orange detection reagent (DUO92102, Sigma-Aldrich) according to the manufacturer's instructions. Cells were incubated for 60 min at 37°C in a humidified chamber in PLA-blocking solution, followed by 1 h at RT with antibody solution containing primary antibodies. After washing, the ligation and amplification steps were performed. Anti-mouse-IgG conjugated to Alexa Fluor 488 was used as secondary antibody. Nascent DNA was detected as described above. Samples were mounted in ProLong™ Diamond Antifade Mountant. Quantitative analysis of ‘PLA-positive’ DEK bodies was carried out by counting cases where the PLA signal was formed in the radius of 1.5 µm from the center of DEK body (Koos et al., 2014).

### STED microscopy

STED measurements were performed on a Leica TCS SP5 gated-STED microscope, using an HCX PL APO 100×100/1.40/0.70 oil immersion objective lens (Leica Microsystems, Mannheim, Germany). Emission depletion was performed with a 592 nm CW STED laser. The 592 nm STED laser power was set at 50% of the 350 mW maximum power (∼175 mW). Excitation was provided with a white laser at 488 nm wavelength for Alexa Fluor 488 and with emission detection band 495–525 nm, with 1.50–9.50 ns time gating using a hybrid detector (Leica Microsystems). Typical pixel size is 40 nm, with the pixel dwell time of 2 µs and 48 lines averaging.

### SMLM imaging

For the super-resolution single-molecule localization microscopy (SMLM) imaging of DEK, MCF10A cells were washed with pre-warmed PBS three times, and fixed with formaldehyde (3.7%, methanol free). After subsequent blocking with blocking buffer [0.1% Triton X-100 and 3% (w/v) of BSA] for 1 h at room temperature, samples were incubated with the primary antibody (overnight at +4°C). F(ab′)2-Goat anti-Mouse IgG (H+L) Cross-Adsorbed Secondary Antibody, Alexa Fluor 647 was used as secondary antibody (Thermo Fisher Scientific, A21237, 1:250, 45 min in RT). Samples were post-fixed in 2% PFA for 5 min and stored in PBS at +4°C until applying GLOX buffer containing a glucose oxidase-based oxygen scavenging system for imaging (Bates et al., 2007). A commercial N-STORM and N-SIM TIRF Eclipse Ti2 microscope equipped with an oil immersion objective (CFI SR HP Apochromat TIRF 100XC Oil, NA 1.49) was used to acquire 20,000 frames at a frame rate of 30 ms using oblique incidence excitation and an sCMOS camera (ORCA-Flash4.0, Hamamatsu Photonics K. K.). Alexa Fluor 647-labeled samples were switched into dark state with the 647 nm laser and reactivated with the 405 nm laser. Imaging cycles consisted of one activation frame followed by three read-out frames. Acquisition was performed using the continuous STORM filter and hardware autofocus. Image reconstruction was performed with the NIS-Elements and N-STORM Analysis (Nikon) imaging software.

### Fluorescence recovery after photobleaching

FRAP measurements were performed on a Leica TCS SP5 confocal laser scanning microscope. The experiments were conducted under a controlled environment in a dedicated live-cell imaging chamber (5% CO_2_, 37°C, 90% humidity). Briefly, regions of interest (ROI)d of 2 μm×2 μm were selected and photobleached with 100% laser power (55 μW at 488 nm). Subsequently, fluorescence recovery was monitored for ∼3 min. ROIs for FRAP were chosen based on the fluorescence intensity of GFP–DEK signal, and spatial characteristics of GFP–DEK foci. GFP–DEK foci were chosen based on their location (close to the nuclear periphery or close to nucleoli). For each FRAP experiment, only a single DEK body was measured per cell. The parameters used for the FRAP measurement are listed in Table S7. For the analysis of FRAP data, first the photobleached region within the nucleus was cropped. After this, the mean intensity of the ROI along the time experiment was plotted. For the FRAP data analysis, we applied the Stowers Institute ImageJ Plugins from Stowers Institute for Medical Research (https://imagej.net/plugins/). Briefly, the FRAP curves were normalized to the minimum and maximum, averaged for subsequent time points of the measurement and the standard deviation was calculated. For calculation of the tau, each fluorescence recovery curve was fit to the exponential model. Tau average and standard deviation values for DEK in chromatin and DEK within DEK bodies were calculated and depicted as a box plot.

### Image analysis and statistical analysis

Image analysis was performed using ImageJ (http://imagej.nih.gov/ij/) and Leica LAS X F (Leica Microsystems GmbH). The number of DEK bodies was obtained from *Z*-stack maximum projections on which the ‘Find Maxima’ function (with the prominence about 80-90 depending on the level of immunofluorescence staining) was performed.

For finding the number of DEK bodies colocalizing with PCNA DNA replication foci, the object-based analysis was performed by using an ImageJ plugin JaCoP (Bolte and Cordelieres, 2006). For the analysis of FRAP data, first the photobleached region within cell nucleus was cropped. After this, the mean intensity of the ROI along the time experiment was plotted, and the curves were combined and normalized to the minimum and maximum of the combined curve. Finally, each of the curves was fit. The tau parameter was obtained from the average of all fits. This procedure was performed for the case of DEK within DEK bodies and for DEK within the other chromatin regions.

Manders' coefficient is a reliable indicator of the proportion of the first channel signal coincident with the signal in the second channel over its total intensity. The Manders’ coefficient varies from 0 to 1, which corresponds to non-overlapping images and 100% colocalization between both images. For analyses the colocalization fraction was calculated using JaCoP Fiji Plugin. This image analysis is based on the Pearson's correlation coefficient. The ‘M1 Manders’ coefficient’ depicted on the graph is defined as the ratio of the summed intensities of pixels from microscopy acquisition channel 1 (in our case, the signal for DEK bodies) for which the intensity in the second channel (histone modifications) is above zero, to the total intensity in the first channel. In other words, it is a reliable indicator of the proportion of the first channel signal coincident with the signal in the second channel over its total intensity. Details of statistical analyses are provided within the figure legends. On overview of sample size and replicates for each experiment can be found in Table S6.

### High-throughput siRNA screen coupled with time-lapse fluorescence microscopy

For preparation of transfection-ready imaging plates, U2-OS GFP–DEK cells were transfected with siRNAs for RNAi according to an established solid-phase lipid-based procedure developed at the EMBL (Neumann et al., 2006). Briefly, imaging plates or microarrays are coated with a mixture of siRNA, medium, sucrose, gelatin and transfection reagent (Lipofectamine 2000, Thermo Fisher Scientific). For the screening assays, 96-well cyclic olefin copolymer (COC) imaging plates (Greiner) and locked nucleic acids (LNA)-modified 21-bp duplex siRNAs with overhang (Silencer Select, Ambion) were used. For the primary screen, the custom-ordered library comprised 678 unique siRNAs and was delivered lyophilized in eight different 96-well plates by the manufacturer (the complete library can be found in Table S1). In each plate, four wells were supplied with sNEG1 negative control, two wells with sPOLD1 (s616) positive control, two wells with sDEK transfection control (s15459) siRNA and three wells were left empty. The rest of the wells were supplied with library siRNAs in an alphabetical order. Before preparing the imaging plates, siRNAs of the mother plate were resolved: 33 µl double distilled (dd)H_2_O was added per well for a final siRNA concentration of 3 µM by an automated multichannel pipette (Integra), incubated for 1 h at RT and mixed eight times to yield a homogenous solution. For the pilot screen, in one 96-well plate, 5 µl of a 3 µM dilution of each control siRNA was distributed into three wells by hand for technical triplicates. Plates were sealed directly after distribution of siRNAs and stored at −20°C until further use. The following protocol is for the coating of eight identical 96-well imaging plates from one unique siRNA layout. All steps were performed at RT. First, the siRNA plate was thawed and 5 µl of the 3 µM siRNA dilution was transferred into a V-shaped 96-well plate using a manual 96-well liquid handling device (Steinbrenner). Then, 11 µl of a mixture of 554 µl of 0.4 M sucrose in Opti-MEM, 323 µl of Lipofectamine 2000 and 323 µl of ddH_2_O were distributed into another V-shaped 96-well plate using a manual eight-channel pipette. The following pipetting steps were performed by an automated multichannel pipette. 7 µl of this transfection reagent mixture was transferred into the previously prepared siRNA plate and mixed eight times. After 20 min of incubation, 7 µl of sterile filtered 0.2% gelatin (0.45 µm pore size) was added to the wells and mixed 8 times. This final transfection mix was diluted 1:51 in water in several steps. in a 96 deep-well plate, 100 µl of ddH_2_O was added per well, then 9 µl of the transfection mix followed by 100 µl and 2×125 µl of ddH_2_O were added. From this diluted transfection mix, 50 µl was distributed into each well of the eight 96-well COC imaging plates. In 100 µl medium, the final siRNA concentration per well was 7.7 nM. Plates were immediately lyophilized (37°C, 1 h) and stored in plastic boxes containing drying pearls (Merck, 94098).

### Cell seeding and time-lapse microscopy

For the first four plates of the primary screen (layout 1–4, replicate 1), 4000 U2-OS GFP–DEK cells per well were seeded in 100 µl McCoy's medium (without antibiotics) into the coated 96-well imaging plate using an automated cell seeding device and incubated for 48 h at 37°C under 5% CO_2_. For all other plates, 3500 cells were seeded because cell confluency was slightly too high in some control wells. At the day of imaging, medium was exchanged with 100 µl pre-warmed Phenol Red-free CO_2_-independent imaging medium (Gibco) supplemented with 10% FBS, 1.5 mM L-glutamine and 2.3 g/l D-glucose. Then the lid was sealed with silicon paste to prevent any gas exchange and the cells were equilibrated in a pre-warmed full incubation IXM XLS high-content widefield microscope (Molecular Devices) for 1 h at 37°C. The microscope was equipped with a PlanApochromat 20×/0.75 air objective and a sCMOS camera (4.66 megapixels). For GFP–DEK detection a 488 nm solid-state light source, the GFP filter set and a 690 nm laser autofocusing system were used, with exposure time was set to 150 ms. For the time-lapse experiment, four imaging positions per well were set and images were acquired every 12 min for 22 h. Two replicate plates per siRNA layout were seeded and imaged, for the three replicates of layout 5. For the pilot screen, 2500–4000 cells per well were seeded. At the day of imaging, 200 nM aphidicolin (Sigma-Aldrich) and or 100 nM camptothecin (Sigma-Aldrich) were added to the imaging medium where indicated and cells were equilibrated in the microscope. For the time-lapse experiment, 4–9 imaging positions per well were set and images acquired every 5–10 min for 24–48 h.

### Image analysis using CellProfiler

The open-source software CellProfiler (v2.1.0; Kamentsky et al., 2011) was used for automated image analysis. The image analysis pipeline encompassed background correction, nuclei segmentation, filtering of dead and mitotic cells, intensity-based detection of DEK bodies and tracking of nuclei via the minimal overlap method.

The following steps were performed sequentially for every image of a time lapse. Images of the same time lapse were correlated using an object (here nuclei) tracking module. Firstly, the Power Log–Log Slope parameter of the raw image was measured for later quality control. Then, the raw image was corrected for uneven illumination due to vignetting and for background fluorescence as we noticed a slightly asymmetric vignetting effect in the raw images, which became more pronounced in later time-lapse images and was intrinsic to the microscopic setup. To compensate, a reference image was taken with the same settings as for the time-lapse experiments, but with a green fluorescent slide in the sample holder instead of an imaging plate. A stack of 20 images at different depths within the several mm thick slide was acquired and a median *Z*-projection generated using Fiji (normalized so that the brightest pixel had an intensity of 1). The raw image was then divided by this reference image to correct the vignetting effect. As the effect was now limited to the corners of the image the top- and left-most 30, the right-most 60 and the bottom-most 160 pixels (px) were cropped (‘cropped image’). Additionally, the autofluorescence of the cyclic olefin copolymer substrate decreased in later time-lapse images due to photobleaching and thusly had to be removed. The cropped image was smoothed by applying a Gaussian filter [full width at half maximum (FWHM) 6 px] and a threshold was calculated automatically with the global Otsu method (min diameter of foreground object 15 px) to identify nuclei. Subsequently, the cropped image was masked with the nuclei outlines and the median intensity of the remaining background signal was subtracted from the cropped image (‘corrected image’). Next, nuclei were segmented. The cropped image was smoothed by applying a Gaussian filter (FWHM 6 px) and the threshold was calculated with the global Otsu method (min diameter of foreground object 35 px). Using this mask on the corrected image, nuclei of dead or mitotic cells were filtered out when meeting following requirements: too high standard deviation and upper quartile of intensity and too small nuclear area. The filtered nuclei were tracked from image to the next image within a time lapse using a simple overlap method with a maximum pixel distance of 15. Afterwards, DEK bodies were identified. The corrected image was smoothed by applying a Gaussian filter (FWHM 4 px) and DEK bodies were enhanced by performing a top-hat morphological operation (diameter 6 px). In rare cases, micronuclei formed at the periphery of the nucleus. To avoid false-positive detection as DEK bodies, these protrusions were removed, and nuclei outlines were smoothed by performing an opening morphological operation (diameter 15 px). DEK bodies were identified within the smoothed outlines using a manual threshold (0.004 arbitrary units) and a diameter of 3–10 px. Automated thresholding was not successful because the signal-to-noise ratio (here between the uniform nuclear GFP–DEK signal and bright DEK bodies) was not large enough. To compare the quality of the image analysis pipeline for different siRNAs and replicates, a compressed overview image was generated displaying DEK bodies encircled in red, DEK body-positive nuclei outlined in red, and the other filtered nuclei outlined in white. Finally, for each image CSV files containing image, nuclei and DEK body measurements were saved before the next image was analyzed. Aggregation of these raw data and evaluation of DEK body measurements was performed with the statistical software package R.

### Data aggregation and transformation using R

The open-source programming language R with RStudio and additional packages were used to aggregate the data. The R code used can be found at https://github.com/CP-Vogel/R-DEK-body-script.git.

The goal was to obtain a single measurement per treatment or siRNA from the complex, time-resolved raw data of the technical (positions) and biological (plates) replicates that could be used to score DEK body regulators. First, the CSV tables from every image of a 96-well plate (one replicate) were concatenated to one single data frame. As CellProfiler tracks the nuclei over subsequent frames/images, these track identifiers were used to summarize relevant measurements, such as length of tracks, length of DEK body sequences, number of DEK bodies per track, nuclear area and intensity, and intensity of DEK bodies etc. We noticed, however, that the CellProfiler pipeline did not always identify DEK bodies in all of the nuclei within a DEK body-containing sequence. We therefore wrote a custom function that used the closing morphological operation to fill these gaps with the DEK body counts of the previous or following frame. In cases where a second, shorter DEK body-containing sequence was detected within the same track, the function also served to remove these likely false-negative DEK bodies by keeping only the longest sequence. Additionally, a similar function was implemented to calculate the mean DEK body count within this longest sequence, which was used as the main readout to score hits. As a consequence of aggregation, for each track only one row was left in the data frame containing the averaged measurements. The data was further summarized over positions/time lapses to investigate the variability within the same siRNAs/treatments and over time points to be able to visualize the time-dependent function of, for example, the number of nuclei or the intensity of nuclear GFP–DEK signal. Afterwards, the aggregated data of all replicates was concatenated. For the primary screen, quality control was then performed based on the position summaries. Positions with a small integrated nuclear fluorescence intensity (very small and/or dark nuclei), very low cell number, extreme values in the Power LogLog Slope (blurry images) and a negative slope of their linear growth function were filtered out. Furthermore, all wells that were contaminated with bacteria or yeast after the time lapse (as seen under a brightfield benchtop microscope) were filtered out manually.

Finally, Z-scores of the position-based summaries of the relevant measurements were calculated for later hit scoring. The *Z*-score can be used when the data distribution is unimodal and variability of the control measurement (here sNEG1) does not change in between replicates (Zhang, 2011). Therefore, the data was log (base 2)-transformed and the mean (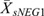) and the standard deviation (s.d._sNEG1_) of all negative control (sNEG1) positions from the same replicate were calculated. For all positions of the other siRNAs of this replicate a Z-score was computed:

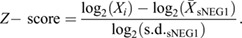
The mean of all Z-scores of the same siRNA was finally calculated. By ordering the mean DEK body count by size it was possible to score DEK body upregulators (highest Z-score) and downregulators (lowest Z-score).

### Low-throughput siRNA validation screen coupled with time-lapse fluorescence microscopy

For siRNA delivery, a liquid-phase instead of a solid-phase reverse transfection procedure was employed. Therefore, transfection mixes had to be prepared freshly for each replicate before cell seeding. Validation siRNAs were picked from the primary screen mother plates into a V-shaped 96-well plate and diluted to 100 nM with MilliQ H_2_O. Two wells were supplied with the same validation siRNA, four wells with sNEG1 negative control, two wells with sDEK transfection control (s15459, Ambion) siRNA and four wells were left empty. The new mother plate was stored at −20°C until the day of transfection. At the day of transfection, 8 µl of a 1:20 dilution of Lipofectamine RNAiMAX in Opti-MEM was added in each well of a 96-well imaging plate (Ibidi) using an eight-channel manual pipette. 25 µl of diluted siRNAs were mixed with the pre-distributed transfection reagent dilution using an eight-channel manual pipette. In two of the four empty wells, no transfection mix was added (no Lipofectamine). After 15 min incubation, 5500 U2-OS GFP–DEK cells in 207 µl medium (no antibiotics) were added for a final siRNA concentration of 10.4 nM and cells were incubated for 48 h at 37°C and 5% CO_2_.

At 48 h after transfection, the medium was exchanged with 200 µl of pre-warmed Phenol Red-free imaging medium (Hyclone, no antibiotics) and equilibrated in a pre-warmed CellObserver HSl microscope (Zeiss) equipped with a CSU-X1 spinning disc unit (Yokogawa) and a full incubator for 1 h at 37°C, 5% CO_2_, humid atmosphere. Images were acquired with a PlanApochromat 20×/0.8 air objective, a 488 nm OPSL laser for GFP–DEK detection and an Evolve EMCCD camera (Photometrics, 512×512 pixels). The laser intensity in the focal plane was increased compared to previous experiments (215 instead of 131 µW/cm^2^) because the imaging interval was also increased (12 min instead of 10 min) and the signal-to-noise ration had to be higher for the automated image analysis pipeline. Exposure time and EM gain were set to 150 ms and 700, respectively. For the time-lapse experiment, six imaging positions per well were set, the focus adjusted manually, and images acquired every 12 min for 24 h. Three replicate plates were acquired in total. In contrast to the primary screen, the image analysis was run on a newer version of CellProfiler (v.2.2.0 instead of 2.1.0) and locally on a workstation instead of a remote cluster. The pipeline was adjusted from the primary screen to meet the needs of the new microscope setup. For background subtraction and nuclei segmentation, the Gaussian filter to smooth the raw image was reduced to a FWHM 6 px and the diameter to segment nuclei via the global Otsu method was adjusted to 23-43 px. Nuclei of dead or mitotic cells were filtered out when meeting following requirements: too high standard deviation of intensity, too low eccentricity, to exclude very round cells, and too high solidity to eliminate ‘ruffled’ shapes that resemble micronuclei. The filtered nuclei were tracked from image to the next image within a time lapse using a simple overlap method with a maximum pixel distance of 15. For DEK body identification, the corrected image was smoothed by applying a 2 px Gaussian filter and DEK bodies were enhanced by performing a 5 px top-hat morphological operation. As micronuclei were eliminated in the previous filtering step, an opening morphological operation did not have to be performed. DEK bodies were identified using a manual threshold of 0.014 AU and a diameter of 2–6 px.

Raw data analysis was performed in a similar manner to that for the primary screen, with the following modifications: the quality control parameters cell number and growth, nuclear fluorescence intensity and Power LogLog Slope were adjusted to meet the differing microscopic parameters (e.g. fewer cells per field-of-view, different signal-to-noise ratio). Additionally, a ratio of the filtered nuclei to all identified nuclei was implemented as a quality control. A time lapse with a low ratio meant many dead or mitotic cells, and was therefore excluded. Calculation of *Z*-scores and siRNA summaries was performed as previously described.

### Preparation of cell extracts, immunoprecipitation and immunoblot analyses

Whole-cell lysates were prepared via scraping cells in PBS containing protease inhibitors (Complete Protease inhibitor cocktail, Roche). Cells were pelleted (300 ***g*** for 5 min at 4°C) and resuspended in hot SDS lysis buffer (0.5% SDS, 50 mM Tris-HCl pH 7.5), incubated for 10 min at 95°C, centrifuged (13,000 ***g*** for 10 min at 4°C), and the resulting samples were frozen in liquid nitrogen and stored at −80°C.

For preparation of cell lysates for immunoprecipitation, cells were harvested in hypotonic buffer [20 mM HEPES pH 7.4, 20 mM NaCl, 5 mM MgCl_2_, 10 mM NaF, 1 mM Na-vanadate, 1 mM PMSF, 1× Complete Protease Inhibitor Cocktail (Roche)] supplemented with 10 mM N-ethylmaleimide (NEM). After centrifugation (5 min at 500 ***g*** and 4°C), the resulting cell pellet was resuspended in hypotonic buffer to 1×10^6^ cells per 100 µl. For extraction of chromatin-bound DEK, 450 mM NaCl and 0.5% NP-40 was added, incubated for 15 min on ice and harvested at 10,000 ***g*** for 10 min at 4°C.

For preparation of cell lysates for nickel affinity chromatography (6×His–SUMO pulldown), a previously published protocol was carried out (Tatham et al., 2009). HeLa cells stably expressing 6×His-SUMO-1 (accession number P62165 in Tatham et al., 2009), 6×His-SUMO-2, (accession number CAG46970 in Tatham et al., 2009) or 6×His-SUMO-3 (accession number CAA67897 in Tatham et al., 2009) as well as HeLa Rachel cells, as negative control, were grown in 10 cm cell culture dishes to 80% confluency. Cells were harvested via scraping in 5 ml PBS containing 10 mM NEM. Crude cell extracts for visualization of SUMO conjugates were prepared with 1 ml of cell suspension. Crude samples were centrifuged (2 min, 1000 ***g*** at RT), the supernatant was removed, and the pellet was resuspended in 200 µl 2× Laemmli sample buffer (200 mM imidazole, 5% SDS, 150 mM Tris-Hcl pH 6.7, 30% glycerol and 0.0025% Bromophenol Blue). Samples were boiled for 5 min at 95°C, then mechanically disrupted by passing through a 20 G needle 20 times using a 1-ml syringe and supplemented with 750 mM β-mercaptoethanol. The remaining 4 ml cell suspension was centrifuged (5 min, 3000 ***g*** at RT) and the pellet was dissolved in 5 ml of cell lysis buffer (6 M Guanidinium-HCl, 10 mM Tris, 100 mM sodium phosphate buffer pH 8.0, 0.1% Triton X-100 and 10 mM NEM). Samples were supplemented with 5 mM β-mercaptoethanol and 5 mM imidazole and sonicated on ice for 40 s at medium power. Cell debris was removed by centrifugation (15 min, 3000 ***g*** at RT) and the supernatant was stored at −20°C.

For immunoprecipitation, all buffers were supplemented with 10 mM NEM. 6 µl of polyclonal rabbit anti-DEK K-877 (Kappes et al., 2008) was used for 100 µl cell lysate. After 30 min incubation on ice, 100 µl of a 25% Protein A/G Plus-Agarose (Santa Cruz Biotechnology) was added and lysate was rotated for 1 h at 4°C. After washing three times with extraction buffer (20 mM HEPES pH 7.4, 300 mM sucrose, 450 mM NaCl and 0.5 mM MgCl_2_), immune complexes were eluted from the beads with 2% SDS and 5% β-mercaptoethanol for 1 h at 37°C. Immunoprecipitation samples were concentrated using methanol-chloroform protein precipitation (Wessel and Flügge, 1984), solubilized in 1× SDS sample buffer and boiled for 10 min at 99°C. Samples were separated by SDS-PAGE, transferred onto nitrocellulose and analyzed by immunoblotting.

For nickel affinity chromatography, all buffers for purification were supplemented with 10 mM NEM of 6×His-SUMO proteins. The prepared cell lysates were subjected to chromatography using HisPur Ni-NTA Resin (Thermo Fisher Scientific). The lysate was added to 100 µl packed volume of beads per sample. Bead slurry was pre-washed three times with ten bead volumes of cell lysis buffer (6 M Guanidinium-HCl, 10 mM Tris, 100 mM sodium phosphate buffer pH 8.0, 0.1% Triton X-100). After tumbling the mixture overnight at 4°C, the bead suspension was centrifuged (2 min, 750 ***g*** at RT) and the supernatant carefully removed. Beads were washed once with 4 ml of cell lysis buffer containing 5 mM β-mercaptoethanol and once with 4 ml of 8.0 pH wash buffer (8 M Urea, 10 mM Tris, 100 mM sodium phosphate buffer pH 8.0, 0.1% Triton X-100 and 5 mM β-mercaptoethanol). Subsequently, the beads were washed three times with 4 ml of 6.3 pH wash buffer (8 M Urea, 10 mM Tris, 100 mM sodium phosphate buffer pH 6.3, 0.1% Triton X-100 and 5 mM β-mercaptoethanol). Finally, the beads were resuspended in 1.5 ml of 6.3 pH wash buffer and then transferred to 1.5 ml centrifuge tubes. The wash buffer was completely removed after centrifugation (1 min, 6000 ***g*** at RT) and elution of 6×His–SUMO proteins was performed with 100 µl of elution buffer (200 mM imidazole, 5% SDS, 150 mM Tris-HCl pH 6.7, 30% glycerol, 720 mM β-mercaptoethanol and 0.0025% Bromophenol Blue) per sample. The beads were boiled for 2 min at 95°C and then incubated for 20 min at RT. The eluted fraction and the crude cell extracts were separated by SDS-PAGE, transferred onto nitrocellulose and analyzed by immunoblotting.

For analysis of whole-cell lysates 40 µg of protein were loaded onto a 12% SDS gel and separated by electrophoresis. Proteins were transferred onto a nitrocellulose membrane using semi-dry western blotting.

For analysis of eluted 6×His-SUMO proteins, 100 µl of the eluted fraction and input (crude) samples were loaded onto a 10% SDS gel and separated by electrophoresis. Proteins were transferred onto a nitrocellulose membrane using wet western blotting.

DEK immunoprecipitation eluates were separated on a 12.5% SDS gel. Membranes were blocked in 5% milk in Tris-buffered saline with 0.1% Tween 20 (TBS-T) and incubated with primary antibodies overnight at 4°C under mild agitation (anti-DEK K-877 antibody). Membranes were washed and incubated with HRP-conjugated secondary antibodies for 1 h at RT (goat anti-rabbit-IgG conjugated to HRP abd goat anti-mouse-IgG conjugated to HRP, both Dako Denmark). Marker lanes were incubated with Streptavidin-biotinylated HRP (GE Healthcare). Proteins were visualized using chemiluminescent detection with ECL Prime western blotting detection reagents (Amersham RPN2232).

For densitometric quantification, obtained immunoblot images were analyzed with Fiji software. Using the rectangular tool, the single lanes were defined manually. Per lane histograms were analyzed and signal intensities of relative bands determined by integration. For 6×His–SUMO pulldown, blots with DEK-specific antibody were used for quantification. Signal intensities of the mono-SUMOylated DEK bands were normalized to the input bands (the input level was set to 100%) and the respective monoSUMOylated DEK signal was calculated as a percentage of input. For quantification of *in vitro* SUMOylation, the blot with DEK-specific antibody was used. First, the ratio between monoSUMOylated DEK and unmodified DEK was calculated. Next, this ratio value of wild-type GST–DEK was set to 1 and the relative GST–DEK SUMOmut signal was calculated accordingly (see below for details of purification of GST-tagged proteins).

### Site-directed mutagenesis

Nucleobase mutations of the DEK sequence were introduced using an improved QuikChange site-directed mutagenesis protocol (Zheng et al., 2004). The pEGFP-N1-hDEK plasmid (DEK WT sequence inserted into eGFP reporter plasmid peGFP-N1; Addgene 6085-1) was used as a template for mutagenesis PCR. The potential SUMOylation site 1 (bases 181–186) and the SUMO consensus motif (bases 778–789) were mutated using overlapping primers containing the desired base change. For the SUMOylation site 1, two codons were mutated (lysine 61 and 62 to alanine) in two rounds of mutagenesis. For the SUMO consensus motif, one round of mutagenesis was performed (lysine 261 to alanine). For SUMO consensus motif mutation, the plasmid DNA was denatured prior to the PCR using 0.2 M NaOH to reduce the number of colonies containing non-mutated wild-type plasmid after transformation. Primer sequences are listed in Table S5.

### Expression and purification of recombinant GST-tagged proteins from *E*. *coli*

The mutated DEK sequences (DEK SUMOmut 61-62, 144, 145 or 261) were inserted into a GST expression plasmid (pGEX 4T-1), transformed into *Escherichia coli* BL21(DE3) (Thermo Fisher Scientific) and purified as described previously (Ganz et al., 2019). Briefly, protein expression was induced with 0.5 mM IPTG (Sigma-Aldrich) for 1.5 h. Bacteria were harvested via centrifugation at 4600 ***g*** for 15 min at 4°C and resuspended in resuspension buffer (20 mM Tris-HCl pH 8.0, 1 M NaCl, 0.5 mM EDTA and 1 mM DTT) and frozen in liquid nitrogen. After thawing, bacteria were sonicated on ice and 0.5% NP-40 (Sigma-Aldrich) was added and centrifuged at 18,000 ***g*** for 30 min at 4°C. The supernatant was added to 200 µl Glutathione-Sepharose 4B-beads (GE Healthcare) and incubated for 2 h at 4°C. After centrifugation, beads were washed with wash buffer with decreasing NaCl concentrations (500 mM, 300 mM and 20 mM) and GST-tagged DEK was eluted with 200 µl elution buffer [200 mM Tris-HCl pH 8.0, 200 mM NaCl, 40 mM reduced glutathione (Sigma-Aldrich) and 10% glycerol] for 1 h at 4°C. The supernatant was frozen in liquid nitrogen and stored at −80°C.

### *In vitro* SUMOylation

For *in vitro* SUMOylation, recombinant SUMO E1, SUMO E2 (UBC9), SUMO-1 and SUMO-2 were purchased from Enzo Life Sciences (Lausen, Switzerland). Reactions were performed for 2 h at 30°C in *in vitro* buffer [20 mM Tris-HCl pH 7.6, 50 mM NaCl, 10 mM MgCl_2_, 0.1 mM dithiothreitol (DTT), 1× protease inhibitor cocktail (cOmplete™, Mini, EDTA-free Protease Inhibitor Cocktail, Roche) with or without 4 mM ATP] as described previously (Aichem et al., 2019). The reaction was terminated by addition of 5× SDS sample buffer with 4% β-mercaptoethanol and boiled for 5 min at 95°C. 20 µl reactions were mixed containing 0.71 µg recombinant protein (GST, GST–DEK, GST–DEK SUMOmut 61-62, GST–DEK SUMOmut 261 or GST–DEK SUMOmut 61,62,144, 145, 261), 450 ng SUMO E1, 1 µg SUMO E2 and 3 µg SUMO-1 or SUMO2.

### Cell fractionation

Cell fractionation of U2-OS cells was performed as described previously (Kappes et al., 2001). U2-OS WT cells were plated 3 days prior to fractionation in two 15 cm-dishes with 8×10^5^ cells. Cells were washed three times with 5 ml ice cold hypotonic buffer [20 mM HEPES pH 7.4, 20 mM NaCl, 5 mM MgCl_2_, 10 mM NaF, 1 mM Na-vanadate, 1 mM PMSF, 1× Complete Protease Inhibitor Cocktail (Roche)], then scraped on ice in 1.5 ml hypotonic buffer per dish and pooled. After centrifugation (5 min, 500 ***g*** at 4°C) the resulting cell pellet was resuspended in hypotonic buffer to 2.5 10^4^ cells/µl, incubated on ice for 10 min and lysed by Dounce homogenization. After 5 min on ice, the cytosolic fraction containing supernatant was separated from the nuclear fraction by centrifugation (5 min, 500 ***g*** at 4°C). The cell pellet was vortexed carefully and resuspended in hypotonic buffer with 0.5% NP-40, incubated on ice for 15 min, and the supernatant containing the nucleosolic fraction was collected by centrifugation (5 min, 1000 ***g*** at 4°C). The cell pellet was vortexed carefully and resuspended sequentially in extraction buffer [20 mM HEPES, pH 7.4, 300 mM Sucrose, 0.5 mM MgCl_2_, 10 mM NaF, 1 mM Na-vanadate, 1 mM PMSF, 1× Complete Protease Inhibitor Cocktail (Roche)] with increasing NaCl concentrations of 100 mM, 250 mM and 500 mM, incubated on ice for 15 min and the corresponding fraction containing structure-bound proteins was collected by centrifugation (5 min, 1000 ***g*** at 4°C). The final pellet was resuspended in RIPA buffer [50 mM Tris-HCl, pH 8.0, 150 mM NaCl, 1% NP-40, 0.5% deoxycholate and 0.1% SDS], incubated on ice for 15 min and centrifuged (10 min, 10,000 ***g*** at 4°C).

### Electrophoretic mobility shift assay

Recombinant purified proteins (GST–DEK wild-type, GST–DEK SUMOmut Δ61,62,144,145,261) were dialyzed against nE100 buffer (20 mM HEPES-KOH, pH 7.6, 100 mM NaCl, 10 mM NaHSO_3_ and 1 mM EDTA) in the presence of 1 µg/ml BSA on Millipore filters (VSWP 0.025 µm) for 90 min at 4°C. 176 ng of plasmid DNA (pPAGFP-H1.2) were incubated with increasing amounts of GST–DEK in a total volume of 30 µl nE100 for 60 min at 37°C. Samples were mixed with 5× loading dye (50% glycerol and 0.4% orange-G in nE100) and loaded onto a 0.6% agarose gel in 1× TBE (50 mM Tris base, 80 mM boric acid and 1 mM EDTA, pH 8.0) and run at 1.75 V/cm for 16 h. The gel was stained using GelRed nuclear acid stain and DNA was visualized by exposure to UV light.

## Supplementary Material

10.1242/joces.261329_sup1 Supplementary information
