# Supplementary material for: DEK oncoprotein participates in heterochromatin replication via SUMO-dependent nuclear bodies
Source: J Cell Sci. 2023 Dec 15;136(23):jcs261329. doi: 10.1242/jcs.261329 (PMC10753498; doi:10.1242/jcs.261329)
Supplement: Supplementary information [file joces-136-261329-s1.pdf]

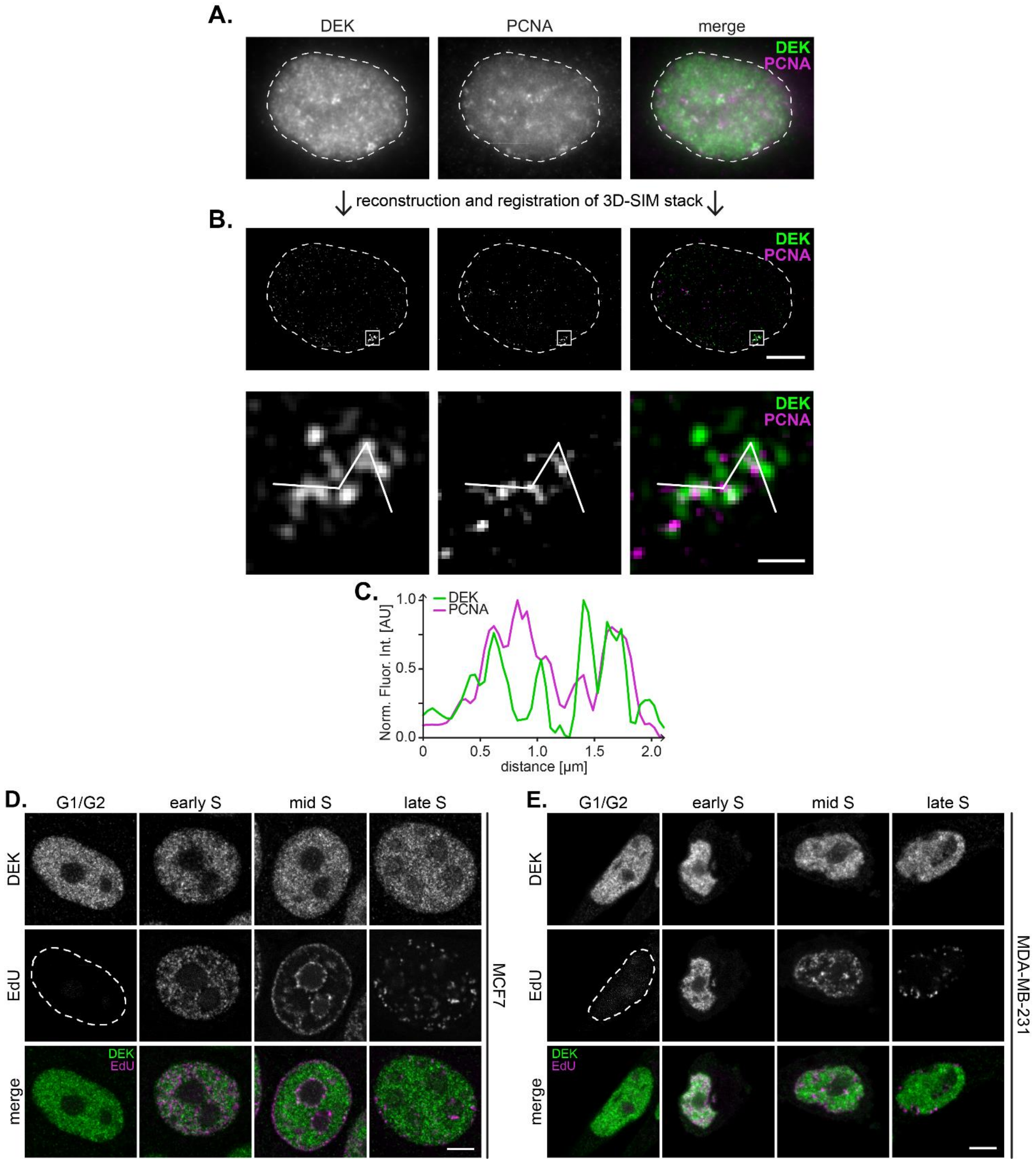

Fig. S1. Endogenous DEK forms DEK foci in primary BJ5-ta cells, yet not in malignant MCF7 and MDA-MB-231 cells.

A. Occurrence of DEK bodies was analyzed via immunofluorescence analysis of DEK (green, DEK 877 Ab) and PCNA (magenta) distribution in a BJ5-TA fibroblast nucleus by super-resolution microscopy (3D-SIM) via indirect immunofluorescence (see Fig. 2C, D and E for details). Inspection of four cells from two biological replicates revealed ~ 2 DEK bodies per cell.

Journal of Cell Science • Supplementary information

B. Image of a single Z-slice from a middle section o f the super-resolved image stack after reconstruction and registration. Scale bar: 5 µm. The magnified inset shows one DEK- positive large replication focus. Scale bar: 0.5 µm.

C. Fluorescence intensity profiles of DEK and PCNA. Interpolated intensity profiles were calculated along the line indicated in (B) and normalized to the min/max values.

D, E No DEK bodies were observed in (D) MCF7 and (E) MDA-MB-231 S-phase cells. Immunofluorescence analysis of DEK (Santa Cruz) and EdU labeling was performed as described in Fig. 1. Shown are representative images for each cell line. Scale bar: 5 µm.

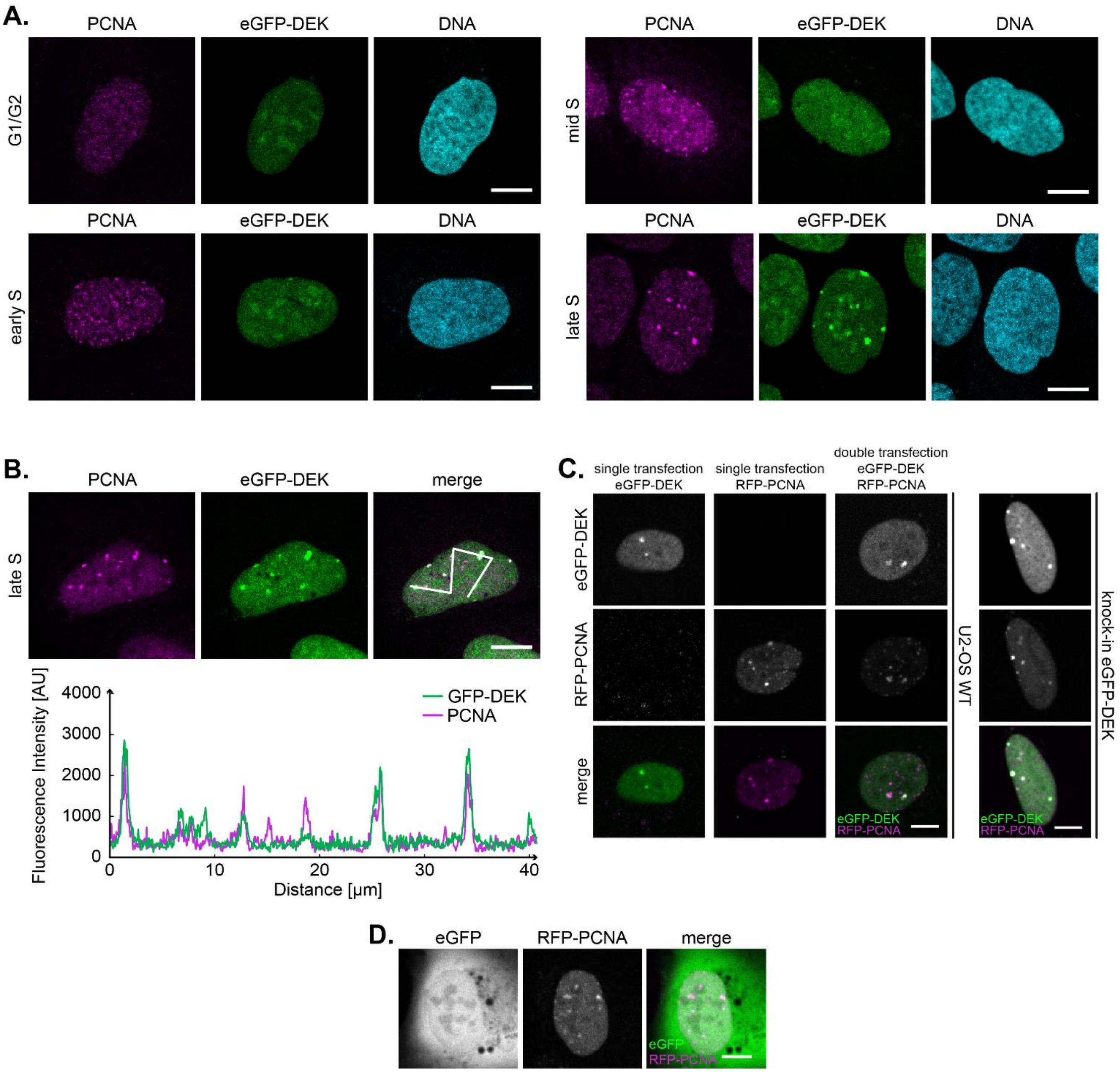

Fig. S2. Colocalization of eGFP-DEK and PCNA during late S- phase in U2-OS cells carrying a TALEN-mediated eGFP-DEK genomic knock in.

A. Confocal images of U2-OS KI eGFP-DEK cells in different cell cycle phases labeled with PCNA-specific antibodies. Similar focal accumulations of eGFP-DEK (green) and PCNA (magenta) can be observed in late S-phase. DNA was counterstained with Hoechst33342 (cyan). Shown is one representative image from 280 inspected cells from two independent experiments inspected. Scale bar: 10 µm.

B. Colocalization analysis of PCNA and DEK bodies in U2-OS KI eGFP-DEK cells. Scale bar: 10 µm. Representative images (out of 33 inspected cells from two independent experiments) are shown, and intensity profile was analyzed along the white line displayed in the right panel.

Journal of Cell Science • Supplementary information

C. DEK body formation is independent of the DEK expression level. U2-OS wild type cells were transfected with plasmids either coding for pEGFP-N1-hDEK or pENmRFP-PCNAL2 or double transfected with both plasmids and incubated for 24 h for maximal protein expression. U2-OS KI eGFP-DEK cells were transfected with pEN-mRFP-PCNAL2 only. Cells were monitored for 24 h (37°C, 5% CO_2). Concomitant with DEK foci formation, relocation of RFP-PCNA (magenta) can be detected both in transiently transfected cells and in the stable cell line. Single transfected cells (upper two panels) serve as control cells. Scale bar: 10 µm.

D. DEK foci formation is not an eGFP artifact. U2-OS cells were double transfected with pEGFP-C1 and pENmRFP-PCNAL2 and incubated for 24 h. During RFP-PCNA body formation, an equally distributed eGFP signal can be visualized. Shown is one representative image from 12 cells from one experiment. Scale bar: 10 µm.

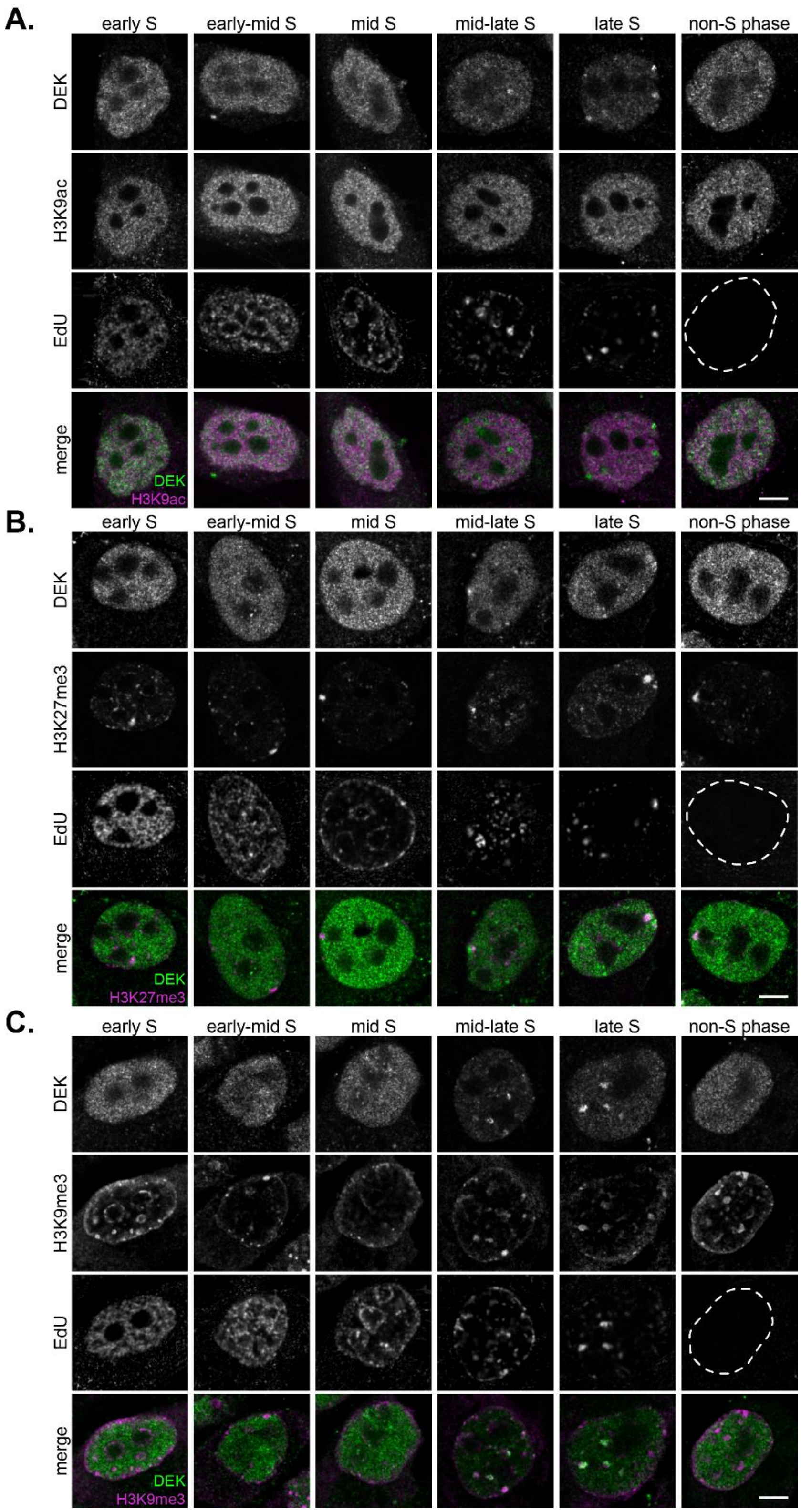

Fig. S3. Localization analysis of DEK and histone marks in S- phase (data set corresponding to Fig. 3A).
confocal images of MCF10A cells labeled with antibodies specific for DEK and histone post-translational modifications H3K9ac (A), H3K27me3 (B), and H3K9me3 (C) during S-phase. Shown are representative images from 95 cells analyzed in five independent experiments. Scale bars = 5 μm.

Journal of Cell Science • Supplementary information

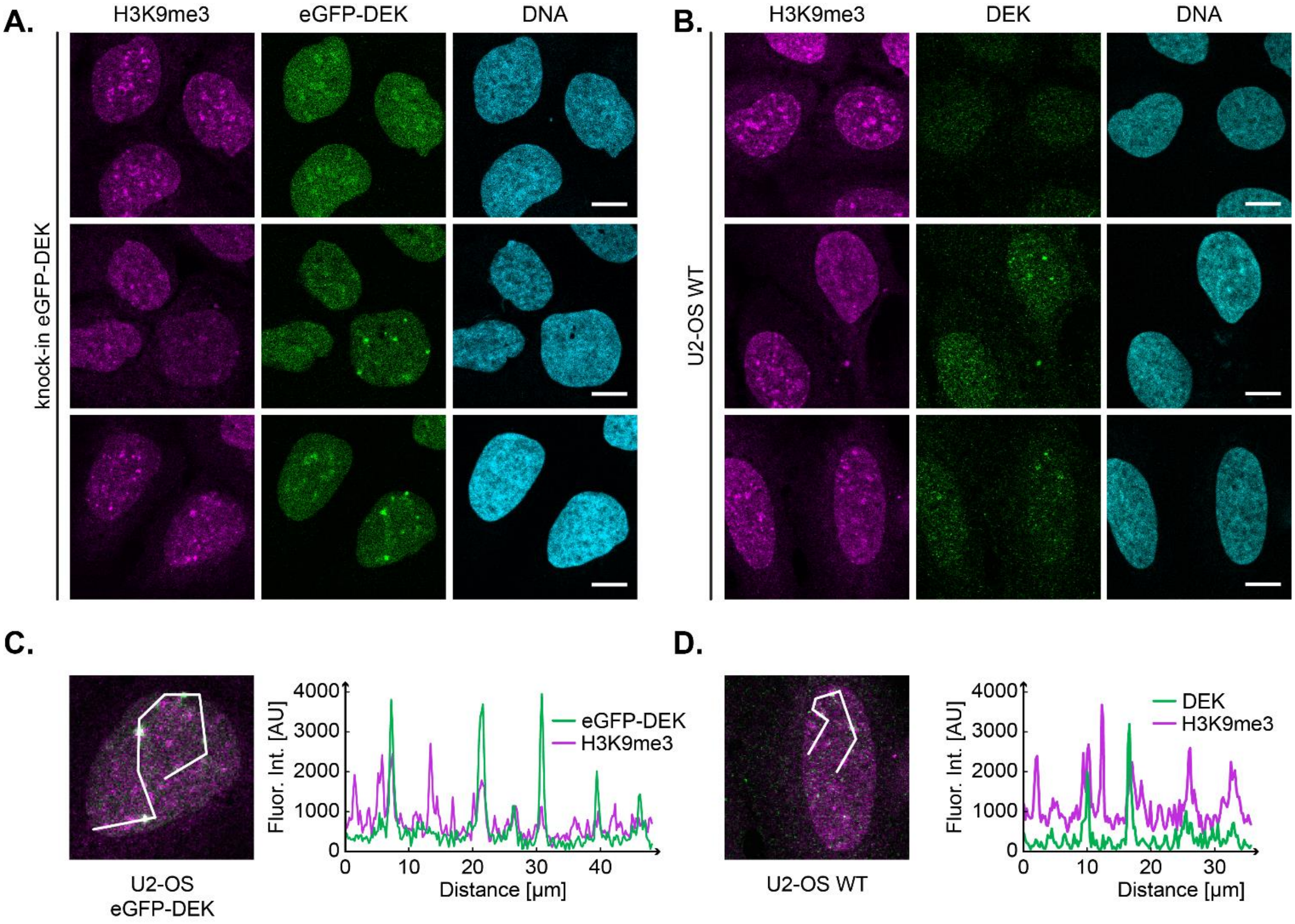

Fig. S4. DEK bodies co-localize with heterochromatin in U2-OS cells.

A, B. Representative confocal images of U2-OS KI eGFP-DEK labelled with H3K9me3 specific antibodies (magenta) (A) and of U2-OS WT cells (B) additionally labelled with antibodies specific for DEK (green). DNA was counterstained using Hochst33342 (cyan) Shown is one representative image from 300 cells from two independent experiments. Scale bar: 10 µm.

C, D. Confocal images of single U2-OS KI eGFP-DEK and U2-OS WT cells from (A) and (B) with the respective fluorescence intensity profiles for the analysis of H3K9me3 (magenta) and DEK (green) colocalization. The white line is given as distance in the graph Shown is one representative image from 150 cells from two independent experiments.

Journal of Cell Science • Supplementary information

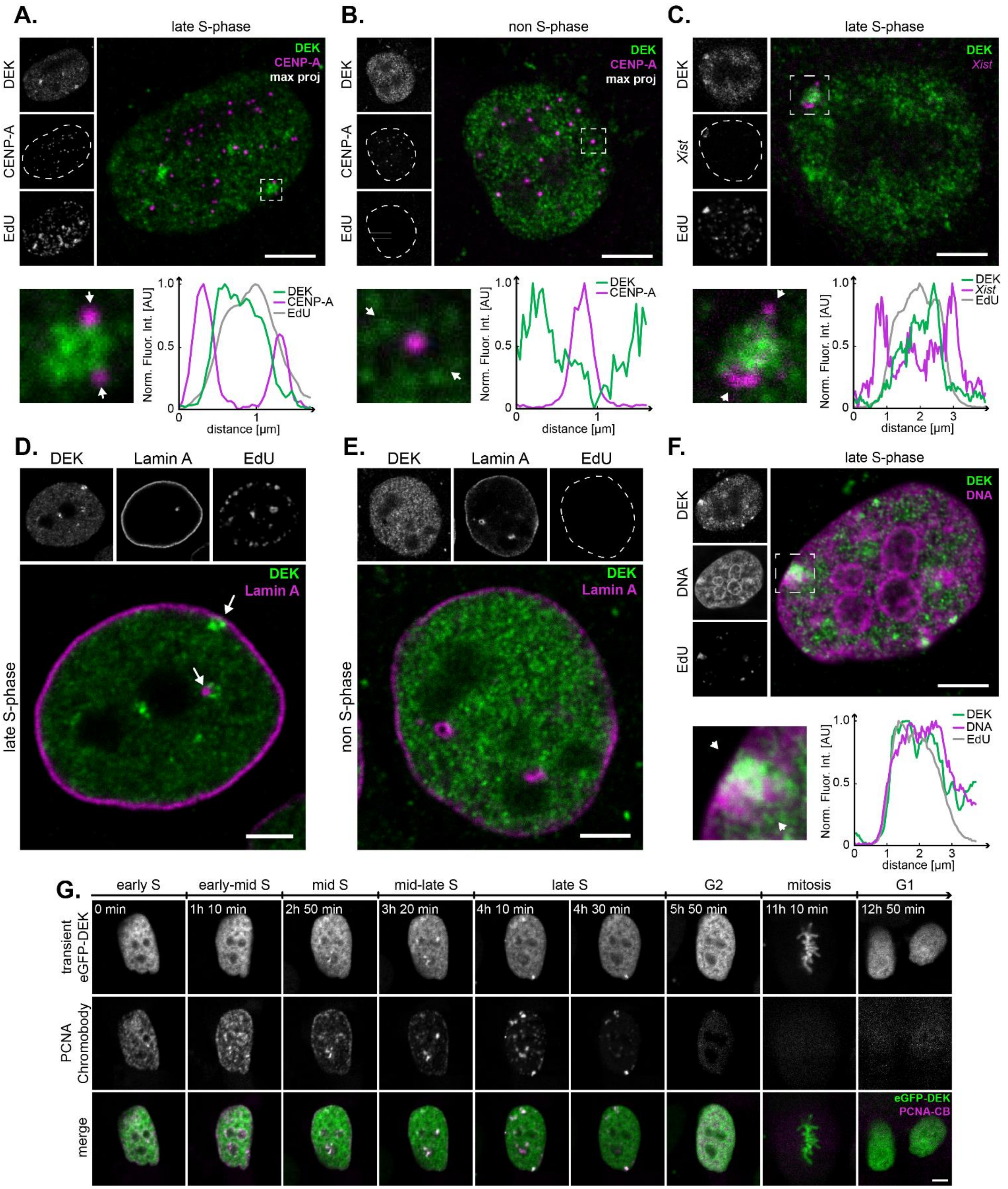

Fig. S5. DEK bodies are spatially associated with replicating heterochromatin. A, B. Maximum intensity projection images of representative MCF10A cell nuclei in late S-phase (A, C), and in the absence of DNA replication (B, non-S-phase, EdU-negative) labelled with anti-DEK and anti-CENP-A antibodies, and EdU-AF467. ROIs are marked by a dashed line and displayed as magnified images below. The fluorescence intensity profiles were

calculated along the lines delimited by the white arrowheads and normalized between 0 and 1. Scale bars: 5 µm.

C. Maximum intensity projection image of a representative MCF10A cell nucleus in late S-phase labelled with anti-DEK antibodies, a *Xist* RNA-specific FISH probe, and EdU-AF467. The ROI marked by a dashed line is displayed as magnified image below. The fluorescence intensity profile was calculated along the lines delimited by the white arrowheads and normalized between 0 and 1. Scale bars: 5 µm.

D, E. Confocal images of representative MCF10A cell nuclei in late S-phase (D) and in the absence of replication (E, non-S-phase, EdU-negative) labelled with anti-DEK and anti-lamin A antibodies, and EdU-AF467. Arrows in (D) indicate the juxtaposition of DEK and lamin A at the periphery of the cell nucleus and at nuclear lamina invaginations, which is not observed in non-S-phase cells (E). Scale bar: 5 µm

F. Confocal image of a representative MCF10A cell nucleus in late S-phase labeled with DEK-specific antibodies, with the DNA dye ToPRo3, and EdU-AF647. The ROI marked by a dashed line is displayed as magnified image below and shows a DEK body overlapping with a region of dense chromatin. The fluorescence intensity profile was calculated along the lines delimited by the white arrowheads and normalized between 0 and 1. Scale bars: 5 µm.

G. Time-lapse fluorescence imaging of MCF10A cells co-transfected with plasmids encoding eGFP-DEK and mRFP-PCNA. The images were acquired at a scanning confocal microscope. Frames were captured every 10 min for 12h. DEK bodies can be traced for about 189 ± 38 min. Analysis of co-localization between DEK bodies and PCNA foci was carried out in 13 cells from 4 independent experiments.

Journal of Cell Science • Supplementary information

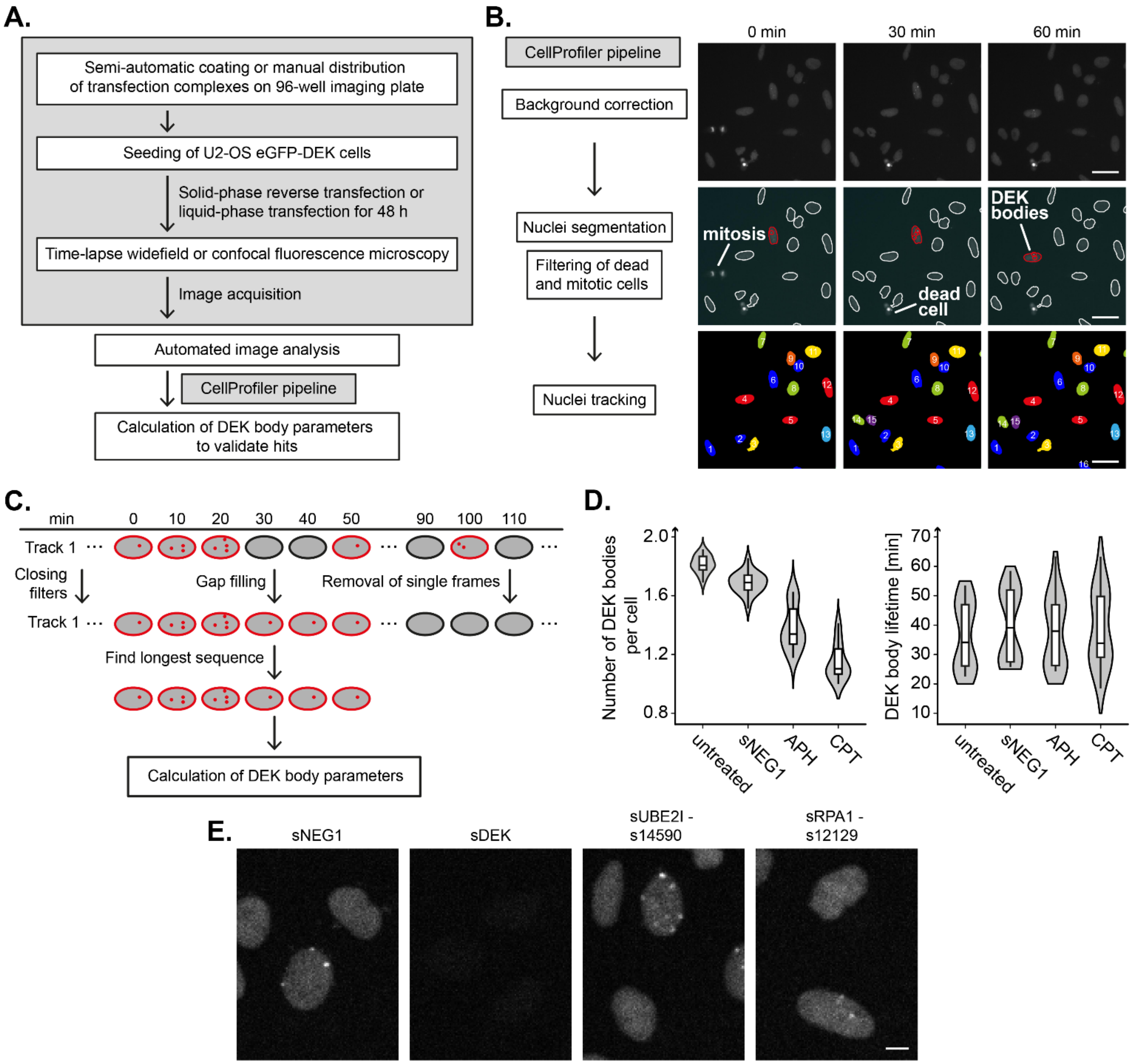

Fig. S6. Design of a high-throughput siRNA screening approach for the identification of DEK body regulators.

A. Schematic of the experimental protocols used for the siRNA screens. The pilot and primary screens relied on automated procedures and solid-phase reverse transfection of the siRNAs. Imaging was performed with a high-content widefield microscope. For the validation screen, cells and transfection reagents were dispensed manually. Imaging was performed on a spinning disk confocal microscope with an automated stage. Two pilot screens with slightly different imaging conditions were performed to optimize imaging parameters. The primary screens were performed in duplicate, and the validation screen in triplicate.

Journal of Cell Science • Supplementary information

B. Main steps of the image analysis pipeline. Example images of cells transfected with a negative control siRNA are shown. Nuclei with detected DEK bodies are outlined in red, nuclei that passed quality control in white. Nuclei that were rejected because of cells were dead or mitotic are not outlined. DEK bodies are encircled in red. Scale bar: 20 µm.

C. Scheme of the gap-filling algorithm used after image analysis. The algorithm removed frames in which detection of DEK bodies within one track failed (false negatives), and sorted out frames in which foci were detected in one frame only (false positives).

D. Quantification of DEK body parameters in image series from one of the pilot screens processed as in (A) and (C). Calculations were done using R. Violin plots display the mean number of DEK bodies per cell (left panel), and the length of the time windows in which DEK bodies appeared (DEK body lifetime, right panel) for the indicated treatments/siRNAs. In total, image data from 12 fields of view (4 positions per well, 3 wells per condition), each containing several tracks, were evaluated. Values from tracks within a field of view were averaged. The violin plot represents the density distribution of these averages, the black line indicates the median, the box the interquartile range (IQR) and the whiskers the IQR ± 1.5.

E. Representative exemplary images of U2-OS KI eGFP-DEK cells treated with sNEG1, sDEK and the DEK body up-regulating siRNA sUBE2I and the down-regulating siRNA sRPA1. Images were taken at a spinning disk confocal microscope. Scale bar: 10 µm.

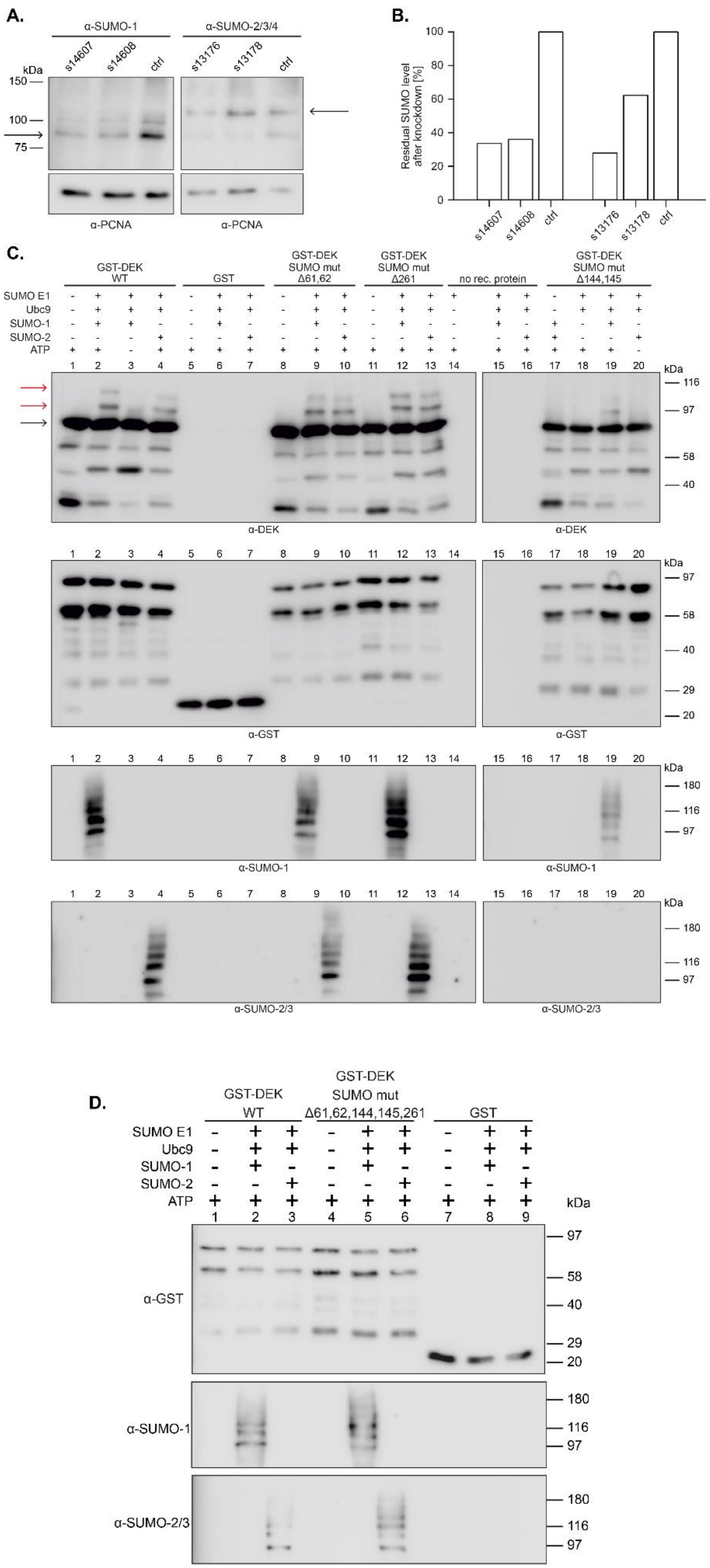

Journal of Cell Science • Supplementary information

Fig. S7. Efficient knockdown of DEK expression after siRNA transfection and unchanged in vitro SUMOylation of DEK single mutants (Control experiments relating to Fig. 6D and Fig. 7D)

A. Immunoblot analysis of SUMO1 and SUMO2/3/4 protein levels after siRNA-mediated knockdown of gene expression. Whole cell lysates from siRNA-transfected U2OS KI eGFP-DEK cells were subjected to Western blotting using SUMO1- and SUMO2/3-specific antibodies, respectively. As negative control, the sNEG1 siRNA was used, PCNA served as loading control. Quantified SUMO signals are marked with an arrow. The experiment was performed once.

B. Densitometric quantification of (A). SUMO-specific signals were normalized to PCNA and are plotted as percentage of their respective controls (PCNA).

C. Samples containing recombinant GST-tagged DEK WT, mutant, or GST only proteins as indicated were subjected to in vitro SUMOylation assays as described in Fig. 7. Proteins were detected with DEK-, GST-, and SUMO-specific antibodies. The black arrow indicates GST-DEK signals, red arrows the mono- and di-SUMOylated variants.

D. Samples containing recombinant GST-tagged DEK WT, mutant, or GST only proteins as indicated were subjected to in vitro SUMOylation assays as described in Fig. 7. Proteins were detected with GST-, and SUMO-specific antibodies. The associated immunoblot with DEK-specific antibodies is shown in Fig. 7D.

Journal of Cell Science • Supplementary information

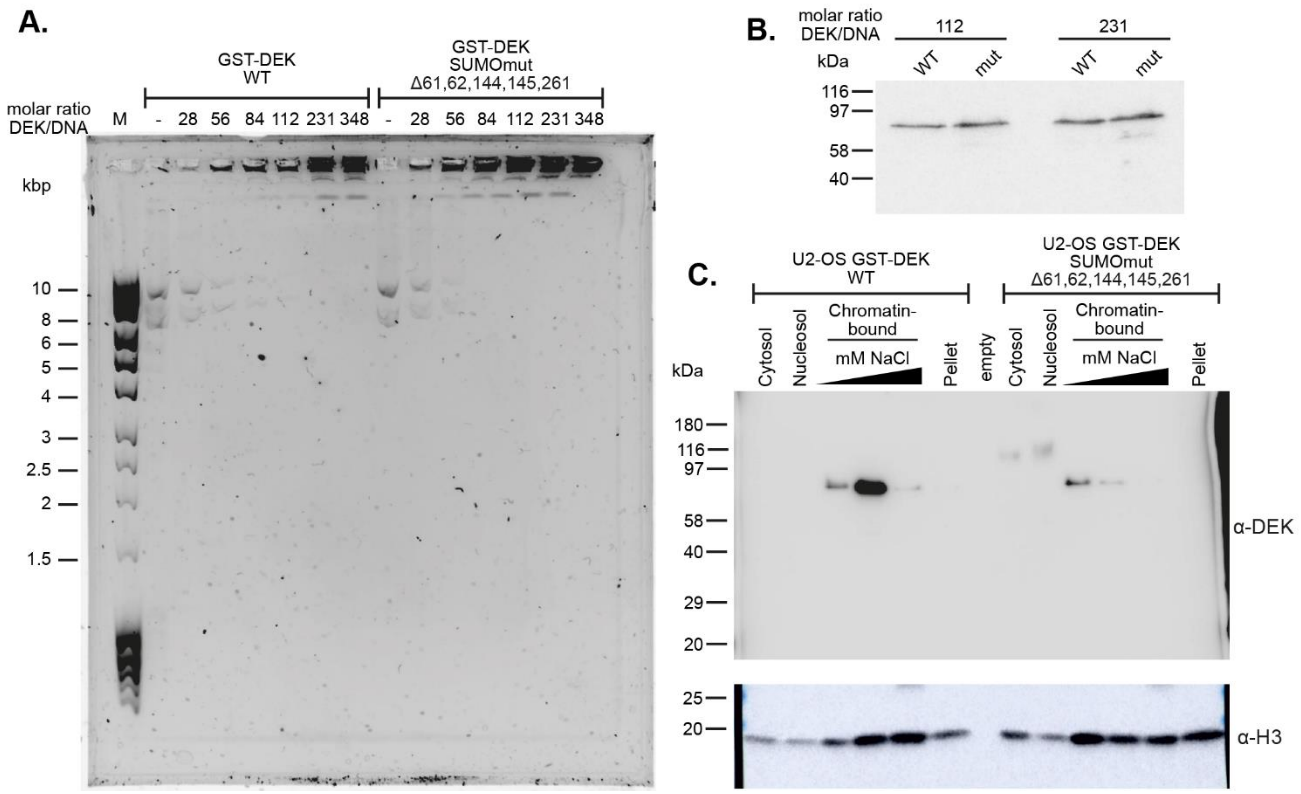

Fig. S8. EMSA and cell fractionation

A. Electrophoretic Mobility Shift Assay (EMSA). Recombinant GST-tagged DEK protein (WT and SUMOmut) were incubated with plasmid DNA in increasing molar ratios as indicated. Agarose gel electrophoresis was performed to separate nucleoprotein complexes. For visualization the gel was incubated in GelRed nucleic acid stain and exposed to UV light. M: Marker.
B. Loading control for EMSA as in A. Equal volumes of recombinant GST-tagged DEK protein dilutions (WT and SUMOmut) used for the molar ratios DEK/DNA 112 and 231 in the EMSA were subjected to SDS-PAGE and immunoblotling. Proteins were detected with DEK-specific polyclonal antibodies (K-877 Ab).
C. Cell fractionation of U2-OS cells expressing GFP-DEK WT and DEK-SUMOmut. The cytosolic fraction was obtained by dounce homogenization of cell suspensions. The nucleosolic fraction was obtained by incubating cells with 0.5 % NP-40. The pellet containing nuclei and chromatin-bound proteins was treated with increasing concentrations of NaCl (100 mM, 250 mM, 450 mM). The remaining pellet was solubilized with RIPA buffer. Equal volumes of cell fractions were subjected to SDS-PAGE and immunoblotting. GFP-DEK was detected with DEK-specific polyclonal antibodies (K-877 Ab) and as a loading control histone H3-specific antibody was used.

Journal of Cell Science • Supplementary information

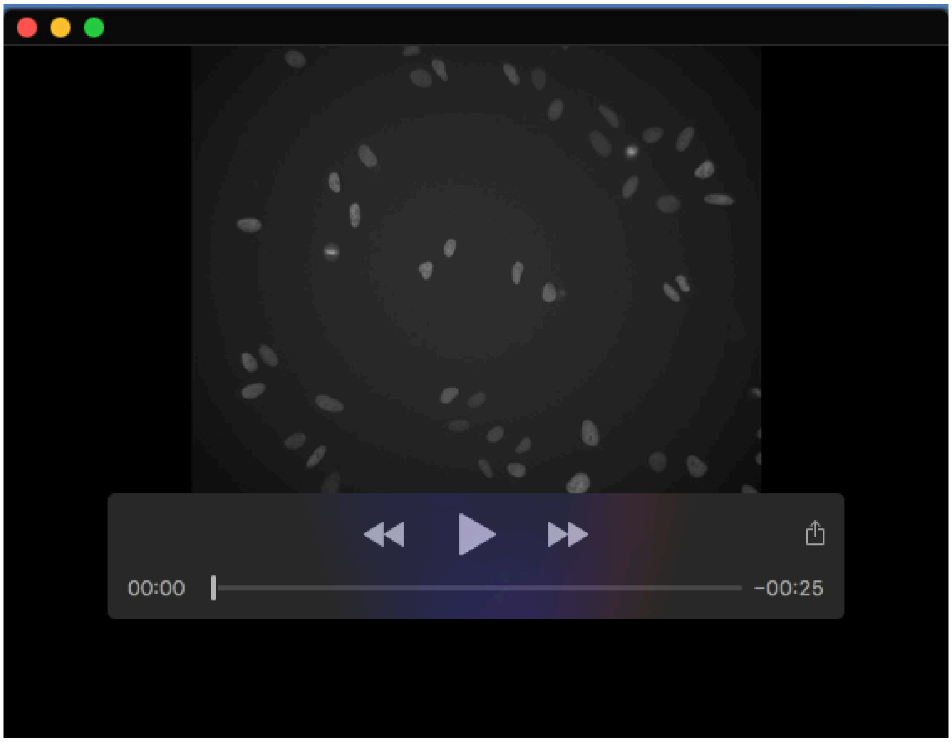

Movie 1. Timelapse widefield microscopy movies of U2-OS cells expressing either eGFP-DEK.

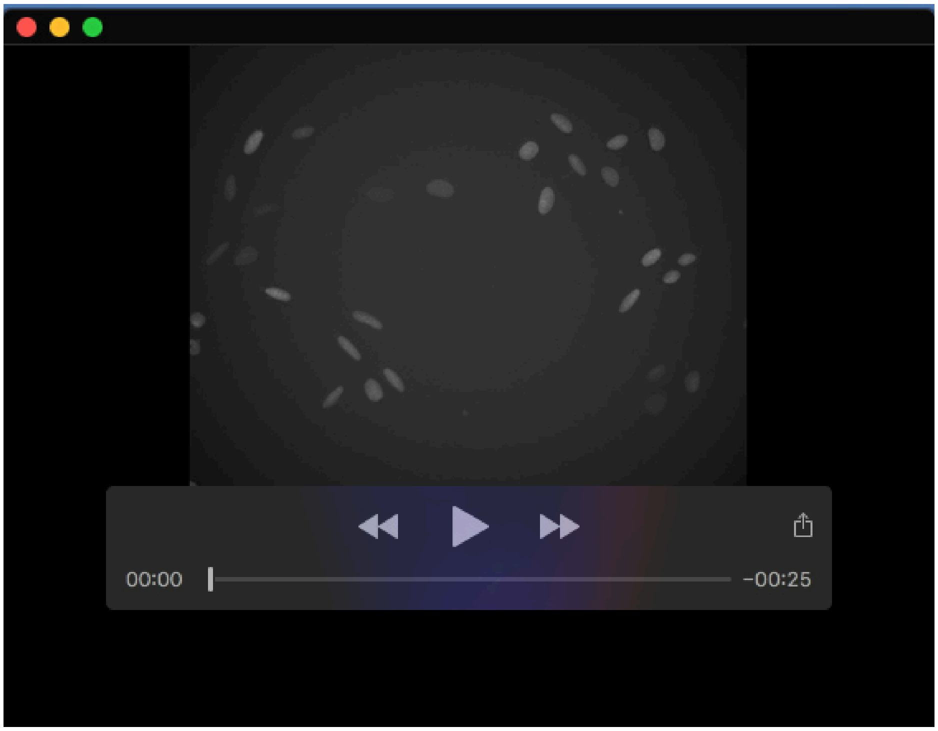

Movie 2. A SUMOylation-deficient DEK mutant - DEKmut (Δ61,62,144,145,261) (movie 2).

Journal of Cell Science • Supplementary information

Table S1. siRNA library of the primary screen. siRNA ID from supplier (Ambion), gene IDs and names from the HUGO Gene Nomenclature Committee (HGNC) as of September 2018. Z-scores of siRNAs which failed quality control are denoted as n.d.

	siRNA ID	Gene ID	Full gene name	Number of DEK bodies (Z-score)		siRNA ID	Gene ID	Full gene name	Number of DEK bodies (Z-score
1	s859	ABCF1	ATP binding cassette subfamily F member 1	2.470	343	s30288	MTPAP	mitochondrial poly(A) polymerase	0.868
2	s860	ABCF1	ATP binding cassette subfamily F member 1	4.913	344	s223607	MTREX	Mtr4 exosome RNA helicase	-1.628
3	s962	ACTL6A	actin like 6A	4.084	345	s23982	MTREX	Mtr4 exosome RNA helicase	0.346
4	s963	ACTL6A	actin like 6A	2.468	346	s9129	MYC	MYC proto-oncogene, bHLH transcription factor	n.d.
5	s1008	ADAR	adenosine deaminase, RNA specific	-1.000	347	s9130	MYC	MYC proto-oncogene, bHLH transcription factor	0.516
6	s1009	ADAR	adenosine deaminase, RNA specific	-0.886	348	s9280	NASP	nuclear autoantigenic sperm protein	-0.984
7	s1445	APEX1	apurinic/apyrimidinic endodeoxyribonuclease 1	1.501	349	s9282	NASP	nuclear autoantigenic sperm protein	0.969
8	s1446	APEX1	apurinic/apyrimidinic endodeoxyribonuclease 1	-1.766	350	s30492	NAT10	N-acetyltransferase 10	0.005
9	s1447	APEX1	apurinic/apyrimidinic endodeoxyribonuclease 1	4.197	351	s30493	NAT10	N-acetyltransferase 10	-0.705
10	s47251	APLF	aprataxin and PNKP like factor	0.472	352	s9291	NBN	nibrin	2.419
11	s47253	APLF	aprataxin and PNKP like factor	2.380	353	s9293	NBN	nibrin	1.101
12	s15785	ARID1A	AT-rich interaction domain 1A	0.610	354	s9312	NCL	nucleolin	2.658
13	s15786	ARID1A	AT-rich interaction domain 1A	0.330	355	s9313	NCL	nucleolin	1.442
14	s31344	ASF1B	anti-silencing function 1B histone chaperone	0.671	356	s22929	NCOA6	nuclear receptor coactivator 6	0.056
15	s31345	ASF1B	anti-silencing function 1B histone chaperone	2.055	357	s22930	NCOA6	nuclear receptor coactivator 6	0.796
16	s31346	ASF1B	anti-silencing function 1B histone chaperone	-0.262	358	s28072	NIP7	NIP7, nucleolar pre-rRNA processing protein	1.214
17	s1709	ATM	ATM serine/threonine kinase	2.185	359	s28074	NIP7	NIP7, nucleolar pre-rRNA processing protein	1.469
18	s57221	ATM	ATM serine/threonine kinase	-0.593	360	s26364	NOB1	NIN1 (RPN12) binding protein 1 homolog	0.136
19	s227305	ATR	ATR serine/threonine kinase	-2.310	361	s26365	NOB1	NIN1 (RPN12) binding protein 1 homolog	0.441
20	s56826	ATR	ATR serine/threonine kinase	4.460	362	s36145	NOL9	nucleolar protein 9	0.893
21	s57368	ATRX	ATRX, chromatin remodeler	0.444	363	s36146	NOL9	nucleolar protein 9	1.602
22	s59082	ATRX	ATRX, chromatin remodeler	1.206	364	s28205	NOP16	NOP16 nucleolar protein	-0.593
23	s22059	BAZ1A	bromodomain adjacent to zinc finger domain 1A	0.311	365	s28206	NOP16	NOP16 nucleolar protein	1.726
24	s22060	BAZ1A	bromodomain adjacent to zinc	1.461	366	s9676	NPM1	nucleophosmin 1	1.069

Journal of Cell Science • Supplementary information

			finger domain 1A						
25	s17209	BAZ1B	bromodomain adjacent to zinc finger domain 1B	-3.348	367	s9677	NPM1	nucleophosmin 1	-0.121
26	s17210	BAZ1B	bromodomain adjacent to zinc finger domain 1B	-1.524	368	s20257	NPM3	nucleophosmin/nucleoplasmin 3	0.157
27	s1997	BLM	BLM RecQ like helicase	-0.470	369	s20258	NPM3	nucleophosmin/nucleoplasmin 3	-0.313
28	s1999	BLM	BLM RecQ like helicase	0.430	370	s29683	NSUN2	NOP2/Sun RNA methyltransferase family member 2	0.057
29	s457	BRCA1	BRCA1, DNA repair associated	2.149	371	s29684	NSUN2	NOP2/Sun RNA methyltransferase family member 2	0.186
30	s458	BRCA1	BRCA1, DNA repair associated	n.d.	372	s9892	ORC1	origin recognition complex subunit 1	-1.750
31	s2083	BRCA2	BRCA2, DNA repair associated	-0.717	373	s9893	ORC1	origin recognition complex subunit 1	-0.101
32	s224695	BRCA2	BRCA2, DNA repair associated	-0.928	374	s9895	ORC2	origin recognition complex subunit 2	0.663
33	s38384	BRIP1	BRCA1 interacting protein C-terminal helicase 1	-1.942	375	s9896	ORC2	origin recognition complex subunit 2	0.279
34	s38385	BRIP1	BRCA1 interacting protein C-terminal helicase 1	1.171	376	s24167	ORC3	origin recognition complex subunit 3	1.647
35	s226839	BRIX1	BRX1, biogenesis of ribosomes	-0.951	377	s24168	ORC3	origin recognition complex subunit 3	-0.264
36	s30678	BRIX1	BRX1, biogenesis of ribosomes	5.168	378	s9898	ORC4	origin recognition complex subunit 4	0.489
37	s2134	BYSL	bystin like	-0.570	379	s9900	ORC4	origin recognition complex subunit 4	0.587
38	s2135	BYSL	bystin like	-0.779	380	s9901	ORC5	origin recognition complex subunit 5	1.883
39	s8377	CAPRIN1	cell cycle associated protein 1	-1.209	381	s9903	ORC5	origin recognition complex subunit 5	0.045
40	s8378	CAPRIN1	cell cycle associated protein 1	1.958	382	s24164	ORC6	origin recognition complex subunit 6	-0.366
41	s21550	CBX1	chromobox 1	1.224	383	s24165	ORC6	origin recognition complex subunit 6	0.163
42	s21551	CBX1	chromobox 1	0.003	384	s9978	PA2G4	proliferation-associated 2G4	3.354
43	s223227	CBX3	chromobox 3	n.d.	385	s9980	PA2G4	proliferation-associated 2G4	0.233
44	s22356	CBX3	chromobox 3	n.d.	386	s25664	PABPC1	poly(A) binding protein cytoplasmic 1	2.385
45	s23883	CBX5	chromobox 5	0.400	387	s25665	PABPC1	poly(A) binding protein cytoplasmic 1	1.163
46	s23884	CBX5	chromobox 5	0.379	388	s25666	PABPC1	poly(A) binding protein cytoplasmic 1	0.496
47	s225505	CCDC124	coiled-coil domain containing 124	-0.784	389	s16693	PABPC4	poly(A) binding protein cytoplasmic 4	0.393
48	s41755	CCDC124	coiled-coil domain containing 124	1.656	390	s52787	PABPC4	poly(A) binding protein cytoplasmic 4	1.145
49	s229	CCND1	cyclin D1	0.168	391	s29268	PAF1	PAF1 homolog, Paf1/RNA polymerase II complex component	0.917
50	s230	CCND1	cyclin D1	1.622	392	s29269	PAF1	PAF1 homolog, Paf1/RNA polymerase II complex component	-0.612
51	s225066	CCNH	cyclin H	1.561	393	s1097	PARP1	poly(ADP-ribose) polymerase 1	3.088
52	s2537	CCNH	cyclin H	1.734	394	s1099	PARP1	poly(ADP-ribose) polymerase 1	0.765
53	s15830	CDC45	cell division cycle 45	n.d.	395	s19502	PARP2	poly(ADP-ribose)	0.489

Journal of Cell Science • Supplementary information

								polymerase 2	
54	s15831	CDC45	cell division cycle 45	-0.062	396	s19504	PARP2	poly(ADP-ribose) polymerase 2	0.528
55	s463	CDK1	cyclin dependent kinase 1	1.842	397	s19505	PARP3	poly(ADP-ribose) polymerase family member 3	3.828
56	s464	CDK1	cyclin dependent kinase 1	3.045	398	s19506	PARP3	poly(ADP-ribose) polymerase family member 3	-0.576
57	s204	CDK2	cyclin dependent kinase 2	0.606	399	s18861	PCLAF	PCNA clamp associated factor	0.983
58	s205	CDK2	cyclin dependent kinase 2	2.967	400	s18863	PCLAF	PCNA clamp associated factor	-1.174
59	s2829	CDK7	cyclin dependent kinase 7	0.353	401	s10134	PCNA	proliferating cell nuclear antigen	0.054
60	s2830	CDK7	cyclin dependent kinase 7	3.290	402	s10135	PCNA	proliferating cell nuclear antigen	0.744
61	s416	CDKN1A	cyclin dependent kinase inhibitor 1A	0.535	403	s25745	PELP1	proline, glutamate and leucine rich protein 1	1.130
62	s417	CDKN1A	cyclin dependent kinase inhibitor 1A	2.379	404	s25746	PELP1	proline, glutamate and leucine rich protein 1	-0.420
63	s2838	CDKN1B	cyclin dependent kinase inhibitor 1B	-4.865	405	s38846	PHF6	PHD finger protein 6	-0.633
64	s2839	CDKN1B	cyclin dependent kinase inhibitor 1B	n.d.	406	s38848	PHF6	PHD finger protein 6	1.556
65	s37723	CDT1	chromatin licensing and DNA replication factor 1	3.911	407	s194691	PML	promyelocytic leukemia	1.334
66	s37724	CDT1	chromatin licensing and DNA replication factor 1	4.045	408	s194692	PML	promyelocytic leukemia	0.540
67	s2909	CENPB	centromere protein B	2.259	409	s22287	PNKP	polynucleotide kinase 3'-phosphatase	1.111
68	s2911	CENPB	centromere protein B	3.030	410	s22288	PNKP	polynucleotide kinase 3'-phosphatase	-0.339
69	s225845	CENPX	centromere protein X	2.345	411	s32352	PNO1	partner of NOB1 homolog	0.338
70	s47345	CENPX	centromere protein X	1.143	412	s32353	PNO1	partner of NOB1 homolog	2.148
71	s19499	CHAF1A	chromatin assembly factor 1 subunit A	-2.102	413	s10772	POLA1	DNA polymerase alpha 1, catalytic subunit	-0.520
72	s19501	CHAF1A	chromatin assembly factor 1 subunit A	-1.132	414	s10774	POLA1	DNA polymerase alpha 1, catalytic subunit	0.928
73	s15705	CHAF1B	chromatin assembly factor 1 subunit B	-2.347	415	s24280	POLA2	DNA polymerase alpha 2, accessory subunit	0.909
74	s15706	CHAF1B	chromatin assembly factor 1 subunit B	1.113	416	s24282	POLA2	DNA polymerase alpha 2, accessory subunit	1.364
75	s503	CHEK1	checkpoint kinase 1	1.706	417	s10775	POLB	DNA polymerase beta	0.987
76	s504	CHEK1	checkpoint kinase 1	2.206	418	s10776	POLB	DNA polymerase beta	0.894
77	s22120	CHEK2	checkpoint kinase 2	-2.010	419	s614	POLD1	DNA polymerase delta 1, catalytic subunit	2.067
78	s22121	CHEK2	checkpoint kinase 2	-0.563	420	s615	POLD1	DNA polymerase delta 1, catalytic subunit	2.261
79	s14676	COL14A1	collagen type XIV alpha 1 chain	0.345	421	s616	POLD1	DNA polymerase delta 1, catalytic subunit	2.145
80	s14677	COL14A1	collagen type XIV alpha 1 chain	0.839	422	s10778	POLD2	DNA polymerase delta 2, accessory subunit	-0.653

Journal of Cell Science • Supplementary information

81	s3495	CREBBP	CREB binding protein	2.794	423	s10779	POLD2	DNA polymerase delta 2, accessory subunit	1.664
82	s3497	CREBBP	CREB binding protein	1.775	424	s21045	POLD3	DNA polymerase delta 3, accessory subunit	0.705
83	s3639	CSNK2A2	casein kinase 2 alpha 2	0.183	425	s21046	POLD3	DNA polymerase delta 3, accessory subunit	1.672
84	s3641	CSNK2A2	casein kinase 2 alpha 2	-0.253	426	s10781	POLE	DNA polymerase epsilon, catalytic subunit	1.945
85	s3642	CSNK2B	casein kinase 2 beta	-0.575	427	s10783	POLE	DNA polymerase epsilon, catalytic subunit	1.647
86	s3643	CSNK2B	casein kinase 2 beta	1.230	428	s10785	POLE2	DNA polymerase epsilon 2, accessory subunit	1.054
87	s3644	CSNK2B	casein kinase 2 beta	-0.870	429	s10786	POLE2	DNA polymerase epsilon 2, accessory subunit	1.264
88	s37000	CTC1	CST telomere replication complex component 1	0.942	430	s10787	POLG	DNA polymerase gamma, catalytic subunit	-1.834
89	s37001	CTC1	CST telomere replication complex component 1	0.916	431	s10788	POLG	DNA polymerase gamma, catalytic subunit	1.189
90	s3935	DAXX	death domain associated protein	2.245	432	s10789	POLG	DNA polymerase gamma, catalytic subunit	1.231
91	s3937	DAXX	death domain associated protein	0.820	433	s22169	POLG2	DNA polymerase gamma 2, accessory subunit	0.378
92	s3979	DDB1	damage specific DNA binding protein 1	0.862	434	s22171	POLG2	DNA polymerase gamma 2, accessory subunit	0.703
93	s3980	DDB1	damage specific DNA binding protein 1	0.143	435	s10790	POLH	DNA polymerase eta	2.702
94	s3982	DDB2	damage specific DNA binding protein 2	1.841	436	s10791	POLH	DNA polymerase eta	-0.207
95	s3984	DDB2	damage specific DNA binding protein 2	1.434	437	s22122	POLI	DNA polymerase iota	-0.082
96	s17564	DDX21	DExD-box helicase 21	0.894	438	s22123	POLI	DNA polymerase iota	-0.357
97	s17565	DDX21	DExD-box helicase 21	-0.037	439	s28115	POLK	DNA polymerase kappa	0.817
98	s32631	DDX24	DEAD-box helicase 24	2.206	440	s28116	POLK	DNA polymerase kappa	1.392
99	s32632	DDX24	DEAD-box helicase 24	1.279	441	s26196	POLL	DNA polymerase lambda	2.335
100	s224194	DDX47	DEAD-box helicase 47	0.065	442	s26197	POLL	DNA polymerase lambda	1.282
101	s27672	DDX47	DEAD-box helicase 47	1.564	443	s51480	POLN	DNA polymerase nu	-1.434
102	s35416	DDX50	DExD-box helicase 50	1.944	444	s51481	POLN	DNA polymerase nu	1.036
103	s35417	DDX50	DExD-box helicase 50	1.305	445	s21059	POLQ	DNA polymerase theta	0.001
104	s21782	DDX52	DExD-box helicase 52	1.162	446	s21060	POLQ	DNA polymerase theta	-0.592
105	s21784	DDX52	DExD-box helicase 52	1.598	447	s719	PPP1CC	protein phosphatase 1 catalytic subunit gamma	1.521
106	s35469	DDX54	DEAD-box helicase 54	2.138	448	s721	PPP1CC	protein phosphatase 1 catalytic subunit gamma	1.275
107	s35470	DDX54	DEAD-box helicase 54	1.000	449	s17267	PRC1	protein regulator of cytokinesis 1	1.026
108	s33616	DDX55	DEAD-box helicase 55	-0.122	450	s17268	PRC1	protein regulator of cytokinesis 1	0.152

Journal of Cell Science • Supplementary information

109	s33617	DDX55	DEAD-box helicase 55	2.435
110	s15457	DEK	DEK proto-oncogene	n.d.
111	s15458	DEK	DEK proto-oncogene	n.d.
112	s15459	DEK	DEK proto-oncogene	n.d.
113	s22643	DHX30	DExH-box helicase 30	n.d.
114	s22645	DHX30	DExH-box helicase 30	0.618
115	s33511	DHX37	DEAH-box helicase 37	2.092
116	s33512	DHX37	DEAH-box helicase 37	1.512
117	s21855	DIDO1	death inducer-obliterator 1	0.749
118	s225469	DIDO1	death inducer-obliterator 1	1.634
119	s4173	DNA2	DNA replication helicase/nuclease 2	0.871
120	s4175	DNA2	DNA replication helicase/nuclease 2	3.145
121	s4215	DNMT1	DNA methyltransferase 1	0.248
122	s4216	DNMT1	DNA methyltransferase 1	1.470
123	s226739	DTL	denticleless E3 ubiquitin protein ligase homolog	4.622
124	s28247	DTL	denticleless E3 ubiquitin protein ligase homolog	-0.331
125	s4390	DUT	deoxyuridine triphosphatase	-0.265
126	s4391	DUT	deoxyuridine triphosphatase	0.933
127	s44664	E2F7	E2F transcription factor 7	3.107
128	s44665	E2F7	E2F transcription factor 7	3.326
129	s21591	EBNA1BP2	EBNA1 binding protein 2	1.863
130	s21592	EBNA1BP2	EBNA1 binding protein 2	2.437
131	s4556	EIF2S1	eukaryotic translation initiation factor 2 subunit alpha	2.458
132	s4557	EIF2S1	eukaryotic translation initiation factor 2 subunit alpha	1.226
133	s17003	EIF2S2	eukaryotic translation initiation factor 2 subunit beta	2.380
451	s11050	PRIM1	DNA primase subunit 1	2.934
452	s11051	PRIM1	DNA primase subunit 1	2.008
453	s11053	PRIM2	DNA primase subunit 2	1.581
454	s11054	PRIM2	DNA primase subunit 2	0.689
455	s61393	PRKDC	protein kinase, DNA-activated, catalytic subunit	2.680
456	s775	PRKDC	protein kinase, DNA-activated, catalytic subunit	0.802
457	s16335	PRKRA	protein activator of interferon induced protein kinase EIF2AK2	0.689
458	s16336	PRKRA	protein activator of interferon induced protein kinase EIF2AK2	1.642
459	s22035	PSIP1	PC4 and SFRS1 interacting protein 1	-2.151
460	s22036	PSIP1	PC4 and SFRS1 interacting protein 1	-2.313
461	s19272	PUM3	pumilio RNA binding family member 3	3.313
462	s19274	PUM3	pumilio RNA binding family member 3	1.864
463	s38873	PYM1	PYM homolog 1, exon junction complex associated factor	4.175
464	s38874	PYM1	PYM homolog 1, exon junction complex associated factor	3.563
465	s38875	PYM1	PYM homolog 1, exon junction complex associated factor	1.821
466	s20341	RACK1	receptor for activated C kinase 1	1.763
467	s20342	RACK1	receptor for activated C kinase 1	-0.552
468	s11589	RAD1	RAD1 checkpoint DNA exonuclease	-0.656
469	s11590	RAD1	RAD1 checkpoint DNA exonuclease	1.099
470	s11723	RAD17	RAD17 checkpoint clamp loader component	n.d.
471	s11724	RAD17	RAD17 checkpoint clamp loader component	2.541
472	s32295	RAD18	RAD18, E3 ubiquitin protein ligase	-0.541
473	s32296	RAD18	RAD18, E3 ubiquitin protein ligase	1.083
474	s11725	RAD21	RAD21 cohesin complex component	-1.311
475	s11727	RAD21	RAD21 cohesin complex component	-1.259

134	s17004	EIF2S2	eukaryotic translation initiation factor 2 subunit beta	1.679	476	s55074	RAD21L1	RAD21 cohesin complex component like 1	0.714
135	s16490	EIF3A	eukaryotic translation initiation factor 3 subunit A	-0.840	477	s55075	RAD21L1	RAD21 cohesin complex component like 1	2.550
136	s16491	EIF3A	eukaryotic translation initiation factor 3 subunit A	-2.310	478	s791	RAD50	RAD50 double strand break repair protein	-0.744
137	s7586	EIF6	eukaryotic translation initiation factor 6	0.853	479	s793	RAD50	RAD50 double strand break repair protein	-0.980
138	s7588	EIF6	eukaryotic translation initiation factor 6	-0.116	480	s11734	RAD51	RAD51 recombinase	-1.159
139	s20415	EMG1	EMG1, N1-specific pseudouridine methyltransferase	1.515	481	s11736	RAD51	RAD51 recombinase	-0.109
140	s20416	EMG1	EMG1, N1-specific pseudouridine methyltransferase	1.682	482	s11746	RAD52	RAD52 homolog, DNA repair protein	0.234
141	s4695	EP300	E1A binding protein p300	0.247	483	s11747	RAD52	RAD52 homolog, DNA repair protein	-0.387
142	s4696	EP300	E1A binding protein p300	1.762	484	s11719	RAD9A	RAD9 checkpoint clamp component A	1.466
143	s4785	ERCC1	ERCC excision repair 1, endonuclease non-catalytic subunit	0.554	485	s11720	RAD9A	RAD9 checkpoint clamp component A	-1.155
144	s4786	ERCC1	ERCC excision repair 1, endonuclease non-catalytic subunit	1.533	486	s224514	RAD9A	RAD9 checkpoint clamp component A	-1.619
145	s230766	ERCC2	ERCC excision repair 2, TFIIH core complex helicase subunit	1.234	487	s44687	RAD9B	RAD9 checkpoint clamp component B	0.640
146	s4787	ERCC2	ERCC excision repair 2, TFIIH core complex helicase subunit	0.212	488	s44688	RAD9B	RAD9 checkpoint clamp component B	n.d.
147	s4796	ERCC3	ERCC excision repair 3, TFIIH core complex helicase subunit	0.368	489	s11837	RBBP4	RB binding protein 4, chromatin remodeling factor	-0.494
148	s4798	ERCC3	ERCC excision repair 3, TFIIH core complex helicase subunit	-0.646	490	s55169	RBBP4	RB binding protein 4, chromatin remodeling factor	2.246
149	s4799	ERCC4	ERCC excision repair 4, endonuclease catalytic subunit	1.444	491	s19398	REC8	REC8 meiotic recombination protein	1.689
150	s4801	ERCC4	ERCC excision repair 4, endonuclease catalytic subunit	1.352	492	s19400	REC8	REC8 meiotic recombination protein	0.672
151	s4805	ERCC6	ERCC excision repair 6, chromatin remodeling factor	3.600	493	s11902	RECQL	RecQ like helicase	-1.321
152	s4806	ERCC6	ERCC excision repair 6, chromatin remodeling factor	1.903	494	s11904	RECQL	RecQ like helicase	-1.414
153	s3104	ERCC8	ERCC excision repair 8, CSA ubiquitin ligase complex subunit	1.704	495	s17989	RECQL5	RecQ like helicase 5	-0.056
154	s3105	ERCC8	ERCC excision repair 8, CSA ubiquitin ligase complex subunit	2.520	496	s17990	RECQL5	RecQ like helicase 5	-1.172
155	s10738	EXOSC10	exosome component	n.d.	497	s28165	REV1	REV1, DNA directed	1.624

			10	
156	s10739	EXOSC10	exosome component 10	3.132
157	s23751	EXOSC2	exosome component 2	1.068
158	s23752	EXOSC2	exosome component 2	2.017
159	s223551	EXOSC7	exosome component 7	3.020
160	s22837	EXOSC7	exosome component 7	1.959
161	s22838	EXOSC7	exosome component 7	0.379
162	s4913	EZH1	enhancer of zeste 1 polycomb repressive complex 2 subunit	0.979
163	s4914	EZH1	enhancer of zeste 1 polycomb repressive complex 2 subunit	0.636
164	s4916	EZH2	enhancer of zeste 2 polycomb repressive complex 2 subunit	0.871
165	s4918	EZH2	enhancer of zeste 2 polycomb repressive complex 2 subunit	0.730
166	s163	FANCA	FA complementation group A	2.179
167	s164	FANCA	FA complementation group A	2.066
168	s4985	FANCC	FA complementation group C	-0.891
169	s4987	FANCC	FA complementation group C	-0.060
170	s4988	FANCD2	FA complementation group D2	-0.923
171	s4989	FANCD2	FA complementation group D2	1.847
172	s4991	FANCE	FA complementation group E	-1.121
173	s4992	FANCE	FA complementation group E	0.808
174	s5015	FANCF	FA complementation group F	-1.387
175	s5016	FANCF	FA complementation group F	0.827
176	s5019	FANCG	FA complementation group G	2.938
177	s5020	FANCG	FA complementation group G	n.d.
178	s33619	FANCM	FA complementation group M	-0.236
179	s33620	FANCM	FA complementation group M	1.203
180	s33621	FANCM	FA complementation group M	-1.705
181	s5039	FAU	FAU, ubiquitin like and ribosomal protein S30 fusion	0.328
182	s5040	FAU	FAU, ubiquitin like and ribosomal protein S30 fusion	0.103
183	s39592	FBH1	F-box DNA helicase 1	1.362
184	s39593	FBH1	F-box DNA helicase 1	0.926
185	s30663	FBXW7	F-box and WD repeat domain containing 7	1.242
			polymerase	
498	s28167	REV1	REV1, DNA directed polymerase	1.314
499	s11939	REV3L	REV3 like, DNA directed polymerase zeta catalytic subunit	0.483
500	s11940	REV3L	REV3 like, DNA directed polymerase zeta catalytic subunit	0.187
501	s11941	RFC1	replication factor C subunit 1	-1.532
502	s224529	RFC1	replication factor C subunit 1	1.643
503	s11945	RFC2	replication factor C subunit 2	-1.078
504	s11946	RFC2	replication factor C subunit 2	0.774
505	s11947	RFC3	replication factor C subunit 3	0.563
506	s11949	RFC3	replication factor C subunit 3	-1.538
507	s11951	RFC4	replication factor C subunit 4	1.250
508	s11952	RFC4	replication factor C subunit 4	2.142
509	s11953	RFC5	replication factor C subunit 5	0.583
510	s11955	RFC5	replication factor C subunit 5	1.186
511	s39523	RIOX2	ribosomal oxygenase 2	2.464
512	s39524	RIOX2	ribosomal oxygenase 2	-0.379
513	s20657	RNASEH2A	ribonuclease H2 subunit A	2.041
514	s20658	RNASEH2A	ribonuclease H2 subunit A	0.785
515	s12074	RNF4	ring finger protein 4	0.128
516	s12075	RNF4	ring finger protein 4	-2.254
517	s12128	RPA1	replication protein A1	-2.745
518	s12129	RPA1	replication protein A1	-2.799
519	s12131	RPA2	replication protein A2	-1.222
520	s12132	RPA2	replication protein A2	-0.313
521	s26742	RPA4	replication protein A4	3.324
522	s26744	RPA4	replication protein A4	-1.724
523	s38769	RPAIN	RPA interacting protein	1.858
524	s38771	RPAIN	RPA interacting protein	0.048
525	s12357	RRM1	ribonucleotide reductase catalytic subunit M1	-1.148
526	s12359	RRM1	ribonucleotide reductase catalytic subunit M1	n.d.
527	s12360	RRM2	ribonucleotide reductase regulatory subunit M2	-0.383

Journal of Cell Science • Supplementary information

186	s30665	FBXW7	F-box and WD repeat domain containing 7	-0.026
187	s5103	FEN1	flap structure-specific endonuclease 1	1.488
188	s5104	FEN1	flap structure-specific endonuclease 1	3.462
189	s42145	FTSJ3	FtsJ RNA methyltransferase homolog 3	-0.125
190	s42147	FTSJ3	FtsJ RNA methyltransferase homolog 3	4.475
191	s19754	G3BP1	G3BP stress granule assembly factor 1	2.027
192	s19756	G3BP1	G3BP stress granule assembly factor 1	1.109
193	s19206	G3BP2	G3BP stress granule assembly factor 2	0.846
194	s19207	G3BP2	G3BP stress granule assembly factor 2	2.606
195	s19030	GINS1	GINS complex subunit 1	0.416
196	s19031	GINS1	GINS complex subunit 1	2.527
197	s28485	GINS2	GINS complex subunit 2	5.608
198	s28486	GINS2	GINS complex subunit 2	-1.297
199	s34942	GINS3	GINS complex subunit 3	n.d.
200	s34943	GINS3	GINS complex subunit 3	0.436
201	s38849	GINS4	GINS complex subunit 4	2.247
202	s38850	GINS4	GINS complex subunit 4	0.272
203	s26649	GNL2	G protein nucleolar 2	4.426
204	s26650	GNL2	G protein nucleolar 2	2.809
205	s25421	GNL3	G protein nucleolar 3	2.904
206	s25422	GNL3	G protein nucleolar 3	1.668
207	s29189	GNL3L	G protein nucleolar 3 like	4.144
208	s29190	GNL3L	G protein nucleolar 3 like	2.640
209	s38171	GRWD1	glutamate rich WD repeat containing 1	0.988
210	s38172	GRWD1	glutamate rich WD repeat containing 1	-0.500
211	s24099	GTPBP4	GTP binding protein 4	1.051
528	s12362	RRM2	ribonucleotide reductase regulatory subunit M2	-0.217
529	s26992	RRM2B	ribonucleotide reductase regulatory TP53 inducible subunit M2B	0.688
530	s26993	RRM2B	ribonucleotide reductase regulatory TP53 inducible subunit M2B	0.462
531	s25187	RSL1D1	ribosomal L1 domain containing 1	1.143
532	s25189	RSL1D1	ribosomal L1 domain containing 1	3.128
533	s24739	RTTN	rotatin	1.001
534	s24741	RTTN	rotatin	2.787
535	s19550	SAE1	SUMO1 activating enzyme subunit 1	3.740
536	s19552	SAE1	SUMO1 activating enzyme subunit 1	0.228
537	s25208	SENP3	SUMO specific peptidase 3	-0.317
538	s25209	SENP3	SUMO specific peptidase 3	1.509
539	s25142	SERBP1	SERPINE1 mRNA binding protein 1	2.457
540	s25144	SERBP1	SERPINE1 mRNA binding protein 1	-0.303
541	s12705	SET	SET nuclear proto-oncogene	1.325
542	s12706	SET	SET nuclear proto-oncogene	1.824
543	s19110	SETDB1	SET domain bifurcated 1	0.890
544	s19112	SETDB1	SET domain bifurcated 1	1.919
545	s38215	SETDB2	SET domain bifurcated 2	0.172
546	s38217	SETDB2	SET domain bifurcated 2	2.643
547	s12708	SETMAR	SET domain and mariner transposase fusion gene	0.179
548	s12709	SETMAR	SET domain and mariner transposase fusion gene	-0.024
549	s23770	SIRT1	sirtuin 1	0.412
550	s23771	SIRT1	sirtuin 1	-0.217
551	s39053	SLX4	SLX4 structure-specific endonuclease subunit	-0.098
552	s39054	SLX4	SLX4 structure-specific endonuclease subunit	2.100
553	s13131	SMARCA1	SWI/SNF related, matrix associated, actin dependent regulator of chromatin, subfamily a, member 1	0.934

Journal of Cell Science • Supplementary information

212	s24100	GTPBP4	GTP binding protein 4	0.955
213	s17117	H1FX	H1 histone family member X	0.759
214	s17118	H1FX	H1 histone family member X	0.492
215	s74	HDAC1	histone deacetylase 1	0.437
216	s75	HDAC1	histone deacetylase 1	-0.322
217	s38332	HDAC10	histone deacetylase 10	0.509
218	s38334	HDAC10	histone deacetylase 10	0.440
219	s6493	HDAC2	histone deacetylase 2	3.920
220	s6494	HDAC2	histone deacetylase 2	0.360
221	s19459	HDAC6	histone deacetylase 6	0.148
222	s19461	HDAC6	histone deacetylase 6	0.170
223	s23868	HEY1	hes related family bHLH transcription factor with YRPW motif 1	0.623
224	s23869	HEY1	hes related family bHLH transcription factor with YRPW motif 1	1.865
225	s223604	HEY2	hes related family bHLH transcription factor with YRPW motif 2	3.052
226	s23929	HEY2	hes related family bHLH transcription factor with YRPW motif 2	3.881

554	s13132	SMARCA1	SWI/SNF related, matrix associated, actin dependent regulator of chromatin, subfamily a, member 1	0.226
555	s13139	SMARCA4	SWI/SNF related, matrix associated, actin dependent regulator of chromatin, subfamily a, member 4	1.728
556	s13140	SMARCA4	SWI/SNF related, matrix associated, actin dependent regulator of chromatin, subfamily a, member 4	0.477
557	s16082	SMARCA5	SWI/SNF related, matrix associated, actin dependent regulator of chromatin, subfamily a, member 5	1.449
558	s16083	SMARCA5	SWI/SNF related, matrix associated, actin dependent regulator of chromatin, subfamily a, member 5	0.456
559	s26996	SMARCAL1	SWI/SNF related, matrix associated, actin dependent regulator of chromatin, subfamily a like 1	1.060
560	s26997	SMARCAL1	SWI/SNF related, matrix associated, actin dependent regulator of chromatin, subfamily a like 1	0.476
561	s15751	SMC1A	structural maintenance of chromosomes 1A	0.674
562	s15752	SMC1A	structural maintenance of chromosomes 1A	0.861
563	s25864	SMC1B	structural maintenance of chromosomes 1B	0.002
564	s25866	SMC1B	structural maintenance of chromosomes 1B	0.251
565	s17426	SMC3	structural maintenance of chromosomes 3	-0.790
566	s17427	SMC3	structural maintenance of chromosomes 3	-0.898
567	s25747	SND1	staphylococcal nuclease and tudor domain containing 1	-0.536
568	s25749	SND1	staphylococcal nuclease and tudor domain containing 1	2.138

Journal of Cell Science • Supplementary information

227	s223702	HEYL	hes related family bHLH transcription factor with YRPW motif-like	n.d.
228	s25474	HEYL	hes related family bHLH transcription factor with YRPW motif-like	1.687
229	s14520	HIRA	histone cell cycle regulator	-0.196
230	s14522	HIRA	histone cell cycle regulator	-0.393
231	s194488	HIST1H1E	histone cluster 1 H1 family member e	-0.766
232	s6402	HIST1H1E	histone cluster 1 H1 family member e	1.042
233	s15858	HIST1H2BM	histone cluster 1 H2B family member m	2.380
234	s194895	HIST1H2BM	histone cluster 1 H2B family member m	0.457
235	s15857	HIST2H2AC	histone cluster 2 H2A family member c	2.660
236	s194887	HIST2H2AC	histone cluster 2 H2A family member c	0.624
237	s30813	HJURP	Holliday junction recognition protein	2.485
238	s30814	HJURP	Holliday junction recognition protein	1.184
239	s30815	HJURP	Holliday junction recognition protein	0.740
240	s27105	HP1BP3	heterochromatin protein 1 binding protein 3	0.667
241	s27106	HP1BP3	heterochromatin protein 1 binding protein 3	0.722
242	s6993	HSP90AA1	heat shock protein 90 alpha family class A member 1	1.265
243	s6994	HSP90AA1	heat shock protein 90 alpha family class A member 1	-1.612
244	s6999	HSP90AB1	heat shock protein 90 alpha family class B member 1	-0.156
245	s7000	HSP90AB1	heat shock protein 90 alpha family class B member 1	1.182
246	s14373	HSP90B1	heat shock protein 90 beta family member 1	n.d.
247	s14375	HSP90B1	heat shock protein 90 beta family member 1	0.139
248	s7066	HUS1	HUS1 checkpoint clamp component	1.718
249	s7067	HUS1	HUS1 checkpoint clamp component	2.752
250	s20916	IGF2BP1	insulin like growth factor 2 mRNA binding protein 1	0.004
251	s20918	IGF2BP1	insulin like growth factor 2 mRNA binding protein 1	-0.361
252	s7246	IGHMBP2	immunoglobulin mu DNA binding protein 2	2.691

569	s813	sNEG1	-	0.000
570	s210	SRP14	signal recognition particle 14	0.033
571	s224681	SRP14	signal recognition particle 14	2.244
572	s13435	SRP9	signal recognition particle 9	1.611
573	s227428	SRP9	signal recognition particle 9	2.085
574	s13451	SRPK1	SRSF protein kinase 1	0.695
575	s224686	SRPK1	SRSF protein kinase 1	0.636
576	s12731	SRSF3	serine and arginine rich splicing factor 3	-0.016
577	s12733	SRSF3	serine and arginine rich splicing factor 3	-0.600
578	s12734	SRSF4	serine and arginine rich splicing factor 4	-1.203
579	s12735	SRSF4	serine and arginine rich splicing factor 4	1.862
580	s13490	SSRP1	structure specific recognition protein 1	2.429
581	s13491	SSRP1	structure specific recognition protein 1	2.018
582	s20074	STAG1	stromal antigen 1	0.998
583	s20076	STAG1	stromal antigen 1	1.607
584	s21089	STAG2	stromal antigen 2	-0.222
585	s21090	STAG2	stromal antigen 2	-0.096
586	s21086	STAG3	stromal antigen 3	0.374
587	s21088	STAG3	stromal antigen 3	-0.022
588	s13546	STAU1	staufen double-stranded RNA binding protein 1	0.956
589	s13548	STAU1	staufen double-stranded RNA binding protein 1	1.631
590	s14607	SUMO1	small ubiquitin-like modifier 1	1.677
591	s14608	SUMO1	small ubiquitin-like modifier 1	4.340
592	s13179	SUMO2	small ubiquitin-like modifier 2	n.d.
593	s13180	SUMO2	small ubiquitin-like modifier 2	0.743
594	s13176	SUMO3	small ubiquitin-like modifier 3	2.946

Journal of Cell Science • Supplementary information

253	s7247	IGHMBP2	immunoglobulin mu DNA binding protein 2	1.382
254	s7398	ILF2	interleukin enhancer binding factor 2	1.511
255	s7399	ILF2	interleukin enhancer binding factor 2	-0.372
256	s7401	ILF3	interleukin enhancer binding factor 3	0.345
257	s7403	ILF3	interleukin enhancer binding factor 3	2.246
258	s29255	INO80	INO80 complex subunit	1.267
259	s29256	INO80	INO80 complex subunit	0.768
260	s195940	INO80E	INO80 complex subunit E	1.927
261	s49403	INO80E	INO80 complex subunit E	-0.256
262	s16895	KAT2B	lysine acetyltransferase 2B	3.444
263	s16896	KAT2B	lysine acetyltransferase 2B	3.425
264	s18197	KIF23	kinesin family member 23	1.355
265	s225150	KIF23	kinesin family member 23	0.025
266	s22725	KIN	Kin17 DNA and RNA binding protein	-0.843
267	s22726	KIN	Kin17 DNA and RNA binding protein	1.357
268	s51989	KMT5A	lysine methyltransferase 5A	-2.080
269	s51990	KMT5A	lysine methyltransferase 5A	-1.097
270	s27462	KMT5B	lysine methyltransferase 5B	0.513
271	s27463	KMT5B	lysine methyltransferase 5B	3.379
272	s39429	KMT5C	lysine methyltransferase 5C	-0.711
273	s39430	KMT5C	lysine methyltransferase 5C	0.089
274	s35206	KRI1	KRI1 homolog	0.170
275	s35208	KRI1	KRI1 homolog	1.646
276	s21893	KRR1	KRR1, small subunit processome component homolog	1.918
277	s21894	KRR1	KRR1, small subunit processome component homolog	0.085
278	s23665	LARP1	La ribonucleoprotein domain family	-0.960
595	s13177	SUMO3	small ubiquitin-like modifier 3	-1.384
596	s13178	SUMO3	small ubiquitin-like modifier 3	2.770
597	s51833	SUMO4	small ubiquitin-like modifier 4	2.529
598	s51835	SUMO4	small ubiquitin-like modifier 4	0.282
599	s22113	SUPT16H	SPT16 homolog, facilitates chromatin remodeling subunit	-0.313
600	s22115	SUPT16H	SPT16 homolog, facilitates chromatin remodeling subunit	1.209
601	s13659	SUV39H1	suppressor of variegation 3-9 homolog 1	3.825
602	s13660	SUV39H1	suppressor of variegation 3-9 homolog 1	-0.033
603	s36183	SUV39H2	suppressor of variegation 3-9 homolog 2	3.946
604	s36184	SUV39H2	suppressor of variegation 3-9 homolog 2	2.254
605	s36185	SUV39H2	suppressor of variegation 3-9 homolog 2	1.849
606	s25811	TAF5L	TATA-box binding protein associated factor 5 like	3.026
607	s25812	TAF5L	TATA-box binding protein associated factor 5 like	3.739
608	s20882	TAF6L	TATA-box binding protein associated factor 6 like	3.013
609	s20883	TAF6L	TATA-box binding protein associated factor 6 like	1.380
610	s20824	TBL3	transducin beta like 3	2.474
611	s20825	TBL3	transducin beta like 3	0.632
612	s13991	TERF2	telomeric repeat binding factor 2	-0.478
613	s13993	TERF2	telomeric repeat binding factor 2	0.035
614	s29665	TEX10	testis expressed 10	-0.502
615	s29666	TEX10	testis expressed 10	2.170
616	s14003	TFAP2A	transcription factor AP-2 alpha	1.154
617	s14004	TFAP2A	transcription factor AP-2 alpha	0.050
618	s19395	THOC1	THO complex 1	2.505
619	s19396	THOC1	THO complex 1	1.208
620	s40362	TICRR	TOPBP1 interacting checkpoint and	-4.544

Journal of Cell Science • Supplementary information

			member 1	
279	s23667	LARP1	La ribonucleoprotein domain family member 1	1.560
280	s37861	LAS1L	LAS1 like, ribosome biogenesis factor	0.698
281	s37862	LAS1L	LAS1 like, ribosome biogenesis factor	1.517
282	s35540	LBHD1	LBH domain containing 1	2.534
283	s35542	LBHD1	LBH domain containing 1	-1.099
284	s8174	LIG1	DNA ligase 1	1.094
285	s8175	LIG1	DNA ligase 1	0.077
286	s8176	LIG3	DNA ligase 3	-0.257
287	s8177	LIG3	DNA ligase 3	0.269
288	s8179	LIG4	DNA ligase 4	-0.634
289	s8180	LIG4	DNA ligase 4	0.627
290	s38853	LLPH	LLP homolog, long-term synaptic facilitation factor	-0.009
291	s56478	LLPH	LLP homolog, long-term synaptic facilitation factor	0.739
292	s30777	LSG1	large 60S subunit nuclear export GTPase 1	n.d.
293	s30779	LSG1	large 60S subunit nuclear export GTPase 1	0.025
294	s39727	LTV1	LTV1 ribosome biogenesis factor	1.226
295	s39729	LTV1	LTV1 ribosome biogenesis factor	1.309
296	s31155	LYAR	Ly1 antibody reactive	2.653
297	s31156	LYAR	Ly1 antibody reactive	n.d.
298	s20467	MAD2L2	mitotic arrest deficient 2 like 2	-0.612
299	s20468	MAD2L2	mitotic arrest deficient 2 like 2	1.244
300	s11138	MAPK1	mitogen-activated protein kinase 1	-1.277
301	s11139	MAPK1	mitogen-activated protein kinase 1	1.582
302	s11140	MAPK3	mitogen-activated protein kinase 3	-0.110
303	s11141	MAPK3	mitogen-activated protein kinase 3	2.955
304	s17080	MBD2	methyl-CpG binding domain protein 2	0.002
			replication regulator	
621	s40363	TICRR	TOPBP1 interacting checkpoint and replication regulator	-4.049
622	s29864	TIPIN	TIMELESS interacting protein	-1.616
623	s29865	TIPIN	TIMELESS interacting protein	0.718
624	s230547	TMA16	translation machinery associated 16 homolog	0.918
625	s30718	TMA16	translation machinery associated 16 homolog	2.011
626	s9520	TONSL	tonsoku like, DNA repair protein	0.674
627	s9521	TONSL	tonsoku like, DNA repair protein	1.521
628	s14304	TOP1	DNA topoisomerase I	3.456
629	s14305	TOP1	DNA topoisomerase I	3.937
630	s14307	TOP2A	DNA topoisomerase II alpha	0.407
631	s14309	TOP2A	DNA topoisomerase II alpha	n.d.
632	s14310	TOP3A	DNA topoisomerase III alpha	1.940
633	s224746	TOP3A	DNA topoisomerase III alpha	0.529
634	s17098	TOP3B	DNA topoisomerase III beta	-0.212
635	s228087	TOP3B	DNA topoisomerase III beta	2.679
636	s606	TP53	tumor protein p53	0.755
637	s607	TP53	tumor protein p53	2.078
638	s229446	TREX1	three prime repair exonuclease 1	-0.708
639	s229448	TREX1	three prime repair exonuclease 1	1.023
640	s37740	TRMT1L	tRNA methyltransferase 1 like	0.328
641	s37741	TRMT1L	tRNA methyltransferase 1 like	2.122
642	s31336	TSR1	TSR1, ribosome maturation factor	1.157
643	s31337	TSR1	TSR1, ribosome maturation factor	1.614
644	s14556	UBA52	ubiquitin A-52 residue ribosomal protein fusion product 1	-0.333
645	s14557	UBA52	ubiquitin A-52 residue ribosomal protein fusion product 1	-1.033
646	s14558	UBA52	ubiquitin A-52 residue ribosomal protein fusion product 1	2.111

Journal of Cell Science • Supplementary information

305	s225056	MBD2	methyl-CpG binding domain protein 2	1.334	647	s228085	UBB	ubiquitin B	0.870
306	s17076	MBD4	methyl-CpG binding domain 4, DNA glycosylase	0.044	648	s338	UBB	ubiquitin B	3.265
307	s17077	MBD4	methyl-CpG binding domain 4, DNA glycosylase	2.169	649	s14560	UBC	ubiquitin C	n.d.
308	s30853	MCM10	minichromosome maintenance 10 replication initiation factor	-2.849	650	s14561	UBC	ubiquitin C	-2.934
309	s30854	MCM10	minichromosome maintenance 10 replication initiation factor	-2.243	651	s14565	UBE2A	ubiquitin conjugating enzyme E2 A	2.786
310	s8586	MCM2	minichromosome maintenance complex component 2	1.637	652	s14566	UBE2A	ubiquitin conjugating enzyme E2 A	4.606
311	s8587	MCM2	minichromosome maintenance complex component 2	-0.273	653	s14569	UBE2B	ubiquitin conjugating enzyme E2 B	2.294
312	s8590	MCM3	minichromosome maintenance complex component 3	-1.129	654	s14570	UBE2B	ubiquitin conjugating enzyme E2 B	0.432
313	s8591	MCM3	minichromosome maintenance complex component 3	-0.105	655	s14590	UBE2I	ubiquitin conjugating enzyme E2 I	5.674
314	s8593	MCM4	minichromosome maintenance complex component 4	-0.867	656	s14591	UBE2I	ubiquitin conjugating enzyme E2 I	1.055
315	s8594	MCM4	minichromosome maintenance complex component 4	0.053	657	s14723	USP1	ubiquitin specific peptidase 1	0.637
316	s8595	MCM5	minichromosome maintenance complex component 5	-0.099	658	s14724	USP1	ubiquitin specific peptidase 1	-0.343
317	s8596	MCM5	minichromosome maintenance complex component 5	0.882	659	s17367	USP10	ubiquitin specific peptidase 10	0.929
318	s8598	MCM6	minichromosome maintenance complex component 6	-0.210	660	s17368	USP10	ubiquitin specific peptidase 10	1.129
319	s8600	MCM6	minichromosome maintenance complex component 6	-1.366	661	s33026	WDR18	WD repeat domain 18	-0.018
320	s224035	MCM7	minichromosome maintenance complex component 7	n.d.	662	s33027	WDR18	WD repeat domain 18	2.737
321	s8602	MCM7	minichromosome maintenance complex component 7	-2.415	663	s21378	WDR3	WD repeat domain 3	-0.036
322	s39083	MCM8	minichromosome maintenance 8 homologous recombination repair factor	1.264	664	s21379	WDR3	WD repeat domain 3	0.156
323	s39084	MCM8	minichromosome maintenance 8 homologous	-1.186	665	s14907	WRN	Werner syndrome RecQ like helicase	-1.530

Journal of Cell Science • Supplementary information

			recombination repair factor						
324	s48537	MCM9	minichromosome maintenance 9 homologous recombination repair factor	2.028	666	s14908	WRN	Werner syndrome RecQ like helicase	-1.671
325	s48538	MCM9	minichromosome maintenance 9 homologous recombination repair factor	-0.020	667	s32337	WRNIP1	Werner helicase interacting protein 1	2.005
326	s8682	MEN1	menin 1	-0.206	668	s32338	WRNIP1	Werner helicase interacting protein 1	-0.677
327	s8684	MEN1	menin 1	1.201	669	s14925	XPA	XPA, DNA damage recognition and repair factor	0.781
328	s40987	MGME1	mitochondrial genome maintenance exonuclease 1	3.632	670	s14926	XPA	XPA, DNA damage recognition and repair factor	0.489
329	s40988	MGME1	mitochondrial genome maintenance exonuclease 1	1.607	671	s14928	XPC	XPC complex subunit, DNA damage recognition and repair factor	0.722
330	s25711	MLH3	mutL homolog 3	1.840	672	s14930	XPC	XPC complex subunit, DNA damage recognition and repair factor	1.180
331	s25713	MLH3	mutL homolog 3	3.241	673	s14952	XRCC5	X-ray repair cross complementing 5	2.251
332	s48436	MMS22L	MMS22 like, DNA repair protein	0.222	674	s14953	XRCC5	X-ray repair cross complementing 5	3.361
333	s48437	MMS22L	MMS22 like, DNA repair protein	1.679	675	s52594	XRCC6	X-ray repair cross complementing 6	1.285
334	s8898	MNAT1	MNAT1, CDK activating kinase assembly factor	0.669	676	s5456	XRCC6	X-ray repair cross complementing 6	0.445
335	s8900	MNAT1	MNAT1, CDK activating kinase assembly factor	1.202	677	s5457	XRCC6	X-ray repair cross complementing 6	2.070
336	s8960	MRE11	MRE11 homolog, double strand break repair nuclease	0.021	678	s35020	YTHDC2	YTH domain containing 2	2.548
337	s8961	MRE11	MRE11 homolog, double strand break repair nuclease	0.106	679	s35021	YTHDC2	YTH domain containing 2	-3.266
338	s30362	MRM3	mitochondrial rRNA methyltransferase 3	-1.183	680	s40699	ZNF830	zinc finger protein 830	1.075
339	s30363	MRM3	mitochondrial rRNA methyltransferase 3	-0.013	681	s40701	ZNF830	zinc finger protein 830	1.318
340	s27567	MRTO4	MRT4 homolog, ribosome maturation factor	1.057	682	s224929	ZRANB3	zinc finger RANBP2-type containing 3	1.541
341	s27568	MRTO4	MRT4 homolog, ribosome maturation factor	0.841	683	s38487	ZRANB3	zinc finger RANBP2-type containing 3	1.851
342	s30287	MTPAP	mitochondrial poly(A) polymerase	2.019					

Journal of Cell Science • Supplementary information

Table S2. Treatments/siRNAs included in the pilot screen

identifier	treatment type	target gene name	gene id	comment	control type
untreated	-	-	-	gelatin/sucrose coating only	negative
mock	transfection reagent	-	-	coating + transfection reagent	negative
sNEG1	siRNA	-	-	non-targeting siRNA	negative
sNEG9	siRNA	-	-	non-targeting siRNA	negative
sDEK	siRNA	DEK	DEK	-	transfection
APH	drug	-	-	200 nM APH	positive
CPT	drug	-	-	100 nM CPT	positive
sPOLD	siRNA	DNA polymerase δ	POLD1	catalytic subunit	positive
sPOLA	siRNA	DNA polymerase α	POLA1	catalytic subunit	positive
sKIF11	siRNA	kinesin family member 11	KIF11	microtubule motor protein	phenotypic
sAURKB	siRNA	aurora kinase B	AURKB	binds to mitotic spindle	phenotypic

Journal of Cell Science • Supplementary information

Table S3. siRNA library of the validation screen. siRNA ID from supplier (Ambion), gene IDs and names from the HUGO Gene Nomenclature Committee (HGNC) as of September 2018.

	siRNA ID	Gene ID	Full gene name	Number of DEK bodies (Z-score)		siRNA ID	Gene ID	Full gene name	Number of DEK bodies (Z-score)
1	s791	RAD50	RAD50 double strand break repair protein	0.370	15	s14305	TOP1	DNA topoisomerase I	0.731
2	s793	RAD50	RAD50 double strand break repair protein	-0.086	16	s14590	UBE2I	ubiquitin conjugating enzyme E2 I	0.636
3	s11734	RAD51	RAD51 recombinase	-0.296	17	s14607	SUMO1	small ubiquitin-like modifier 1	0.960
4	s11736	RAD51	RAD51 recombinase	-0.705	18	s14608	SUMO1	small ubiquitin-like modifier 1	0.077
5	s11941	RFC1	replication factor C subunit 1	-1.266	19	s16895	KAT2B	lysine acetyltransferase 2B	-0.236
6	s11945	RFC2	replication factor C subunit 2	-0.941	20	s16896	KAT2B	lysine acetyltransferase 2B	0.479
7	s11949	RFC3	replication factor C subunit 3	-0.511	21	s19550	SAE1	SUMO1 activating enzyme subunit 1	0.472
8	s12128	RPA1	replication protein A1	-2.019	22	s22123	POLI	DNA polymerase iota	-0.959
9	s12129	RPA1	replication protein A1	-1.494	23	s25811	TAF5L	TATA-box binding protein associated factor 5 like	0.547
10	s12131	RPA2	replication protein A2	-0.858	24	s25812	TAF5L	TATA-box binding protein associated factor 5 like	0.090
11	s13176	SUMO3	small ubiquitin-like modifier 3	0.296	25	s36183	SUV39H2	suppressor of variegation 3-9 homolog 2	0.066
12	s13178	SUMO3	small ubiquitin-like modifier 3	-0.240	26	s36184	SUV39H2	suppressor of variegation 3-9 homolog 2	-0.049
13	s13659	SUV39H1	suppressor of variegation 3-9 homolog 1	0.149	27	s36185	SUV39H2	suppressor of variegation 3-9 homolog 2	0.164
14	s14304	TOP1	DNA topoisomerase I	0.764					

Journal of Cell Science • Supplementary information

Table S4. Antibodies used in this study.

**Whole cell lysates:**		
anti-DEK rabbit polyclonal K-877*	1:20.000	F. Kappes
anti-GFP mouse monoclonal	1:1.000	Roche (1 814 460)
Goat-anti-rabbit-HRP	1:2.000	Dako Denmark (P0448)
Goat-anti-mouse-HRP	1:2.000	Dako Denmark (P0447)
Streptavidin-biotinylated-HRP	1:15.000	GE Healthcare
**DEK-IP:**		
anti-DEK mouse monoclonal*	1:500	BD (610948)
anti-SUMO-2/3/4 mouse monoclonal Clone C-3	1:500	Santa Cruz (sc-393144)
anti SUMO-1 mouse monoclonal Clone D-11	1:200	Santa Cruz (sc-5308)
Veriblot for IP Detection Reagent (HRP)	1:200	Abcam (ab131366)
Streptavidin-biotinylated-HRP	1:50.000	GE Healthcare
**6His-SUMO Ni-NTA chromatography:**		
anti-DEK rabbit polyclonal K-877	1:20.000	F. Kappes
anti-SUMO-2/3/4 mouse monoclonal Clone C-3	1:500	Santa Cruz (sc-393144)
anti SUMO-1 mouse monoclonal Clone D-11	1:200	Santa Cruz (sc-5308)
Goat-anti-rabbit-HRP	1:2.000	Dako Denmark
Goat-anti-mouse-HRP	1:2.000	Dako Denmark
Streptavidin-biotinylated-HRP	1:5.000	GE Healthcare
**In vitro SUMOylation:**		
anti-DEK rabbit polyclonal K-877	1:20.000	F. Kappes
Anti-GST mouse monoclonal	1:2.000	Santa Cruz (sc-138)
anti-SUMO-2/3 rabbit monoclonal Clone 18H8	1:500	Cell Signaling (#4971)
anti SUMO-1 mouse monoclonal Clone D-11	1:200	Santa Cruz (sc-5308)
Goat-anti-rabbit-HRP	1:2.000	Dako Denmark
Goat-anti-mouse-HRP	1:2.000	Dako Denmark
Streptavidin-biotinylated-HRP	1:25.000	GE Healthcare
**Cell fractionation & Immunoblot for EMSA:**		
anti-DEK rabbit polyclonal K-877	1:20.000	F. Kappes
anti-Histone H3 rabbit polyclonal	1:5.000	Abcam (ab1791)
**Immunofluoresence MCF10A**		
anti-DEK mouse*	1:50	Santa Cruz (sc-136222)
anti-H3K9ac rabbit	1:250	Invitrogen (710293)
anti-H3K9me3 rabbit	1:500	Abcam (ab8898)
anti-H3K27me3 rabbit	1:1000	Abcam (ab192985)
anti-lamin A rabbit	1:1000	Abcam (ab253000)
anti-PCNA rabbit	1:50	Sigma-Aldrich (HPA030522)
anti-CENP-A rabbit	1:400	Cell Signaling (2186)
2° anti-mouse Alexa Fluor 488	1:250	Invitrogen (A11001)
2° anti-rabbit Alexa Fluor 488	1:250	Invitrogen (A11008)
2° anti-rabbit Atto 532	1:200	Rockland (611-152-122)
2° anti-rabbit Atto 594	1:200	Sigma-Aldrich (77671)
2° FluoTag®-X2 IgG1 anti-Mouse Atto 647N	1:500	NanoTag Biotechnologies (N2002)
2° anti-mouse IgG F(ab')2-goat Alexa Flure 647	1:240	ThermoFisher (A21237)
*** detect DEK bodies in indirect IF**		

Journal of Cell Science • Supplementary information

Table S5. Primer sequences for the site-directed mutagenesis of the DEK primary sequence.

Target site	Primer name	Primer sequence 5' – 3'
SUMOylation site 1	SUMO1 Nr.1-fwd	CGTGGAAGGCAAGAGGGAAAAGGCAAAAGTAGAGAGG
	SUMO1 Nr. 1-rev	GCATTGTCAACCTCTCTACTTTTGCCTTTTCCCTCTTGC
	SUMO1 Nr. 2-fwd	GGAAGGCAAGAGGGAAAAGGCAGCAGTAGAGAGG
	SUMO1 Nr. 2-rev	CTTGCATTGTCAACCTCTCTACTGCTGCCTTTTCCCTC
SUMO consensus motif	SUMO3-fwd	GAGGAGCCACCAAAAAAGACAGCCGCAAGAGAAAAACCTAAAC
	SUMO3-rev	GTAGCTTTCTGTTTAGGTTTTTCTCTTGCGGCTGTCTTTTTTGG

Journal of Cell Science • Supplementary information

Table S6. Summary of cell numbers, replicates, and analysis for each experiment.

Figure	Cell type	Method; Target	# cells	Biological replicates	Analysis
Fig.1 A, B	MCF10A	IF; EdU, PCNA, DEK	124	11	Quantitative; DEK body number in MCF10A
Fig. 1 C	U2-OS / U2-OS GFP	IF, GFP-DEK, DEK	14	1	Qualitative
Fig. 2A	MCF10A	STED; DEK	11	2	Qualitative
Fig. 2B	MCF10A	STORM; DEK	4	2	Qualitative
Fig. 2 C-E	U2-OS	SIM; DEK	11	2	Qualitative
Fig. 3 A, B	MCF10A	IF; H3K9ac, DEK	21	3	Quantitative; Co-localization, Manders, t-test
Fig. 3 A, B	MCF10A	IF; H3K27me3, DEK	21	3	Quantitative; Co-localization, Manders, t-test
Fig. 3 A, B	MCF10A	IF; H3K9me3, DEK	21	3	Quantitative; Co-localization, Manders, t-test
Fig. 3C	MCF10A	PLA, H3K9me3, DEK	26	1	Quantitative;
Fig. 3D	MCF10A	PLA, H3K27me3, DEK	5	1	Quantitative;
Fig. 3 E, F, G	MCF10A	FRAP, DEK bodies	6	2	Quantitative; Mobility, t-test
Fig. 3 E, F, G	MCF10A	FRAP, DEK chromatin	14	2	Quantitative; Mobility, t-test
Fig. 4 A, B, C	U2-OS		56	2	Quantitative; DEK body number in U2-OS
Fig. 5 A, B	U2-OS	IF, control	136	13	Quantitative; t-test
Fig. 5 A, B	U2-OS	IF, CPT	15	4	Quantitative; t-test
Fig. 5 A, B	U2-OS	IF, APH	28	4	Quantitative; t-test
Fig. 6C	U2-OS	Screen	500-750 cells per siRNA. For each siRNA 2-3 wells, 4 imaging	2	Quantitative;

			positions/ well; 60-63 cells per position, cells per replicate.		
Fig. 7 F	U2-OS	WF, GFP-DEK/ GFP-SUMOmut	1600, 2000 (80 positions per cell line, ~20-25 cells per position)	2	Quantitative;
S Fig. 1 A, B, C	BJ5-TA	SIM, DEK bodies	4	2	Qualitative;
S Fig. 1 D	MCF7	IF; EdU, DEK	43	2	Qualitative;
S. Fig. 1 E	MDA-MB-231	IF; EdU, DEK	46	2	Qualitative;
S Fig. 2A	U2-OS KI eGFP-DEK	IF, PCNA, DEK	280	2	Qualitative
S Fig. 2B	U2-OS KI eGFP-DEK	IF, PCNA, DEK	33	2	Qualitative
S Fig. 2C	U2-OS	Transient expression of eGFP-DEK, RFP-PCNA, eGFP	eGFP-DEK: 23<br>RFP-PCNA: 24<br>Merge: 29<br>KI eGFP-DEK: 62	2	Qualitative
S Fig. 2D	U2-OS	Transient expression of eGFP	12	1	Qualitative
S Fig. 3 A, B, C	MCF10A	See Fig. 3A, B	93	5	Quantitative
S Fig. 4A	U2-OS KI eGFP-DEK	IF, H3K9Me3, DEK	300	2	Qualitative
S Fig. 4B	U2-OS	IF, H3K9Me3, DEK	150	2	Qualitative
S Fig. 5 A, B	MCF10A	IF, CENP-A	29	2	Qualitative
S Fig. 5 C	MCF10A	IF, Xist	27	2	Qualitative
S Fig. 5D,E	MCF10A	IF, LaminA	11	2	Qualitative
S Fig. 5 F	MCF10A	IF, DNA, DEK	13	3	Qualitative
S Fig. 5G	MCF10A	eGFP-DEK, RFP-PCNA	14	2	Quantitative
S Fig. 6 E	U2-OS KI eGFP-DEK	Screen	SNEG: 60<br>sDEK: 220<br>sUBE2I: 200<br>sRPA1: 100		Quantitative

Table S7. Parameters used in FRAP experiments and data acquisition.

	DEK-eGFP
FRAP image acquisition	512 x 512 pixels<br>40 nm pixel size<br>Excitation wavelength: 488 nm 10%<br>Emission: 500 nm - 580 nm<br>1400 Hz scan speed
Pre-photobleaching	2 frames (time/frame 0,37 s)
Photobleaching protocol	5 frames (time/frame 0,37 s);<br>2 x 2 μm square ROI; 100% 488 nm laser
FRAP imaging	90 frames (time/frame 2s); 10% 488 laser

Journal of Cell Science • Supplementary information
