# Supplementary material for: DEK oncoprotein participates in heterochromatin replication via SUMO-dependent nuclear bodies
Source: J Cell Sci. 2023 Dec 15;136(23):jcs261329. doi: 10.1242/jcs.261329 (PMC10753498; doi:10.1242/jcs.261329)
Supplement: Supplementary file 1 [file joces-136-261329_review_history.pdf]

# DEK oncoprotein participates in heterochromatin replication via SUMO-dependent nuclear bodies

Agnieszka Pierzynska-Mach, Christina Czada, Christopher Vogel, Eva Gwosch, Xenia Osswald, Denis Bartoschek, Alberto Diaspro, Ferdinand Kappes and Elisa Ferrando-May
DOI: 10.1242/jcs.261329

**Editor**: Daniel Billadeau

**Review timeline**
Original submission: 12 May 2023
Editorial decision: 26 June 2023
First revision received: 26 October 2023
Accepted: 17 November 2023

---

**Original submission**

First decision letter

MS ID#: JOCES/2023/261329

MS TITLE: DEK oncoprotein participates in heterochromatin replication via SUMO-dependent nuclear bodies

AUTHORS: Agnieszka Pierzynska-Mach, Christina Czada, Christopher Vogel, Eva Gwosch, Xenia Osswald, Denis Bartoschek, Alberto Diaspro, Ferdinand Kappes, and Elisa Ferrando-May
ARTICLE TYPE: Research Article

We have now reached a decision on the above manuscript.

To see the reviewers' reports and a copy of this decision letter, please go to: https://submit-jcs.biologists.org and click on the 'Manuscripts with Decisions' queue in the Author Area. (Corresponding author only has access to reviews.)

As you will see, the reviewers were very positive about the novelty of the study, but raise a number of substantial criticisms that prevent me from accepting the paper at this stage. In particular, all three reviewers commented on the lack of statistical analyses regarding the imaging. They suggest, however, that a revised version might prove acceptable, if you can address their concerns. If you think that you can deal satisfactorily with the criticisms on revision, I would be pleased to see a revised manuscript. We would then return it to the reviewers.

Please ensure that you clearly highlight all changes made in the revised manuscript. Please avoid using 'Tracked changes' in Word files as these are lost in PDF conversion.

I should be grateful if you would also provide a point-by-point response detailing how you have dealt with the points raised by the reviewers in the 'Response to Reviewers' box. Please attend to all of the reviewers' comments. If you do not agree with any of their criticisms or suggestions please explain clearly why this is so.

Reviewer 1

*Advance summary and potential significance to field*

© 2023. Published by The Company of Biologists under the terms of the Creative Commons Attribution License (https://creativecommons.org/licenses/by/4.0/).

This well-written manuscript by Pierzynsha-Mach et al. investigates the localization of the chromatin-bound DEK protein across the cell cycle and noted a novel localization into distinct puncta during S phase. The authors go on to describe these as “DEK bodies” and co-localize these puncta with both markers of nascent DNA (PCNA and EdU incorporation) and markers of constitutive heterochromatin (H3K9me3). They also perform a siRNA screen and identify the SUMO pathway as a modifier of DEK body formation, subsequently providing evidence that DEK can be post-translationally modified by SUMOylation. These are novel findings in our understanding of DEK functions and provide a new perspective for future studies on the role of DEK in DNA replication and chromatin formation.

*Comments for the author*

Major comments

1. The antibody used to detect DEK by immunofluorescence for DEK bodies purchased from Santa Cruz, is not used in any other applications in this manuscript. The other protocols use antibodies from BD or gifted by Ferdinand Kappes (a co-corresponding author). While the GFP-DEK fusion data is highly supportive of DEK body formation, this reviewer is curious if other DEK antibodies also detect DEK puncta in S phase, similar to the Santa Cruz antibody. It will be an important distinction for the community if only one commercially available antibody can be used to detect DEK bodies.

2. Several times, the authors comment that DEK bodies are more readily observed in primary and immortalized cells but not cancer cells. However, U2OS (osteosarcoma) cells, that are transformed and can form xenograft tumors, also create DEK bodies. The authors only chose the antibody-based method to detect DEK bodies in MCF7 and MDA-MB-231 cells. Validating that GFP-DEK also does not form DEK bodies in these cells will help validate this observation. It is interesting to note that immortalized MCF10A cells have 2-3x more DEK bodies per cell than the U2OS cancer line, and some cancer lines have no DEK bodies. This point is under-discussed. The authors also state in the Discussion that “DEK bodies were observed… in transformed, yet non-malignant cell lines.” By definition transformed cells are malignant cells, so this statement is not accurate (perhaps metastatic was the distinction between cell lines the authors were looking for not malignant?). This is also a small number of cell lines to draw such a broad conclusion. Without having a justification for potential differences in DEK body formation across cell lines (i.e.: aberrant expression or function of SUMO pathway proteins in cancers, mutation of DEK at SUMO sites in cancers, etc), it is difficult to draw any conclusions. However, this reviewer would like to highlight to the authors that the METABRIC dataset shows high positive correlation between mRNA expression levels of DEK and SUMO1/2, and other SUMOylation pathway members in breast cancers. Using publicly available datasets may help them develop some hypotheses to better address these differences in the Discussion. No additional experiments are absolutely required (although the eGFP-DEK fusion in MCF7 and MDAMB231 is requested), but this reviewer believes that at least changing the language used to describe this phenomenon is required in the revision.

3. The images from Figure 2C to 2D look over-processed. There are many rather large foci (especially for PCNA and EdU) that are completely eliminated in the reconstruction of the 3D-SIM stack. This reviewer encourages the authors to take a closer look at the image processing here, and to explain in the text why some large foci may disappear during image reconstruction
4. Many experiments have a biological sample size of only 2 replicates. In datasets with high variability, this causes some concern about the significance and reproducibility of the data. A prime example is Figure 3B, which has a high variability of colocalization between DEK and H3K9ac. Increasing replicates to permit more rigorous data analysis and interpretation is needed for several assays.

5. Many graphs representing quantified data do not have statistical analyses to support the authors’ conclusions, such as Figures 3B, 3G, 6B lower half, etc. A thorough application of statistical analyses is required. This may require increasing replicates (see point #4)

6. In the proximity ligation assay experiments in Figure 3C, there are abundant PLA signals that do not overlap with the DEK signal and the single control probe for H3K9me3 is not shown. This reviewer has low confidence that this assay was performed correctly due to what appears to be

© 2023. Published by The Company of Biologists under the terms of the Creative Commons Attribution License (https://creativecommons.org/licenses/by/4.0/). 

non-specific signal and missing controls. Adding the appropriate controls is essential for a revised manuscript.

7. The data for Figures 7 and 8 create conflicting data that the authors need to examine a little more closely. If this reviewer understood it correctly, the siRNA screen showed that the loss of the SUMO pathway INCREASED DEK bodies, but blocking DEK SUMOylation with mutations DECREASED DEK bodies. The authors very briefly include a potential hypothesis why this might happen (and it is a plausible hypothesis, that loss of SUMOylation will have a broader impact on other proteins than just DEK), but there are a few concerns to be considered here.

a. First, the authors have not confirmed that the DNA binding ability of the DEK mutant is intact. Amino acids 144 and 145 are in the region between the SAP and pseudo-SAP domains that may impact the efficiency of DNA-binding in this region. Thus, the impact of this mutant on DEK bodies may be due to impaired DNA binding, not SUMOylation deficiency.

b. Second, there still appear to be focal regions of increased signal in the DEK-SUMO mutant, they are larger but more diffuse. This reviewer recommends that the authors co-localize the GFP-DEK-SUMO mutant with PCNA or EdU as in previous figures. This will help determine if the mutant signal is still present but larger/more diffuse (potentially due to more DEK bodies, as would be expected with the siRNA data), or if the mutants truly prevent the formation of DEK bodies. This co-stain would also confirm that the images shown in the mutant are, indeed, from S phase cells. This experiment is considered essential by this reviewer for future revisions.

c. Third, using a small molecular inhibitor of SUMOylation (i.e.: TAK-981) will better support the siRNA screen that suggests SUMOylation inhibition impacts DEK body formation. Since the DEK mutant and SUMO pathway knockdown data are conflicting, adding an additional method, like TAK-981, may help resolve the discrepancy.

Minor Comments (not related to experiments)

1. In the introduction, the authors mention a few times that “DEK is highly enriched at maturing chromatin” but no references are included to support this statement.

2. In the “DEK bodies are novel players…” section of the Discussion, the authors state “In this study, over-expression of KAT2B [histone acetyltransferase] led to an accumulation of DEK in interchromatin granule clusters (IGCs)…” but data from KAT2B experiments are NOT included in this manuscript draft. Please either add the data or remove this statement.

3. In Figure 4, the authors mention that DEK bodies can be in close proximity to the nuclear envelope and nuclear invaginations. The relevance of this observation needs to be described.

Reviewer 2

*Advance summary and potential significance to field*

The authors identified an accumulation of the oncoprotein DEK at late DNA replication sites, which correspond to constitutive heterochromatin. How DNA embedded in the complex structure of heterochromatin is replicated is a very active field of research, as it is also associated to the epigenetic memory of these regions. Finding a new factor potentially involved in the replication of heterochromatin is, therefore, of interest for the field, especially since its accumulation at late replicating sites seems to be modulated by SUMO, a protein already found to accumulate at these sites in other systems. The findings from this study could definitely participate to a better understanding of the mechanisms underlying the replication of heterochromatin and give new directions for investigating this process.

*Comments for the author*

I found the article very interesting and finding DEK at heterochromatin replication sites could open open new ways to investigate the mechanisms underlying the replication of DNA in the complex structures of dense heterochromatic regions. However, before publication, several points should be addressed and/or clarified, in particular regarding the role of SUMOylation in the formation of the DEK bodies (last part, see specific points below). Also, since SUMO is an important factor for the formation of PML bodies and since PML bodies were also found at late replication sites

© 2023. Published by The Company of Biologists under the terms of the Creative Commons Attribution License (https://creativecommons.org/licenses/by/4.0/).

in other systems, the authors should definitely look at the overlap between the DEK and the PML bodies.

I enjoyed reading the imaging part of the manuscript. However, the authors use different types of super-resolution microscopy (STED, dSTORM, 3D-SIM) but why they chose some and not others depending on the context is not discussed at all. It may be obvious for microscopists, but this should be briefly explained, at least in the Material and Methods section.

I am still unsure after reading the manuscript what the DEK bodies do contain, in particular regarding PCNA and H3K9me3. To me, it looks like that they form a heterogenous population, but this is almost not discussed by the authors. And this information is missing since experiments such as FRAP were done at the single body level (see below for more details).

There is not a single statistical analysis, which is unfortunate as we can not assess the robustness of the analyses and the significance of the differences. This should be done at several places.

I also think that the way some results are presented is too "shallow" and the reader has to find the information within the figures and the rational by themselves. Some results are actually discussed in the discussion without being presented in the results section.

I will be glad to review a revised manuscript.

**Specific points**

**Results**

***A sub-population of DEK molecules assembles into complex nuclear bodies at sites of late DNA replication***

*Page 7 "A sub-population of DEK molecules assembles into complex nuclear bodies…"*. Why complex?

*Legend of Figure 1A " Average number of DEK foci per cell"*. In which cells? (late S phase I would guess?) Also, *"Wildfield images"* is indicated in the legend for panel A and D, but not B and C. In my opinion, *"Widefield images of MCF10A cells"* should not only be for panel A, but also for panels B and C (A-C).

*Figure 1B and 1C*. Figure 1C shows the distribution of PCNA and EdU in addition to DEK in MCF10A cells, even though not mentioned in the legend (in the text, it is mentioned either one or the other). Why then having a separate panel for EdU alone (1B)? Couldn't the authors show the fluorescence intensity profile corresponding to EdU along those corresponding to PCNA and DEK, and then get rid of panel B? Alternatively, EdU should not be shown in panel C.

*Legend of Supplementary Figure S1*. The average number of DEK foci per cell should be indicated for each cell type, as in Figure 1.

*Legend of Figure 2A-B*. For 2A, the legend says *"The STED image reveals a granular internal structure of DEK bodies (insets)"*. What does the *"magnified image of the ROI shown in the middle containing a DEK body"* show in 2B? Did the use of two types of super-resolution microscopy allow the authors to reveal different structures, or do they simply support each other?

*Page 8 " […] followed by 3D Structured Illumination Microscopy (3D-SIM)"*. Not clear how this was done in the Material and Methods section (as opposed to STORM).

*DEK bodies assemble at replicating heterochromatic regions*

*Page 8 "The occurrence of DEK in a subpopulation of PCNA- and EdU-positive nuclear bodies in late S-phase"*. This is an interesting observation. Not all EdU/PCNA foci (nuclear bodies) are found enriched in DEK (according to Fig.1A-C). How about the opposite? Do all DEK foci "contain" PCNA and/or EdU? When looking at Fig.1B, it does not seem so. Could the authors comment on that and maybe provide a quantitative analysis indicating how many DEK foci (% ?) are associated with the

© 2023. Published by The Company of Biologists under the terms of the Creative Commons Attribution License (https://creativecommons.org/licenses/by/4.0/). 

two other types of foci? And vice versa? Since they calculated the average number of DEK foci per cell (6.7 ± 2.2, n=46 cells, Fig.1), this should be easily doable.

*Figure 3A*. The intensity profiles of EdU should be added to each graph (as in Figure 4A), to show that the authors are looking at replicating chromatin and to support their conclusion. It is actually true for all graph showing intensity profiles in late S phase.

*Page 8 "This conclusion was corroborated by Manders' coefficient analysis"*. I am not sure to understand how this analysis was performed. What does n represent? (*n=8 from two independent experiments*). This number (8) seems rather small (the upper whisker for H3K9ac goes above the one for H3K27me3). Which DEK foci were analyzed? From what I can see on the figures, not all foci contain H3K27me3 or H3K9me3. This is not clear and should be better explained.

*Page 8, FRAP experiment*. While I understand how the acquisitions were made and how the mean fluorescence intensity within the bleached region in each case was monitored, I have a problem understanding how the normalization was done. The authors write in their methods (page 23) *"the curves were combined and normalized to the minimum and maximum of the combined curve"*. What is the combined curve? Shouldn't each curve be normalized according to their own maximum/minimum? (so the maximum is always 1 and, in that case, the minimum 0). Actually, the authors write page 22 *"all traces were min/max normalized, averaged and the standard error was calculated."*, which is what I would expect from such an analysis. I am confused about what was done. Also, what does *"Finally, each of the curves was fit"* mean (Page 23)? Also, according to the different images provided by the authors showing both DEK (either endogenous or overexpressed) and H3K9me3 distribution patterns, not all DEK bodies seem to contain H3K9me3. So how were the bleached foci selected? The legend for Figure 3F says *"Data points show the average of 6 experiments for DEK bodies [...]"*. Does that mean that 6 FRAP experiments were done? Or one experiment with 6 cells cells? Were the bleached foci from different nuclei? If, as I mentioned previously, DEK bodies are not systematically enriched in H3K9me3, how to be sure that the bodies the authors looked at by FRAP are really associated with heterochromatin? Information should be provided about the occurrence of H3K9me3 and DEK-EGFP foci colocalization in MCF10A when DEK-EGFP is transiently expressed (only images in U2-OS cells overexpressing eGFP-DEK is provided in Supplemental Figure S4). If several types of DEK bodies (with and without H3K9me3) do co-exist within a given nucleus, then "6" is not enough to conclude anything. The authors could bleach several (or all) DEK foci from the same nucleus and verify if they all show the same type of recovery curve (or not). If all eGFP-DEK bodies in transfected MCF10A do contain H3K9me3, then this should be clearly shown. Finally, the eGFP-DEK foci (focus) bleached in Figure 3E is large, close to the nuclear periphery, and resembles to the one associated with H3K27me3 in Figure 3A. Is it representative of all DEK bodies? For all the aforementioned reasons, I find it difficult to draw a conclusion from the results of this experiment.

*Page 8, "In line with these data, DEK bodies were associated with heterochromatin, in particular with H3K9me3, also in U2-OS wild type and U2-OS KI eGFP-DEK cells (Fig. S4)."* As mentioned above, not all DEK bodies seem to contain H3K9me3 (hence my comments on the FRAP experiment). The line drawn in panel C exclude one foci (focus) that most likely does not contain H3K9me3. The authors should be more precise when presenting their results and mention whether all DEK bodies contain H3K9me3 or not.

*Page 9, "Maximum intensity projections of confocal Z-stacks revealed that the epigenetic mark for centromeres, the histone H3 variant CENP-A, reproducibly decorates DEK bodies from both sides (Fig. 4A)"*. How were the top and bottom of the Z-stacks defined? Do they correspond to the top and bottom of the DEK bodies only, or are fluorescent signals above and under these bodies also captured? The reason I am asking this is because I do not understand how/why in 3D, CENP-A signals would flank the DEK-bodies only along the x/y axes. This would be easily explained if the z-stacks only covered in z the volume of the DEK bodies and ignored what is above and under. However, if the stacks also contain signals located above and under the bodies, then why do we not see CENP-A in the middle of some bodies, since we look at max projections along the z-axis? This should be clarified either here or in the methods section. This is also the case when analyzing the staining of the Barr body in regards to the DEK bodies (Figure 4C). And again, are all DEK bodies decorated by CENP-A?

© 2023. Published by The Company of Biologists under the terms of the Creative Commons Attribution License (https://creativecommons.org/licenses/by/4.0/). 

*Figure 4A*. Four ROI are drawn, why? One has an asterisk, which is the one shown under I think. But no indication in the legend.

*Page 9, "We did not observe spatial proximity of DEK and CENP-A outside of late S (Fig. 4B)"*. In Figure 4B, the authors selected one CENP-A foci (focus) which does not overlap with DEK. However, I can see at least two white foci indicating a colocalization, at this level of resolution, between DEK and CENP-A. Can the authors comment on that?

*Page 9, "Similarly, we found DEK bodies in close proximity of the nuclear periphery and Lamin-A positive nuclear invaginations only in S-phase (Fig. 4D, E)"*. When a nuclear lamina invagination (lamin A staining) is observed, is DEK systematically present within close proximity? The number of invaginations usually differs from nucleus to nucleus.

*Page 9, " DEK bodies corresponded to regions of dense chromatin, as shown by DNA staining with ToPro3 (Fig. 4F)"*. In the magnified image, why did the authors draw a line "along the white area" where the two signals (DNA and DEK) overlap, and not along the perpendicular direction, as they did for H3K27me3 to show a juxtaposition instead of a colocalization? This should be fixed or explained.

*Page 9, "Altogether, our collective microscopy data strongly support a role for DEK bodies in the replication of heterochromatic chromatin regions in late S-phase."* I agree, even though I think more precision should be added in some parts of the results.

***DEK body dynamics is affected by DNA replication inhibitors***

*Page 9, " time-lapse imaging experiments <u>at</u> a spinning disk confocal microscope"*. Should be "with" or "using".

*Page 9, " 2 ± 18% of DEK bodies colocalized with PCNA-marked replication foci in these cells (object-based analysis of 19 DEK bodies)"*. Why was the JaCoP plugin used here while the number of DEK bodies was assessed manually in U2-OS cells? Also, why only 19 bodies analyzed, which correspond to 3 cells, according to "6.7±2,2 bodies per nucleus" (out to 13, according to the supplementary figure S5's legend).

*Page 10, "Of the three substances, aphidicolin showed the most prominent effect reducing DEK body number by 47%, and prolonging DEK body lifetime by 28%."* The number of foci being relatively low in the control, the authors should give numbers for the different conditions and compare them instead of indicating by which percentage they differ. Also, a statistical analysis should be performed in figure 6B (and at several other places) to address the significance of the observed differences. Between 15 and 132 cells were analyzed, which is a very broad window. The exact number for each condition should be indicated somewhere (in figure, legend or methods).

*Page 10, " Camptothecin had a similar but more moderate effect, and hydroxyurea did not alter DEK body dynamics"*. The experiment was done once and HU did not seem to have much effect on the duration of G2, as opposed to the other inhibitors. How can the authors be sure that they used HU in the proper condition? I think that the data provided here are not sufficient for the author to conclude that their results *"<u>strongly suggests</u> that DEK bodies are implicated in the replication of DNA structures particularly susceptible to aphidicolin"*. The significance of the differences is important to assess, as APH and CPT are further used in the positive controls for the siRNA screen.

***Down-regulation of the SUMO pathway increases DEK body number in an siRNA screen for DEK body regulators***

*Supplementay Matetrial and Methods, Page 27*. What is COC? Where are the COC imaging from?

*Supplementay Matetrial and Methods, Page 29. […] these track identifiers were used to summarize relevant measurements such as DEK body lifetime, number of DEK bodies per track etc."* The DEK body lifetimes and number of DEK bodies were previously used by the authors. What were the other relevant parameters? Only the cited ones are shown in *Supplemental Figure S6*.

© 2023. Published by The Company of Biologists under the terms of the Creative Commons Attribution License (https://creativecommons.org/licenses/by/4.0/).

*Supplementay Matetrial and Methods, Page 30, "[…] and the mean ($\underline{X}$sNEG1)…"*. Should be $\bar{\underline{X}}$ sNEG1. Also $X_i$ should be defined.

*Page 11, "[…] corresponding to 44 and 28 different genes, respectively (Fig. 7B, C)" and Figure 7B.* The authors should provide an exhaustive list of all up-regulators.

*Page 11, " we conducted a low-throughput secondary screen with 28 siRNAs (Fig. 7D)"*. Only 20 siRNAs are shown in the figure. Where do the authors show the effects of the remaining 8? Since it is a validation screen, one would expect to see them all.

*Page 11, " Still, the effect of up- and down-regulation of DEK body number was confirmed (Fig. 7D)".* Not for SUMO3, based on Figure 7D, right graph. The authors should mention it and comment, since they focus on SUMO3 later on. Actually, the statement *"the secondary screen validated four top DEK body up-regulators from the primary screen belonging to the SUMO pathway, among them the E1 activating enzyme SAE1 and the E2 conjugating"* is incorrect, if SUMO3 is part of them (which I believe it is, considering the last part of the results section). The authors should refine the analysis of their validation screen. Statistics?

*Page 11, " Our screening approach thus determined that the SUMO pathway plays a major role in the regulation of DEK body formation."*. In my opinion, this statement is too strong at this stage and should be tuned down.

***DEK is a SUMO target and SUMOylation is required for the formation of DEK bodies***

*Page 11, " we focused on SUMO 1 and 3, as both were strong hits in the screens."* As for my previous comments, SUMO 3 is not a strong hit based on the secondary (validation) screen.

*Figure 8A*. The authors should indicate on the figure or in the legend which antibody was used for IP. Should be against DEK, according to the main text and the material and methods. As it stands now in the legend, one cannot know what antibody was used for IP and/or detection. Also, I find it striking how the three immunoblots look alike, with a main band around 50 kDa in the IP lane and a faint one around 35 kDa for all of them. Is the faint band due to the secondary antibody used? Or a degradation product? Finally, why the authors do not detect any SUMO in the inputs?

*Page 11, "showing direct interaction of DEK with SUMO1 and SUMO2/3 (Fig. 8A)"*. If I read the figure correctly, the band that the authors detect using SUMO1 and SUMO2/3/4 antibodies correspond to DEK. Then it is not really a direct interaction, as we would expect from an interaction between the SIM (SUMO-interacting motif) of a protein and SUMO. The authors should rephrase this sentence. Also, according to the immunoblot performed after DEK IP and using antibodies against DEK for detection, there is only one band corresponding to DEK. Do the authors believe that DEK is always SUMOylated? Should we not expect several bands according to the level of SUMOylation otherwise? And when looking at the next panel (B), I am not sure to understand what we are looking at in these immunoblots (see my next comment).

*Figure 8B*. After spending a substantial amount of time on it, I do not understand the differences in terms of observed bands between these immunoblots and the ones presented in Figure 8A. Does the reader have to make their own interpretations of these results? What are we looking at in the middle and right immunoblots? Shouldn't we see several bands for SUMO in Figure 8A as well after DEK pull down? This series of experiments and their interpretations (corresponding to Figure 8A and 8B) should be better explained.

*Legend of Figure 8C, "Densitometric quantification of mono-SUMOylated DEK signals from (B, left panel). DEK signals in pulldown samples were normalized to the corresponding input samples."* How was this done? Which signals correspond to "mono-SUMOylated DEK". No information is provided anywhere as far as I could see. Also, there are some statistics provided. What are they? How was this done? How many experiments were performed?

© 2023. Published by The Company of Biologists under the terms of the Creative Commons Attribution License (https://creativecommons.org/licenses/by/4.0/).

*Page 12, "we screened the DEK sequence for additional potential SUMOylation sites, which revealed amino acids 61, 62 and 144, 145 as suitable SUMO attachment sites."* How did the authors identify these sites? This should be indicated.

*Figure 8D.* The authors should show immunoblots with anti-SUMO-1 and anti-SUMO-2/3 as they did in Supplementary Figure 8.

*Page 12, "[…] highlighting that most of the potential SUMOylation sites were captured by our mutational approach".* Difficult to assess, since the authors do not provide information about how they did identify the other SUMOylation sites (see previous comment). There are maybe more sites, which are SUMOylated under specific conditions. This statement should be tuned down.

*Page 12, " No DEK body formation was observed in cells expressing SUMO-less DEK (GST- DEK SUMO mut, Fig. 8F)."* Actually, the whole DEK distribution pattern within the nucleus seems to be different between the WT and the mutant DEK. Indeed, even at this level of resolution, it seems that the mutant DEK is not really bound to anything anymore and accumulate in sub-compartment which I would instinctively identify as nucleolus. The fact that the authors do not observe DEK bodies with this mutant maybe due to something more general than just SUMOylation. The sites that the authors have identified as potential SUMO targets may be involved in other mechanisms that are independent of SUMO. Maybe these sites are not directly SUMOylated but rather involved in protein-protein (or protein-DNA?) interactions which are necessary for the SUMOylation of DEK? Is the mutant DEK even able to bind chromatin? Is it still "functional"? Based on the images in Figure 8F, I suspect a large fraction of the DEK mutant to be soluble. Several ways to verify this exist and should be easy to put in place, including a FRAP experiment in HeLa cells expressing wt or mutant DEK to look at the "chromatin" fraction, or a simple cell fractionation to look at the soluble fraction of DEK compared to the insoluble one. The authors should also provide pictures corresponding to DNA staining in Figure 8F, so one can rule out (or not) the accumulation of the DEK mutant in the nucleolus. Finally, what about the formation of endogenous DEK bodies when the mutant is expressed? (in other words, is there a dominant negative effect?)

*Page 12, " where SUMOylation of DEK seems to be required for DEK body formation".* For the reasons I mentioned above, I think that more evidence should be provided to show that it is really the lack of DEK SUMOylation that is responsible for the absence of DEK bodies in the last experiment.

**Discussion**

I found the discussion interesting to read and informative. However, I would like the authors to answer my comments regarding the role of SUMOylation of DEK in the process of DEK body formation before commenting on the last part of the discussion dealing with SUMO.

*Page 15, " DEK body up-regulating siRNAs show a remarkable enrichment in proteins involved in post-translational modification pathways. Histone acetyltransferases (HATs) ACTL6A, KAT2B, TAF5L and TAF6L, histone deacetylase HDAC2, histone methyltransferases SUV39H1/2 and SUMOylation-related proteins UBE2I, SUMO1 and SAE1 are among the top hits."* Yes, this is interesting. But why did the authors not mention it in the results section? As mentioned before, the presentation of results should be improved at several places.

*Page 16, " SUMO-less DEK does not form bodies."* I have already commented on that earlier, and I am not convinced that one can conclude this at the moment.

*Page 16, " This knowledge aligns well with our screening data, where downregulation of both SUV39H1 and SUMO increase the number of DEK bodies".* Sorry, but I do not understand how. Also why is SUV39H1 not mentioned in the results part at all? Back to previous comments regarding the way some results are presented.

Reviewer 3

*Advance summary and potential significance to field*

© 2023. Published by The Company of Biologists under the terms of the Creative Commons Attribution License (https://creativecommons.org/licenses/by/4.0/).

The authors have investigated the subcellular localization of the DEK oncoprotein. Using immunofluorescence microscopy and cultured mammalian cells, they found that DEK localizes to distinct nuclear focal assemblies specifically in early to mid S-phase cells, which they term DEK bodies. Using co-localization studies, they demonstrated that DEK bodies exist in close proximity to active replisomes and are associated with markers of constitutive heterochromatin. Using a high-throughput screen, they identified factors affecting DEK body formation, including multiple regulators of sumoylation. Using in vivo and in vitro assays, they demonstrated that DEK is sumoylated at multiple lysine residues. A mutant of DEK with reduced sumoylation failed to form DEK bodies when expressed in cells. The authors conclude that DEK is important for heterochromatin integrity and that its function is regulated through sumoylation.

DEK is an oncoprotein with functions associated with DNA replication and replication stress. Its precise functions are not fully characterized, and associations with discrete chromatin domains and nuclear foci has not been previously described. The findings reported in this study are therefore novel and provide new insights into DEK function that may be relevant to better understanding its role in oncogenesis. Overall, the manuscript is well written and the data are mostly of high quality.

*Comments for the author*

There are two major issues that need to be addressed:

(1) Much of the fluorescence microscopy data is presented as a single cell. Quantitative, population-based data would strengthen the conclusions. Specific questions that should be addressed include: Figure 1 - (1) what fraction of S-phase cells show DEK bodies?, (2) what fraction of Edu and PCNA positive foci are associated with DEK bodies? Figure 2 - (1) is the data supported by analysis of multiple DEK bodies analyzed across multiple cells? Figure 3 - (1) with each marker (H3K9ac, H3K27me3, and H3K9me3) what fraction of DEK foci show no overlap, spatial juxtaposition or colocalization? This question is relevant given that in the image in 3A showing DEK:H3K9me3 there are DEK foci showing colocalization (the one analyzed) and juxtaposition. (2) The Manders' coefficients analysis should be explained. Figure 4 - (1) what fraction of DEK bodies associate with centromeres, lamin invaginations and Xist, and vice versa?

(2) There are a number of issues with the sumoylation data in Figure 8. (1) In Figure 8A, here are no, or barely detectable, SUMO signals in the input lanes for the anti-SUMO1 and SUMO2/3/4 blots, raising a concern about the effectiveness of these antibodies. In addition, it is odd that the bands in the IP lanes for the anti-DEK, SUMO1 and SUMO2/3/4 blots are all of similar molecular weights (sumoylation at one site should cause a ~15 kDa shift in DEK, but this does not appear to be the case). There is a concern that the signals in the IP lane correspond to antibody heavy and light chains. Were similar amounts of control and DEK antibodies used - Ponceau S stained blots showing antibody chains in control and DEK antibody IPs would help. (2) In Figure 2B, there are no or barely detectable high molecular weight signals corresponding to SUMO2 or SUMO3 conjugated proteins seen in the anti-SUMO2/3/4 blot in either the input or the pulldowns.
This raises suspicions about the high molecular weight DEK bands detected in the anti-DEK blot. The presence of such high molecular weight bands is also inconsistent with results presented in 8A. Results in Figure 8F should include quantification across multiple cells. It would also be helpful to do co-localization with Edu or PCNA to verify that there are S phase cells present in the mutant expressing cells. It also appears that the mutant protein is concentrated in nucleoli, unlike the wild type protein. This should also be noted and quantified.

Lastly, it is a stretch to conclude that the observed defect in DEK body formation is due to a defect in DEK sumoylation. Lysine to arginine substitutions are also expected to disrupt possible ubiquitylation, acelylation and methylation at these sites. Can the defect be rescued with a DEKmutant-SUMO fusion protein?

Collectively, these issues raise serious concerns about the role that DEK sumoylation may play in affecting DEK body formation. Moreover, the findings that more DEK bodies are formed when sumoylation is inhibited is inconsistent with the DEK SUMO mutant result and suggests that other substrates may be more relevant.

© 2023. Published by The Company of Biologists under the terms of the Creative Commons Attribution License (https://creativecommons.org/licenses/by/4.0/).

**First revision**

Author response to reviewers' comments

**Point-by-point response to the Reviewers:**

*For enhanced clarity and cross-referencing purposes between reviewer comments, we added consecutive numbering to questions from reviewers 1, 2 and 3.*

**Reviewer 1 Advance Summary and Potential Significance to Field:**

This well-written manuscript by Pierzynsha-Mach et al. investigates the localization of the chromatin-bound DEK protein across the cell cycle and noted a novel localization into distinct puncta during S phase. The authors go on to describe these as “DEK bodies” and co-localize these puncta with both markers of nascent DNA (PCNA and EdU incorporation) and markers of constitutive heterochromatin (H3K9me3). They also perform a siRNA screen and identify the SUMO pathway as a modifier of DEK body formation, subsequently providing evidence that DEK can be post-translationally modified by SUMOylation. These are novel findings in our understanding of DEK functions and provide a new perspective for future studies on the role of DEK in DNA replication and chromatin formation.

**Response:** We would like to thank this reviewer for the positive comments on our work and the very thoughtful and helpful suggestions which we addressed below.

**Reviewer 1 Comments for the Author:**

**Major comments**

1.1. The antibody used to detect DEK by immunofluorescence for DEK bodies, purchased from Santa Cruz, is not used in any other applications in this manuscript. The other protocols use antibodies from BD or gifted by Ferdinand Kappes (a co-corresponding author). While the GFP- DEK fusion data is highly supportive of DEK body formation, this reviewer is curious if other DEK antibodies also detect DEK puncta in S phase, similar to the Santa Cruz antibody. It will be an important distinction for the community if only one commercially available antibody can be used to detect DEK bodies.

**Response:** We thank the reviewer for raising this indeed important detail, especially considering well-established scenarios where certain pan-specific DEK antibody preparations show selective reactivity to certain post-translational modifications on DEK. We did not systematically test a larger set of commercially available DEK antibodies for their specific reactivity towards DEK bodies. However, we can confirm that at least three different DEK-specific antibody preparations, including the Santa-Cruz antibodies, reproducibly detected focal assemblies of DEK in primary, immortalized and, in some cases, also in transformed cell lines (like U2-OS). Specifically, the polyclonal DEK antibody from F. Kappes (K-877 Ab) has been used in indirect immunofluorescence experiments in Fig. 1C (former Fig. 1D) upper panel (U2-OS wild-type), in Fig. 2 C and in Supplementary Figure Fig. S1 A and detected DEK bodies. DEK-specific monoclonal antibodies from BD also detected DEK bodies under several conditions (collective work of our labs over the past decade; data not shown). In fact, one of the very first recognition of DEK foci in our labs, which was a curious observation back then, was achieved by the BD antibody (Figure 24; Anja Tabbert, Dissertation, University of Konstanz, 2004; https://kops.uni-konstanz.de/entities/publication/bf4a6eaa-807f-4677-b6b7-d8cbaeb31e86).

As DEK-antibody specificity is indeed an important consideration for the community, we now clearly indicated that all used antibodies in this study detect DEK bodies in indirect immunofluorescence in Supplementary Table 3. For additional clarity, we also added information about the employed antibody to the respective figure legends.

© 2023. Published by The Company of Biologists under the terms of the Creative Commons Attribution License (https://creativecommons.org/licenses/by/4.0/).

**1.2.** Several times, the authors comment that DEK bodies are more readily observed in primary and immortalized cells but not cancer cells. However, U2OS (osteosarcoma) cells, that are transformed and can form xenograft tumors, also create DEK bodies. The authors only chose the antibody-based method to detect DEK bodies in MCF7 and MDA-MB-231 cells. Validating that GFP-DEK also does not form DEK bodies in these cells will help validate this observation. It is interesting to note that immortalized MCF10A cells have 2-3x more DEK bodies per cell than the U2OS cancer line, and some cancer lines have no DEK bodies. This point is under- discussed. The authors also state in the Discussion that “DEK bodies were observed… in transformed, yet non-malignant cell lines.” By definition, transformed cells are malignant cells, so this statement is not accurate (perhaps metastatic was the distinction between cell lines the authors were looking for, not malignant?). This is also a small number of cell lines to draw such a broad conclusion. Without having a justification for potential differences in DEK body formation across cell lines (i.e.: aberrant expression or function of SUMO pathway proteins in cancers, mutation of DEK at SUMO sites in cancers, etc), it is difficult to draw any conclusions. However, this reviewer would like to highlight to the authors that the METABRIC dataset shows high positive correlation between mRNA expression levels of DEK and SUMO1/2, and other SUMOylation pathway members, in breast cancers. Using publicly available datasets may help them develop some hypotheses to better address these differences in the Discussion. No additional experiments are absolutely required (although the eGFP-DEK fusion in MCF7 and MDAMB231 is requested), but this reviewer believes that at least changing the language used to describe this phenomenon is required in the revision.

**Response:** We thank this reviewer for raising this critical point. Given that all employed DEK-specific antibodies reliably and robustly detected DEK bodies (see point 1.1 above), we originally hypothesized that assessing GFP-DEK fusions in the transformed cell lines would not yield any deeper insights at this point, as we could not, or very sparsely detect DEK bodies using indirect immunofluorescence. We agree that a systematic assessment of DEK body formation and dynamics using antibodies and GFP-DEK fusions in a larger panel of primary, immortalized and tumor cells would certainly yield interesting insights. However, as we are currently not in the position to conduct such an extensive study, we took the advice from the reviewer and carefully reworded applicable sections. The previously erroneous statement has also been revised and toned down appropriately. We again thank this reviewer for pointing out these shortcomings.

**1.3.** The images from Figure 2C to 2D look over-processed. There are many rather large foci (especially for PCNA and EdU) that are completely eliminated in the reconstruction of the 3D-SIM stack. This reviewer encourages the authors to take a closer look at the image processing here, and to explain in the text why some large foci may disappear during image reconstruction

**Response:** We thank this reviewer for raising this point. The appearance of over-processing derives from the way data have been analyzed in the SIM workflow, which was not appropriately explained in the methods section in the original submission. Specifically, the SIM images depicted on panel 2C and 2D were visualized in a 3D SIM mode by acquiring a z-stack with 20 slices (0.125 µm step size in z). Pseudo-widefield images were generated with Fiji software. Figure 2, panel C shows the pseudo-widefield representations (all z-stacks), while panel D only shows a single z-stack from a middle section of the super-resolved image stack after the reconstruction. Below, the magnified inset shows a single DEK positive large replication focus. However, we re-visited the relevant micrographs in Figure 2D and re-analyzed them. We also added more details to the figure legend and added a section to the Methods part explaining the details of this process, which was missing prior.

**1.4.** Many experiments have a biological sample size of only 2 replicates. In datasets with high variability, this causes some concern about the significance and reproducibility of the data. A prime example is Figure 3B, which has a high variability of colocalization between DEK and H3K9ac. Increasing replicates to permit more rigorous data analysis and interpretation is needed for several assays.

**Response:** We thank this reviewer for highlighting our shortcomings and nudging us to re-visit many aspects of data analyses, which was also pointed out by reviewer 2 (2.3; 2.4; 2.11; 2.13; 2.23) and 3 (3.2; 3.4).

We now re-analyzed most experiments, increased the number of cells and/or replicates where

© 2023. Published by The Company of Biologists under the terms of the Creative Commons Attribution License (https://creativecommons.org/licenses/by/4.0/).

possible and conducted additional statistical analyses. A full summary of cell numbers per experiment including biological replicates is given in Table R1 below (also provided as Supplementary Table 6 in the revised version).

Specifically, for Fig. 3B we analyzed a total of 21 cell nuclei for each condition from three biological replicates (n = 8, n = 8 and n = 5, for DEK&H3K9ac, DEK&H3K27me3 and DEK&H3K9me3, respectively). The total number of DEK bodies analyzed from these nuclei and comprising Figure 3B totals to 101 (29 for DEK&H3K9ac, 37 for DEK&H3K27me3 and 35 for DEK&H3K9me3). Statistical analysis between conditions was calculated using t-test after establishing the variance differences by f-test and applying post-hoc Bonferroni correction. This established statistical differences between the tested conditions with p = 0.0078368 (H3K27ac and H3K27me3), p = 4.37904E-07 (H3K9ac and H3K9me3) and p = 9.19623E-08 (H3K27me3 and H3K9me3). This information has been added to the figure legend.

**Table R1:** Summary of cell numbers, replicates, and analysis for each experiment.

| Figure | Cell type | Method; Target | # cells | Biological replicates | Analysis |
|---|---|---|---|---|---|
| Fig.1 A, B | MCF10A | IF; EdU, PCNA, DEK | 124 | 11 | Quantitative; DEK body number in MCF10A |
| Fig. 1 C | U2-OS / U2-OS GFP | IF, GFP-DEK, DEK | 14 | 1 | Qualitative analysis |
| | | | | | |
| Fig. 2A | MCF10A | STED; DEK | 11 | 2 | Qualitative analysis |
| Fig. 2B | MCF10A | STORM; DEK | 4 | 2 | Qualitative analysis |
| Fig. 2 C-E | U2-OS | SIM; DEK | 11 | 2 | Qualitative analysis |
| | | | | | |
| Fig. 3 A, B | MCF10A | IF; H3K9ac, DEK | 21 | 3 | Quantitative; Co-localization, Manders, t-test |

© 2023. Published by The Company of Biologists under the terms of the Creative Commons Attribution License (https://creativecommons.org/licenses/by/4.0/).

| | | | | | |
|---|---|---|---|---|---|
| Fig. 3 A, B | MCF10A | IF; H3K27me3, DEK | 21 | 3 | Quantitative; Co-localization, Manders, t-test |
| Fig. 3 A, B | MCF10A | IF; H3K9me3, DEK | 21 | 3 | Quantitative; Co-localization, Manders, t-test |
| Fig. 3C | MCF10A | PLA, H3K9me3, DEK | 26 | 1 | Quantitative; |
| Fig. 3D | MCF10A | PLA, H3K27me3, DEK | 5 | 1 | Quantitative; |
| Fig. 3 E, F, G | MCF10A | FRAP, DEK bodies | 6 | 2 | Quantitative; Mobility, t-test |
| Fig. 3 E, F, G | MCF10A | FRAP, DEK chromatin | 14 | 2 | Quantitative; Mobility, t-test |
| **Fig. 4 is now Supplementary Figure S5** | | | | | |
| Fig. S4 A, B | MCF10A | IF, CENP-A | 29 | 2 | Qualitative |
| Fig. S4 C | MCF10A | IF, Xist | 27 | 2 | Qualitative |
| Fig. S4D,E | MCF10A | IF, LaminA | 11 | 2 | Qualitative |
| Fig. S4 F | MCF10A | IF, DNA, DEK | 13 | 3 | Qualitative |
| Fig. 5 is now Fig. 4 | | | | | |
| Fig. 4 A | U2-OS | | 56 | 2 | Quantitative; DEK body number in U2-OS |
| Fig. 6 is now Fig. 5 | | | | | |
| Fig. 5 A, B | U2-OS | IF, control | 136 | 13 | Quantitative; |
| Fig. 5 A, B | U2-OS | IF, CPT | 15 | 4 | Quantitative; |
| Fig. 5 A, B | U2-OS | IF, APH | 28 | 4 | Quantitative; |
| Fig. 7 is now Fig. 6 | | | | | |
| Fig. 6C | U2-OS | Screen | 500-750 cells per siRNA. For each siRNA 2-3 wells, 4 imaging positions/ well; 60-63 cells per position, cells per replicate. | 2 | Quantitative; |
| Fig. 8 is now Fig. 7 | | | | | |
| Fig. 7 F | U2-OS | WF, GFP-DEK/ GFP-SUMOmut | 1600, 2000 (80 positions per cell line, ~20-25 cells per position) | 2 | Quantitative; |

© 2023. Published by The Company of Biologists under the terms of the Creative Commons Attribution License (https://creativecommons.org/licenses/by/4.0/). 

| | | | | | |
|---|---|---|---|---|---|
| S Fig. 1 A, B, C | BJ5-TA | SIM, DEK bodies | 4 | 2 | Qualitative; |
| S Fig. 1 D | MCF7 | IF; EdU, DEK | 43 | 2 | Qualitative; |
| S. Fig. 1 E | MDA-MB-231 | IF; EdU, DEK | 46 | 2 | Qualitative; |
| | | | | | |
| S Fig. 2A | U2-OS KI eGFP-DEK | IF, PCNA, DEK | 280 | 2 | Qualitative |
| S Fig. 2B | U2-OS KI eGFP-DEK | IF, PCNA, DEK | 33 | 2 | Qualitative |
| S Fig. 2C | U2-OS | Transient expression of eGFP-DEK, RFP-PCNA, eGFP | eGFP-DEK: 23<br>RFP-PCNA: 24<br>Merge: 29<br>KI eGFP-DEK: 62 | 2 | Qualitative |
| S Fig. 2D | U2-OS | Transient expression of eGFP | 12 | 1 | Qualitative |
| | | | | | |
| S Fig. 3 A, B, C | MCF10A | See Fig. 3A, B | 93 | 5 | Quantitative |
| | | | | | |
| S Fig. 4A | U2-OS KI eGFP-DEK | IF, H3K9Me3, DEK | 300 | 2 | Qualitative |
| S Fig. 4B | U2-OS | IF, H3K9Me3, DEK | 150 | 2 | Qualitative |
| | | | | | |
| S Fig. 5G | MCF10A | eGFP-DEK, RFP-PCNA | 14 | 2 | Quantitative |
| | | | | | |
| S Fig. 7 E | U2-OS KI eGFP-DEK | Screen | SNEG: 60<br>sDEK: 220<br>sUBE2I: 200<br>sRPA1: 100 | | Quantitative |

1.5. Many graphs representing quantified data do not have statistical analyses to support the authors' conclusions, such as Figures 3B, 3G, 6B lower half, etc. A thorough application of statistical analyses is required. This may require increasing replicates (see point #4)

**Response**: For Figure 3G we performed a two-tailed t-test resulting in a p value of 0.009 using 20 cells from two independent experiments. For Figure 6B we included the statistical analysis, and we improved the section in the Materials and Methods. The analyzed data for Fig. 6B was based on the following samples: control - 87 cells in 7 replicates, CPT - 15 cells in 4 replicates, and APH - 28 cells in 4 replicates. The data has been analyzed by performing single-factor ANOVA. The statistical significance of the difference between control, APH-treated and CPT- treated cells was calculated using Student's t-test with a post-hoc test (Bonferroni correction) after assessing the variance (f-test). The statistical significance was set as 0.017 following the post- hoc test.

1.6. In the proximity ligation assay experiments in Figure 3C, there are abundant PLA signals that do not overlap with the DEK signal and the single control probe for H3K9me3 is not shown. This reviewer has low confidence that this assay was performed correctly due to what appears to be non-specific signal and missing controls. Adding the appropriate controls is essential for a revised manuscript.

**Response**: We fully agree with the reviewer that assessment of the quality of the PLA assays as originally shown can't be carried out to a satisfying level. The PLA assays presented in this manuscript have been performed during a larger experimental approach, which, of course, also entailed analyses of crucial control experiments and has been recently published (Pierzynska-Mach et al., 2023, Scientific Reports). Thus, we are confident that all appropriate and needed controls for PLA have been conducted to the best possible standards. Below we attach a panel presenting the performed PLA control experiments (Fig. R1). For each control specimen, one of the two targeted proteins was additionally immunostained in order to be confident that the PLA-marked structures have a proper spatial location within the sample. For samples staining within

© 2023. Published by The Company of Biologists under the terms of the Creative Commons Attribution License (https://creativecommons.org/licenses/by/4.0/). 

the cell nucleus, we checked the average PLA spot number, which is indicated below. We added the mentioned reference to the revised version of this manuscript.

NOTE: We have removed unpublished data that had been provided for the referees in confidence.

Moreover, we added in the text the explanation of the PLA spots outside of the DEK body (as it is known that DEK interacts with HP1 alpha and enhances its binding to H3K9me3, we hypothesize that the PLA spots outside of DEK body could show the proximity of such type). For better understanding of the results presented in the Figure 3 C and D, we performed a quantitative analysis of “PLA positive” DEK bodies, which we define as cases where PLA signal is formed in the radius of 1.5 µm from the center of DEK body, in case of Figure 3C in a couple between DEK and H3K27me3 and Figure 3D in a couple between DEK and H3K9me3. The chosen radius comes from the size of the entire PLA structure (primary antibodies, secondary antibodies, rolling circle amplification (RCA) product of several hundred nm in diameter. The results of this analysis show that for PLA between DEK in DEK bodies and H3K27me3 we did not identify any PLA positive DEK body (5 cells, 13 DEK bodies), while for DEK in DEK bodies and H3K9me3 we observed that 86% of DEK bodies was PLA-positive (measured in 26 cells, 46 DEK bodies).

**1.7.** The data for Figures 7 and 8 create conflicting data that the authors need to examine a little more closely. If this reviewer understood it correctly, the siRNA screen showed that the loss of the SUMO pathway INCREASED DEK bodies, but blocking DEK SUMOylation with mutations DECREASED DEK bodies. The authors very briefly include a potential hypothesis why this might happen (and it is a plausible hypothesis, that loss of SUMOylation will have a broader impact on other proteins than just DEK), but there are a few concerns to be considered here.

**Response**: We thank this reviewer, and also reviewers 2 and 3, for pointing out this obvious conundrum. We have made changes to several text sections and thank all three reviewers for their critical and very helpful comments.

a. First, the authors have not confirmed that the DNA binding ability of the DEK mutant is intact. Amino acids 144 and 145 are in the region between the SAP and pseudo-SAP domains that may impact the efficiency of DNA-binding in this region. Thus, the impact of this mutant on DEK bodies may be due to impaired DNA binding, not SUMOylation deficiency.

**Response**: We thank this reviewer for raising this critical point. In previous published and unpublished studies, we have tested the influence of a rather large set of amino acid substitutions in DEK on their impact on DNA binding affinity. Specifically, the K144A and K145A mutants have been tested in EMSAs and DNA supercoiling assays and showed no altered DNA binding affinity (Devany et.al. 2008). Mutations K61A, K62A reside outside of any known DNA binding domain, thus are expected to have minimal impact on DNA binding. K261A resides in the RNA binding domain of DEK, however, has not been identified as a target for nucleic acid interaction in our own, yet unpublished studies (unpublished work). As DEK-DNA binding appears to be rather complex, involving multiple points of interaction (Guo et al., 2021), we do not expect that the introduced mutational patterns affect general DNA binding to a great extent. However, as we can’t exclude that the introduced substitutions may affect conformational changes and thus potentially DNA binding of DEK at large, we performed two new experiments. EMSA with GST-tagged versions of DEK WT and the SUMOmut showed no substantial differences in DNA binding affinity between these two protein preparations (see Fig. R2 below, new Supplementary Figure 8A). This clearly indicates that the introduced amino acid substitutions do not affect DNA binding *in vitro*. However, and rather interestingly, in new cell fractionation studies with GFP-DEK WT and GFP- DEK SUMOmut (shown in response 2.40 to reviewer 2, new Supplementary Figure 8B) we found substantially altered extraction behavior. Interestingly, GFP-DEK-SUMOmut bound less firmly to chromatin and high molecular weight versions thereof are found in the nucleoplasm and even cytoplasm. This finding indicates that SUMOylation influences that modification pattern of DEK in cells and that this correlates with its capability of forming DEK bodies.

© 2023. Published by The Company of Biologists under the terms of the Creative Commons Attribution License (https://creativecommons.org/licenses/by/4.0/).

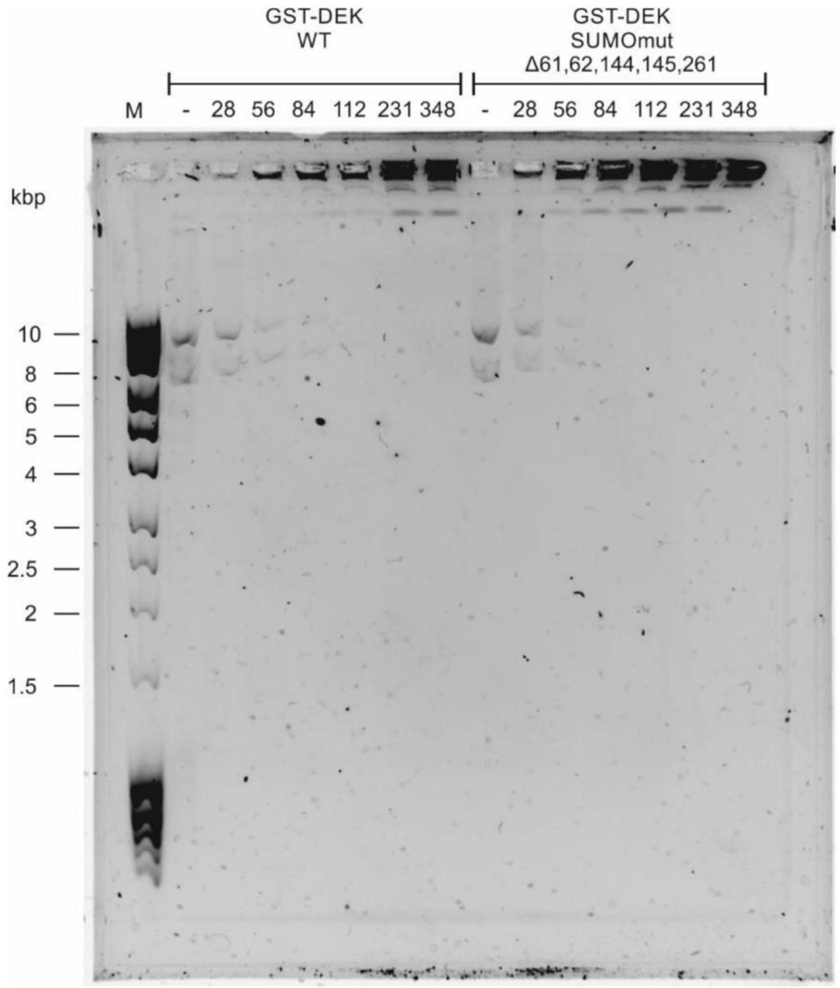


**Fig. R 2:** Electrophoretic Mobility Shift Assay (EMSA). Recombinant GST-tagged DEK protein (WT and SUMOmut) were incubated with plasmid DNA in increasing molar ratios as indicated. Agarose gel electrophoresis was performed to separate nucleoprotein complexes. For visualization the gel was incubated in GelRed nucleic acid stain and imaged with UV light. M: Marker.

**b.** Second, there still appear to be focal regions of increased signal in the DEK-SUMO mutant, they are larger but more diffuse. This reviewer recommends that the authors co-localize the GFP- DEK-SUMO mutant with PCNA or EdU as in previous figures. This will help determine if the mutant signal is still present but larger/more diffuse (potentially due to more DEK bodies, as would be expected with the siRNA data), or if the mutants truly prevent the formation of DEK bodies. This co-stain would also confirm that the images shown in the mutant are, indeed, from S phase cells. This experiment is considered essential by this reviewer for future revisions.

**Response:** As suggested, we performed co-immunofluorescence staining in U2-OS cells expressing either WT GFP=DEK or GFP-DEK-SUMOmut using PCNA and DEK-specific (K-877 Ab) antibodies. This indicated that independently of the GFP signal no endogenous DEK bodies form in cells expressing the SUMO-mutant in late S-phase. Shown below are maximum intensity projections of confocal Z-stacks. The nucleolar-like signal that appears in the WT cells may have its origin in the methanol fixation/permeabilization required for staining with the PCNA antibody (Zarębski M Bosire R Wesołowska J Szelest O Eatmann A Jasińska-Konior K Kepp O Kroemer G Szabo G Dobrucki JW (2021) Translocation of chromatin proteins to nucleoli-the influence of protein dynamics on post-fixation localization Cytometry. Part A 99:1230–1239.)

© 2023. Published by The Company of Biologists under the terms of the Creative Commons Attribution License (https://creativecommons.org/licenses/by/4.0/).

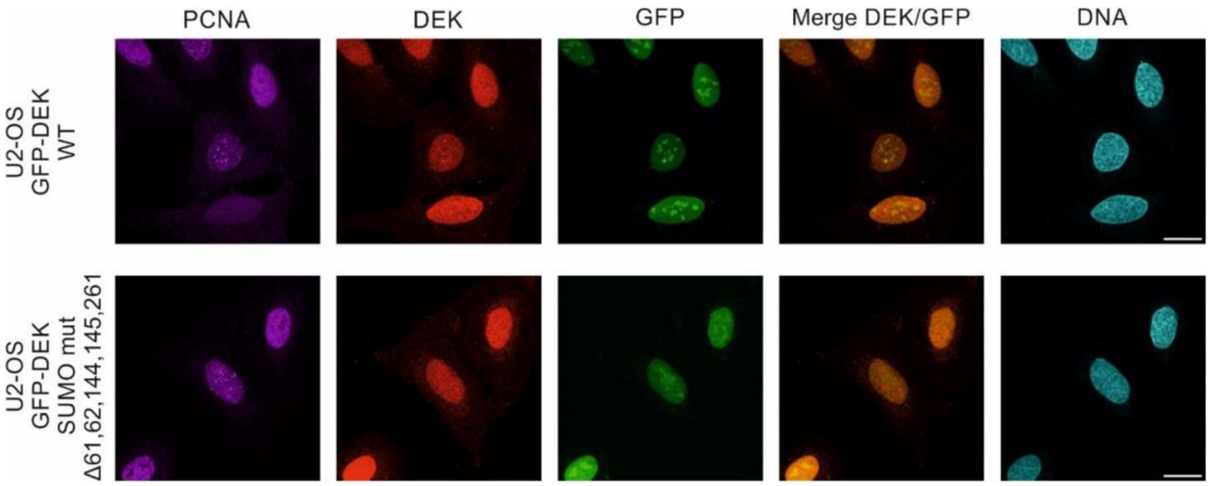


**Fig. R3**: Maximum intensity projections of confocal Z-stacks of U2-OS GFP-DEK WT cells (upper row) and U2-OS GFP-DEK SUMOmut cells (bottom row). Endogenous DEK (red, K-877 Ab) and PCNA (magenta) were visualized by specific antibodies. GFP-tagged DEK is shown in green and DNA was counterstained with Hoechst33342 (cyan). Scale bar: 20 µm.

The microscopy images in 7F originate from timelapse microscopy data where cells were imaged every 12 minutes for 22 hours. The figure below shows an extract of this dataset. The U2-OS mutant shows normal cell division as seen in the WT except for DEK body formation. Also, the cell density increases in both cell lines to the same extent over time showing comparable cell doubling times. These files are now attached as Supplemental Movies 1 and 2.

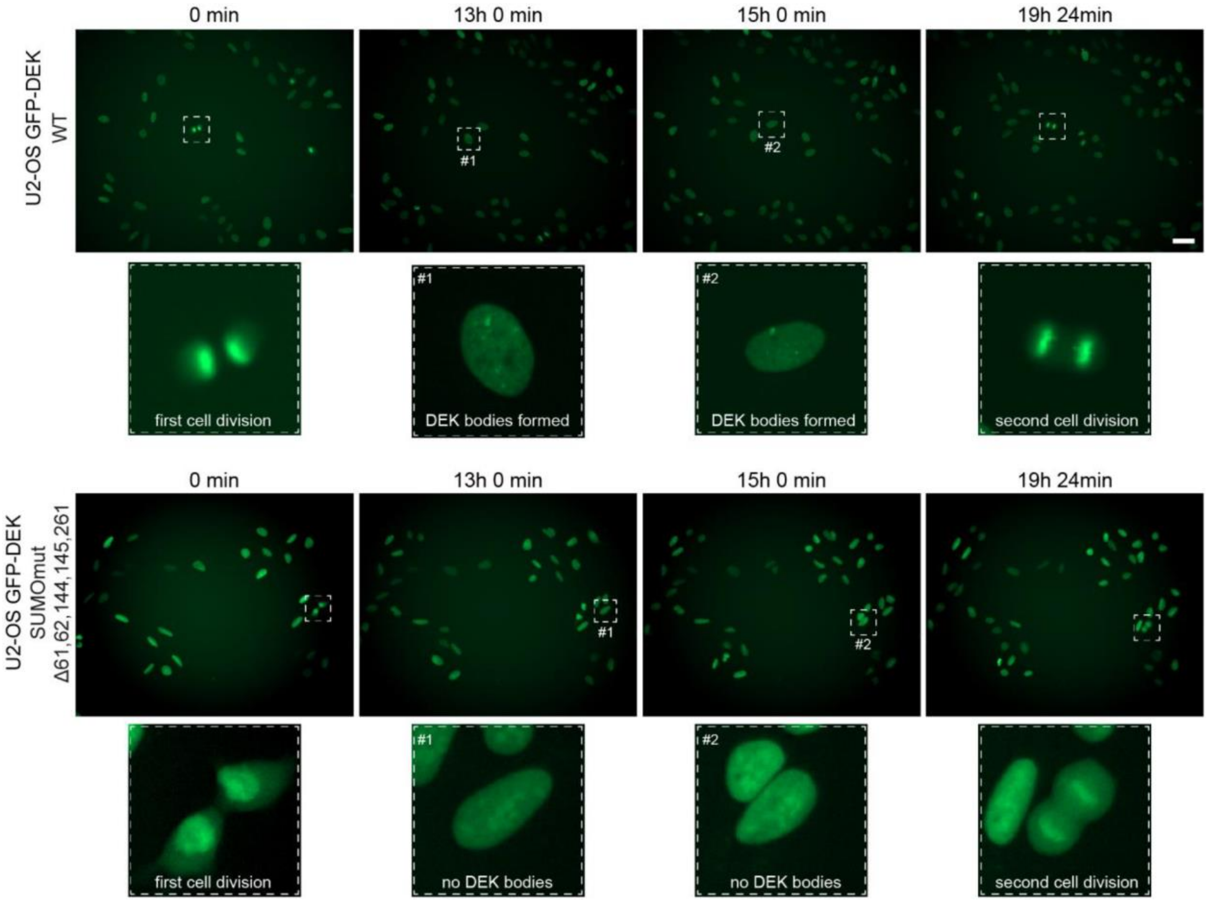


**Fig. R4**: Representative still images of timelapse widefield microscopy movies of U2-OS DEK WT and SUMOmut cell lines. One scene per cell line was chosen and four frames were extracted. The

© 2023. Published by The Company of Biologists under the terms of the Creative Commons Attribution License (https://creativecommons.org/licenses/by/4.0/).

first frames are at pseudo-time 0 min corresponding to the anaphases of the selected cells (marked by dashed boxes) After 13 or 15 hours DEK body formation in daughter cells (#1, #2) is only observed in the DEK WT but not in the SUMOmut cell line. Subsequently, and within similar timeframes, both cells expressing WT or the SUMOmut undergo the next cell division.

c. Third, using a small molecular inhibitor of SUMOylation (i.e.: TAK-981) will better support the siRNA screen that suggests SUMOylation inhibition impacts DEK body formation. Since the DEK mutant and SUMO pathway knockdown data are conflicting, adding an additional method, like TAK-981, may help resolve the discrepancy.

**Response**: We fully agree with this reviewer. However, we are currently not in the position to conduct inhibitor experiments and thus can't provide additional data at this point. As we are aware of contradictions in respect to the role of SUMO in DEK body formation, we made several changes to the results and discussion. Such proposed inhibitor studies will be certainly conducted in future experiments.

**Minor Comments (not related to experiments)**

**1.8.** In the introduction, the authors mention a few times that "DEK is highly enriched at maturing chromatin" but no references are included to support this statement.

**Response**: The references have been included on page 3 of the manuscript as shown below:
"We and others have identified the DEK oncogene, a unique and multifunctional non- histone chromosomal protein, as a supportive factor of DNA replication, particularly in scenarios where the replication machinery is under stress (Deutzmann et al., 2015; Ganz et al., 2019). Although Isolation of Proteins on Nascent DNA (iPOND) studies support no direct association of DEK with the replisome, DEK is enriched in maturing chromatin (Alabert et al., 2014; Aranda et al., 2014; Garcia et al., 2017; Lossaint et al., 2013; Ribeyre et al., 2016; Sirbu et al., 2013)"

**1.9.** In the "DEK bodies are novel players…" section of the Discussion, the authors state "In this study, over-expression of KAT2B [histone acetyltransferase] led to an accumulation of DEK in interchromatin granule clusters (IGCs)…" but data from KAT2B experiments are NOT included in this manuscript draft. Please either add the data or remove this statement.

**Response**: We thank this reviewer for this comment. As reviewers 2 and 3 also highlighted this point, we amended the results and discussion section accordingly and mention the large array of DEK body regulators in the results section.

**1.10**. In Figure 4, the authors mention that DEK bodies can be in close proximity to the nuclear envelope and nuclear invaginations. The relevance of this observation needs to be described.

**Response**: We thank this reviewer for this comment. As the entire original Figure 4 is based on qualitative data sets, we decided to move this Figure to the supplements (now Fig. S5). However, this does not change the fact that data shown in this figure provide additional support for DEK bodies being predominantly located in heterochromatic areas. Please see also response to point 2.26 for reviewer 2 for more explanations.

***********************************************************************************************************

## Reviewer 2

The authors identified an accumulation of the oncoprotein DEK at late DNA replication sites, which correspond to constitutive heterochromatin. How DNA embedded in the complex structure of heterochromatin is replicated is a very active field of research, as it is also associated to the epigenetic memory of these regions. Finding a new factor potentially involved in the replication of heterochromatin is, therefore, of interest for the field, especially since its accumulation at late replicating sites seems to be modulated by SUMO, a protein already found to accumulate at these sites in other systems. The findings from this study could definitely participate to a better understanding of the mechanisms underlying the replication of heterochromatin and give new directions for investigating this process.

© 2023. Published by The Company of Biologists under the terms of the Creative Commons Attribution License (https://creativecommons.org/licenses/by/4.0/). 

**Response:** We would like to thank this reviewer for the highly positive comments on our work and the extremely thorough and very helpful suggestions which we addressed below.

**Comments for the Author:**

I found the article very interesting and finding DEK at heterochromatin replication sites could open new ways to investigate the mechanisms underlying the replication of DNA in the complex structures of dense heterochromatic regions. However, before publication, several points should be addressed and/or clarified, in particular regarding the role of SUMOylation in the formation of the DEK bodies (last part, see specific points below).

2.1 Also, since SUMO is an important factor for the formation of PML bodies and since PML bodies were also found at late replication sites in other systems, the authors should definitely look at the overlap between the DEK and the PML bodies.

**Response:** We thank this reviewer for this comment. We have very early on tested for potential co-localization of DEK bodies with PML, given the known connection. However, co-immunostaining of DEK and PML in U2-OS cells did not provide indications of close proximity. We attached two representative examples of these experiments below.

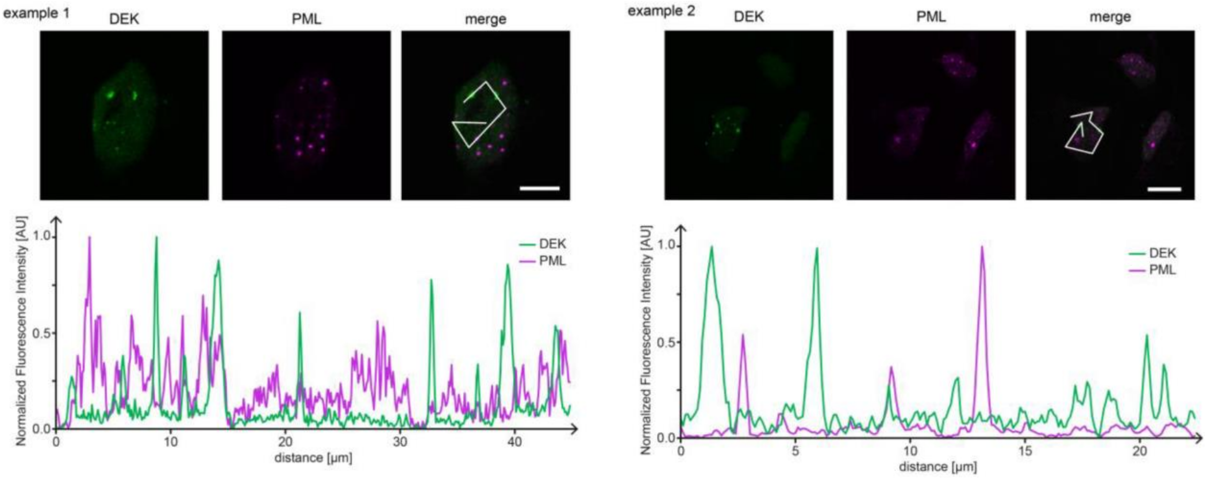


**Fig. R5:** Upper panels: Confocal images of U2-OS KI eGFP-DEK cells labelled with PML-specific antibody (mouse monoclonal α-PML, Santa Cruz E-11) (magenta). The GFP-DEK signal is displayed in green. Scale bar: 10 µm. Bottom panels: Fluorescence intensity profiles for the analysis of PML (magenta) and DEK (green) colocalization. The white line displayed in the merge panel is given as distance in the graph.

2.2 I enjoyed reading the imaging part of the manuscript. However, the authors use different types of super-resolution microscopy (STED, dSTORM, 3D-SIM) but why they chose some and not others depending on the context is not discussed at all. It may be obvious for microscopists, but this should be briefly explained, at least in the Material and Methods section.

**Response:** We would like to thank this reviewer for this comment. As this is the first report describing DEK bodies, we were eager to capture as many structural details as possible. Therefore, we employed the full range of super-resolution microscopy approaches available to our laboratories. These techniques are confirmatory yet also complementary to each other and allow a deeper insight into the ultrastructure of DEK bodies. Even though the ultra-structure of DEK bodies is not the major focus of this work, we think that the presented super-resolution approaches provide useful information for further studies and for specialists in the field. We added additional information to the methods section and rephrased the relevant sentence in the manuscript on page 7. See also response to point 3.4 for reviewer 3.

2.3 I am still unsure after reading the manuscript what the DEK bodies do contain, in particular regarding PCNA and H3K9me3. To me, it looks like that they form a heterogenous population, but this is almost not discussed by the authors. And this information is missing since experiments such as FRAP were done at the single body level (see below for more details).

© 2023. Published by The Company of Biologists under the terms of the Creative Commons Attribution License (https://creativecommons.org/licenses/by/4.0/).

**Response**: We thank the reviewer for raising this important point and we would like to express our gratitude for the patience of this reviewer. Prompted also by comments from reviewers 1 and 3, we have extended our data analyses for most experiments. These are presented in response to points 1.4, 1.5 and 1.6 to reviewer 1. Thus, we are now confident that DEK bodies are formed predominantly in regions containing H3K9me3. In addition, we analyzed co-occurrence of DEK with PCNA and EDU and vice versa as requested in point 2.11. Thus, we hope that these additional data analyses will help in painting a clearer picture of the nature of DEK bodies.

**2.4** There is not a single statistical analysis, which is unfortunate as we can not assess the robustness of the analyses and the significance of the differences. This should be done at several places. I also think that the way some results are presented is too "shallow" and the reader has to find the information within the figures and the rational by themselves. Some results are actually discussed in the discussion without being presented in the results section. I will be glad to review a revised manuscript.

**Response**: We thank the reviewer for pointing out these weaknesses in the first version. We have now followed suit and made changes to the following sections:

- Statistical analysis related to the colocalization of DEK bodies with EdU regions (DEK → EdU and EdU → DEK) has been performed.
- Statistical analysis related to the colocalization of the DEK bodies and PCNA foci (DEK → PCNA and PCNA → DEK) has been performed.
- Statistical analysis related to DEK and post-translational histone modifications regions (Fig. 3) has been performed.
- FRAP experiments (statistical significance of the difference) (Fig. 3)
- Statistical analysis (ANOVA, Student's t-test with Bonferroni correction) for Figure 6B has been performed.

In addition, we systematically listed cell numbers and replicates in the table in point 1.4 in response to reviewer 1.

**Specific points**

**Results**

**A sub-population of DEK molecules assembles into complex nuclear bodies at sites of late DNA replication**

**2.5** Page 7 "A sub-population of DEK molecules assembles into complex nuclear bodies...". Why complex?

**Response**: Our goal was to highlight to the community that DEK bodies seemingly have an intricate and complex internal structure (see also 2.2 and point 3.4), which was based on the super-resolution approaches. As we currently can't provide more detailed analyses regarding the ultra-structure of DEK bodies, our intention in the manuscript was to state this fact using the umbrella term "complex". However, we now removed the term complex from the heading and mention "intricate internal structure" within the same paragraph.

**2.6** Legend of Figure 1A " Average number of DEK foci per cell". In which cells? (late S phase I would guess?) Also, "Wildfield images" is indicated in the legend for panel A and D, but not B and C. In my opinion, "Widefield images of MCF10A cells" should not only be for panel A, but also for panels B and C (A-C).

**Response**: We thank this reviewer for pointing this out. DEK bodies in cells were counted based on EdU staining patterns and thus the number represented cells in S-phase. This has been added to the legend. The label "widefield" has been placed erroneously and has been changed to confocal as only confocal images are shown.

**2.7** Figure 1B and 1C. Figure 1C shows the distribution of PCNA and EdU in addition to DEK in MCF10A cells, even though not mentioned in the legend (in the text, it is mentioned either one or

© 2023. Published by The Company of Biologists under the terms of the Creative Commons Attribution License (https://creativecommons.org/licenses/by/4.0/).

the other). Why then having a separate panel for EdU alone (1B)? Couldn't the authors show the fluorescence intensity profile corresponding to EdU along those corresponding to PCNA and DEK, and then get rid of panel B? Alternatively, EdU should not be shown in panel C.

Response: We included the EdU channel in Figure 1C (now 1B) and depicted the corresponding fluorescence line profile as suggested.

2.8 Legend of Supplementary Figure S1. The average number of DEK foci per cell should be indicated for each cell type, as in Figure 1.

Response: We thank the reviewer for this comment. We added the average number for DEK bodies in BJ5-TA cells, which is 2.25 (4 cells from two independent experiments). However, it must be noted that this is qualitative data and the data set only includes a small number of cells. Also, as MCF7 and MDA-MB-231 cells do not form DEK foci, such an analysis couldn't be conducted. We also modified the figure legend accordingly.

2.9 Legend of Figure 2A-B. For 2A, the legend says "The STED image reveals a granular internal structure of DEK bodies (insets)". What does the "magnified image of the ROI shown in the middle containing a DEK body" show in 2B? Did the use of two types of super-resolution microscopy allow the authors to reveal different structures, or do they simply support each other?

Response: We added dashed lines to show that the highlighted ROI in Fig. 2B middle panel is magnified in the right panel. Here, we wished to show the internal structure of DEK bodies by using complementary super-resolution microscopy techniques (see also 2.2).

2.10 Page 8 " [...] followed by 3D Structured Illumination Microscopy (3D-SIM)". Not clear how this was done in the Material and Methods section (as opposed to STORM).

Response: We apologize for having omitted crucial information regarding 3D-SIM in the first submission. We have now added the required information to the methods section and to the legend. See also additional explanation under response to point 1.3 for reviewer 1 above.

**DEK bodies assemble at replicating heterochromatic regions**

2.11 Page 8 "The occurrence of DEK in a subpopulation of PCNA- and EdU-positive nuclear bodies in late S-phase". This is an interesting observation. Not all EdU/PCNA foci (nuclear bodies) are found enriched in DEK (according to Fig.1A-C). How about the opposite? Do all DEK foci "contain" PCNA and/or EdU? When looking at Fig.1B, it does not seem so. Could the authors comment on that and maybe provide a quantitative analysis indicating how many DEK foci (% ?) are associated with the two other types of foci? And vice versa? Since they calculated the average number of DEK foci per cell (6.7 ± 2.2, n=46 cells, Fig.1), this should be easily doable.

Response: We increased the sample size in order to provide more accurate data analysis of the EdU regions or PCNA foci association with DEK bodies, and vice versa. We found that 90% ± 18 of DEK bodies associate with EdU regions and 17% ± 8 of EDU regions associate with DEK. This analysis was done on 38 DEK bodies and 218 EDU regions from two independent experiments. A similar pattern was observed in co-localization between DEK bodies and PCNA foci. 78% ± 15 of DEK bodies associate with PCNA foci, whereas 11% ± 4 of PCNA foci associate with DEK. Analysis was carried out with 37 DEK bodies and 254 PCNA foci from 7 cells performed in two independent experiments.

Although in both cases (DEK bodies associating with EdU-marked regions and DEK bodies associating with PCNA foci) the fraction of DEK bodies positively associating with these structures is very high, we observe higher percentage in the case of DEK and EdU. We associate this result with the technical aspect of the preparation of samples, which requires a 20-minutes incubation with the EdU compound. Therefore, the EdU regions' fluorescence signal represents the DNA fragments which underwent DNA replication during the preceding 20 min, before cells fixation. On the other hand, a slightly lower DEK-PCNA association fraction could be explained by PCNA presence in other, not DNA replication-related, nuclear processes, such as DNA repair.

© 2023. Published by The Company of Biologists under the terms of the Creative Commons Attribution License (https://creativecommons.org/licenses/by/4.0/).

2.12 Figure 3A. The intensity profiles of EdU should be added to each graph (as in Figure 4A), to show that the authors are looking at replicating chromatin and to support their conclusion. It is actually true for all graph showing intensity profiles in late S phase.

**Response**: We added the line profiles of the EdU fluorescence signal in all the panels related to panels depicting late S-phase. This included Fig. 1B, Fig. 3A, Fig. 4A, Fig. 4C, and Fig. 4F (Fig. 4 is now Supplementary Figure S5).

For former Fig. 4B, we decided not to add the EdU line profile as this panel presents a cell nucleus in a non-replicative phase. Due to the fact of the normalization of the fluorescence signal to min/max, this representation would be misleading for the reader (absence of incorporated EdU, noise pixels detected with max values of about 2 A.U., where the values of DEK and CENP-A are around 240-250 A.U.).

2.13 Page 8 "This conclusion was corroborated by Manders' coefficient analysis". I am not sure to understand how this analysis was performed. What does n represent? (n=8 from two independent experiments). This number (8) seems rather small (the upper whisker for H3K9ac goes above the one for H3K27me3). Which DEK foci were analyzed? From what I can see on the figures, not all foci contain H3K27me3 or H3K9me3. This is not clear and should be better explained.

**Response**: We thank the reviewer for pointing that out. By "n" we mean the number of nuclei analyzed for each pair of proteins which was accordingly n(DEK&H3K9ac) = 8, n(DEK&H3K27me3) = 8, n(DEK&H3K9me3) = 5. The number of foci analyzed within each couple was: n foci = 29, 37 and 35 respectively. We added a more detailed description of the Manders' overlap coefficient calculation in the Materials and Methods section.

In order to construct the Figure 3 B, the colocalization fraction was calculated using JaCoP Fiji Plugin. This image analysis is based on the Pearson's correlation coefficient. The depicted value on the graph "M1 Manders' coefficient" is defined as the ratio of the summed intensities of pixels from microscopy acquisition channel 1 (in our case, DEK bodies signal) for which the intensity in the second channel (histone modifications) is above zero, to the total intensity in the first channel. In other words, it is a reliable indicator of the proportion of the first channel signal coincident with the signal in the second channel over its total intensity. The Manders' coefficient varies from 0 to 1 which corresponds to non-overlapping images and 100% co-localization between both images.

What we performed in the case of this analysis of the association of the DEK bodies to the H3K9ac, H3K27me3 and H3K9me3 signal, was the measurement of the total DEK bodies signal within a single cell nucleus. Therefore, we applied a fluorescence intensity-based mask, in order to separate DEK bodies from other DEK nuclear signal, and we performed the image analysis.

2.14 Page 8, FRAP experiment. While I understand how the acquisitions were made and how the mean fluorescence intensity within the bleached region in each case was monitored, I have a problem understanding how the normalization was done. The authors write in their methods (page 23) "the curves were combined and normalized to the minimum and maximum of the combined curve". What is the combined curve? Shouldn't each curve be normalized according to their own maximum/minimum? (so the maximum is always 1 and, in that case, the minimum 0). Actually, the authors write page 22 "all traces were min/max normalized, averaged and the standard error was calculated.", which is what I would expect from such an analysis. I am confused about what was done. Also, what does "Finally, each of the curves was fit" mean (Page 23)? Also, according to the different images provided by the authors showing both DEK (either endogenous or overexpressed) and H3K9me3 distribution patterns, not all DEK bodies seem to contain H3K9me3. So how were the bleached foci selected? The legend for Figure 3F says "Data points show the average of 6 experiments for DEK bodies [...]". Does that mean that 6 FRAP experiments were done? Or one experiment with 6 cells cells? Were the bleached foci from different nuclei? If, as I mentioned previously, DEK bodies are not systematically enriched in H3K9me3, how to be sure that the bodies the authors looked at by FRAP are really associated with heterochromatin? Information should be provided about the occurrence of H3K9me3 and DEK-EGFP foci colocalization in MCF10A when DEK-EGFP is transiently expressed (only images in U2-OS cells overexpressing eGFP-DEK is provided in Supplemental Figure S4). If several types of DEK bodies (with and without H3K9me3)

© 2023. Published by The Company of Biologists under the terms of the Creative Commons Attribution License (https://creativecommons.org/licenses/by/4.0/).

do co-exist within a given nucleus, then "6" is not enough to conclude anything. The authors could bleach several (or all) DEK foci from the same nucleus and verify if they all show the same type of recovery curve (or not). If all eGFP-DEK bodies in transfected MCF10A do contain H3K9me3, then this should be clearly shown. Finally, the eGFP- DEK foci (focus) bleached in Figure 3E is large, close to the nuclear periphery, and resembles to the one associated with H3K27me3 in Figure 3A. Is it representative of all DEK bodies? For all the aforementioned reasons, I find it difficult to draw a conclusion from the results of this experiment.

**Response**: We thank the reviewer for raising this point. We would firstly like to clarify that FRAP was only applied to test for potential variations in molecular mobility of DEK within DEK bodies as compared to DEK molecules found elsewhere. As such, FRAP was not intended as a tool to claim that DEK bodies are found in heterochromatin. We amended the corresponding section in the manuscript to accurately reflect our intentions. The choice of DEK bodies which were subjected to FRAP experiments has been made based on their position and size. In order to perform FRAP correctly, we have chosen bigger foci which could be photobleached in their entirety by the high intensity laser power within a ROI of 2 µm x 2 µm. All DEK bodies assessed were positioned at the periphery of the cell nucleus or close to the nucleolus where heterochromatin typically is located. However, this is not sufficient to claim a clear association with heterochromatin, as the reviewer rightfully pointed out. At the time these experiments were performed, we had knowledge that DEK bodies are related to DNA replication of heterochromatin. Thus, we employed FRAP to obtain additional information about DEK body internal dynamics.

In order to answer the Reviewer's question, we attach the six examples of DEK bodies (marked with an arrow) on which the FRAP experiment has been performed. We also rephrased the corresponding sections in the methods section:

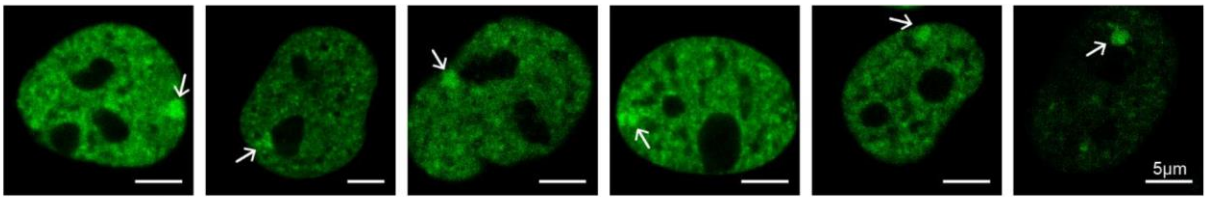


**Fig. R 6.** Overview of the six DEK bodies used in FRAP experiments for Fig. 3E, F and G.

**2.15** Page 8, "In line with these data, DEK bodies were associated with heterochromatin, in particular with H3K9me3, also in U2-OS wild type and U2-OS KI eGFP-DEK cells (Fig. S4)." As mentioned above, not all DEK bodies seem to contain H3K9me3 (hence my comments on the FRAP experiment). The line drawn in panel C exclude one foci (focus) that most likely does not contain H3K9me3. The authors should be more precise when presenting their results and mention whether all DEK bodies contain H3K9me3 or not.

**Response**: We thank the reviewer for this comment. We re-analyzed the respective figure (Fig. S4) and included the originally missing DEK focus in the line analysis. Thus, we conclude that the majority of DEK foci contain H3K9me3. We also stated this fact by adding "predominantly" to the respective sentence.

**2.16** Page 9, "Maximum intensity projections of confocal Z-stacks revealed that the epigenetic mark for centromeres, the histone H3 variant CENP-A, reproducibly decorates DEK bodies from both sides (Fig. 4A)". How were the top and bottom of the Z-stacks defined? Do they correspond to the top and bottom of the DEK bodies only, or are fluorescent signals above and under these bodies also captured? The reason I am asking this is because I do not understand how/why in 3D, CENP-A signals would flank the DEK-bodies only along the x/y axes. This would be easily explained if the z-stacks only covered in z the volume of the DEK bodies and ignored what is above and under. However, if the stacks also contain signals located above and under the bodies, then why do we not see CENP-A in the middle of some bodies, since we look at max projections along the z-axis? This should be clarified either here or in the methods section. This is also the case when analyzing the staining of the Barr body in regards to the DEK bodies (Figure 4C). And again, are all DEK bodies decorated by CENP-A?

© 2023. Published by The Company of Biologists under the terms of the Creative Commons Attribution License (https://creativecommons.org/licenses/by/4.0/). 

**Response:** We thank this reviewer for the comments on Figure 4 (see also questions 2.17; 2.18; 2.19; 2.20), which we would like to firstly address with a general response:

Throughout this study we observed the spatial proximity of DEK bodies and typical heterochromatin compartments. The former figure 4 (now supplementary figure S5) depicts qualitative observations of such phenomena and includes markers of typical heterochromatin compartments such as: histone H3 variant (CENP-A), typical centromeres' marker, *Xist* RNA stained using FISH-RNA which reveals the inactive heterochromatin site of inactivated chromosome X, chromatin region in proximity to nuclear envelope (immunolabelled against lamin-A) which represent a general heterochromatin compartments of perinuclear location or close to so called nuclear invaginations, and lastly heterochromatin stained with a DNA dye localized by a dense fluorescence signal with higher signal intensity. We show these results as qualitative data which, in our opinion, is very promising for further analysis and could be a subject of future research including distance-based image analysis coming from z-stack confocal acquisition, cell cycle analysis or after induction of chromatin-relaxation inducing agents. However, as most data sets in Fig.4 are of qualitative nature, we now present this figure as Supplementary Fig. S5.

The Z-stack for each cell contains the whole volume of the cell nucleus. For each case the total height in Z axis is about 3 µm imaged in about 14-15 planes (with the distance in Z of about 0.21 µm). In the manuscript we show maximum projection of a representative cell in which it is possible to observe some of the DEK bodies being decorated with CENP-A. In order to satisfy the reviewer's curiosity, we attach below other 2D confocal imaging examples of similar cases.

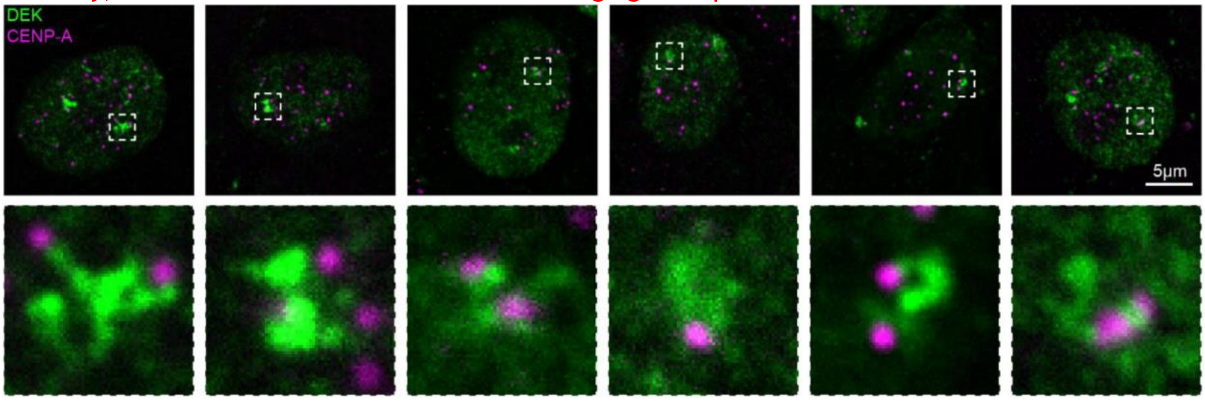


**Fig. R 7:** Examples of the spatial proximity of DEK bodies with immunolabelled CENP-A protein in MCF10A cells.

**2.17** Figure 4A. Four ROI are drawn, why? One has an asterisk, which is the one shown under I think. But no indication in the legend.

**Response:** We modified Figure S4A, and we left only one ROI, which is shown also as an inset.

**2.18** Page 9, "We did not observe spatial proximity of DEK and CENP-A outside of late S (Fig. 4B)". In Figure 4B, the authors selected one CENP-A foci (focus) which does not overlap with DEK. However, I can see at least two white foci indicating a colocalization, at this level of resolution, between DEK and CENP-A. Can the authors comment on that?

**Response:** We thank the reviewer for pointing this out. In total we analyzed 29 cells from two biological replicates. From these examples, we conclude that there is predominant spatial proximity between DEK bodies and CENP-A *only* in late S-phase, when DEK bodies occur. However, cells in non-S-phase show predominantly no co-localization between DEK and CENP-A. This is in agreement with studies of Ivanauskiene et al. 2014 (Supp Fig 1B in their publication), also showing no co-localization of DEK and CENP-A in human MSC cells. We have also attached additional experiments showing co-staining of DEK and CENP-A in non-S-phase cells.

© 2023. Published by The Company of Biologists under the terms of the Creative Commons Attribution License (https://creativecommons.org/licenses/by/4.0/).

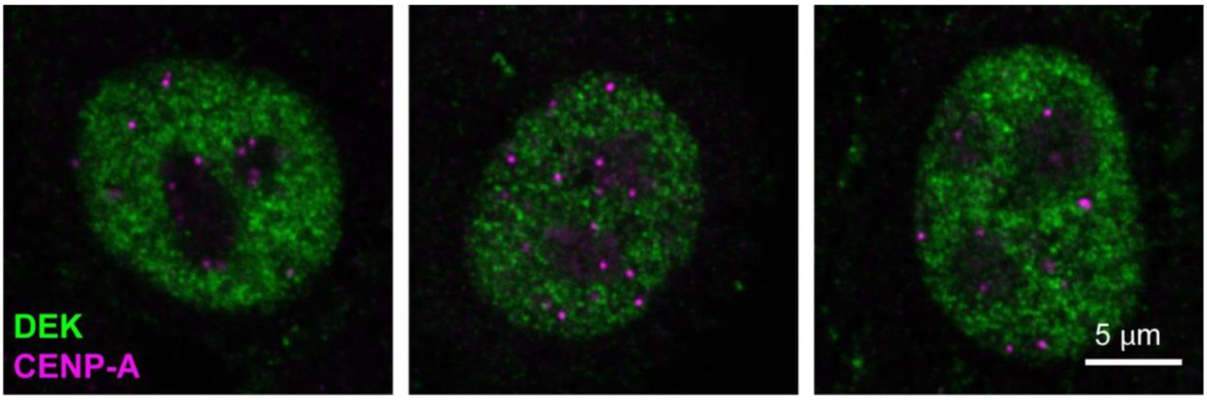


**Figure R 8**: Examples of immunolabelled CENP-A and DEK proteins confocal images of MCF10A cell nuclei in non-S phase.

2.19 Page 9, "Similarly, we found DEK bodies in close proximity of the nuclear periphery and Lamin-A positive nuclear invaginations only in S-phase (Fig. 4D, E)". When a nuclear lamina invagination (lamin A staining) is observed, is DEK systematically present within close proximity? The number of invaginations usually differs from nucleus to nucleus.

**Response**: In all cases we observed this was the case. However due to the rareness of the endogenous nuclear lamina invaginations and our limited sample size we do not provide statistical analysis on that, and rather present a qualitative observation. See also point 2.16.

2.20 Page 9, " DEK bodies corresponded to regions of dense chromatin, as shown by DNA staining with ToPro3 (Fig. 4F)". In the magnified image, why did the authors draw a line "along the white area" where the two signals (DNA and DEK) overlap, and not along the perpendicular direction, as they did for H3K27me3 to show a juxtaposition instead of a colocalization? This should be fixed or explained.

**Response**: In Figure 4F (now Supplementary Fig. S5F) we show the position of DEK bodies in relation to dense regions of chromatin. Considering that DEK bodies associate to a specific type of heterochromatin (as shown on the Figure 1 and Figure 3 A – therefore replicating constitutive heterochromatin) we did not obtain the full colocalization of DEK and ToPro3-stained DNA region. However, depicted DEK body localizes within the DNA-dense region, of which, we assume, a part consists of constitutive heterochromatin. We include below the fluorescence line profile suggested by the reviewer. Although the DEK body signal and dense DNA signal maximums do not overlap, the DEK body is within the more DNA-dense region, which we show in the light grey zone on the plot. For the manuscript, we have chosen the other line profile, that, we believe, will be more comprehensive for the reader.

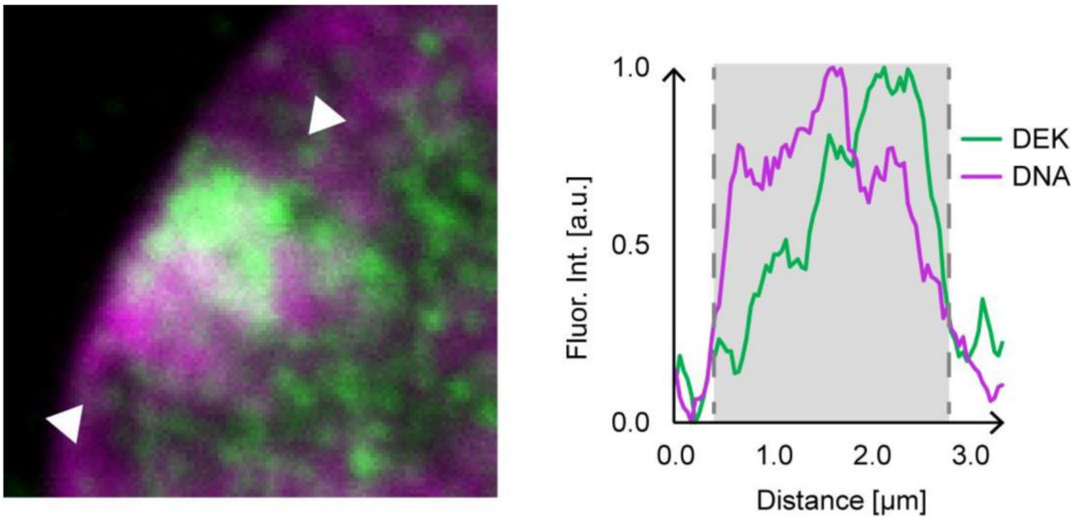


**Figure R 9:** Confocal image of a dense-DNA region and DEK body position in MCF10A cell nucleus in

© 2023. Published by The Company of Biologists under the terms of the Creative Commons Attribution License (https://creativecommons.org/licenses/by/4.0/).

late S-phase labeled with DEK-specific antibodies and with the DNA dye ToPRo3. The fluorescence intensity profile was calculated along the lines delimited by the white arrowheads and normalized between 0 and 1. The grey area on the plot indicates the zone of DEK body along the plotted line profile.

2.21 Page 9, "Altogether, our collective microscopy data strongly support a role for DEK bodies in the replication of heterochromatic chromatin regions in late S-phase." I agree, even though I think more precision should be added in some parts of the results.

**Response**: We thank the reviewer for this comment. We do agree that the conclusions are supported by Figures 1-3. However, as former Fig. 4 would require very complex additional studies to allow for quantitative data analysis, we present the entire Fig. 4 now as Supplementary Fig. S5. However, this change in presentation does not diminish the value of the presented experiments.

**DEK body dynamics is affected by DNA replication inhibitors**

2.22 Page 9, " time-lapse imaging experiments at a spinning disk confocal microscope". Should be"with" or "using".

**Response**: Thank you for pointing out this typo, which has now been fixed.

2.23 Page 9, " 2 ± 18% of DEK bodies colocalized with PCNA-marked replication foci in these cells (object-based analysis of 19 DEK bodies)". Why was the JaCoP plugin used here while the number of DEK bodies was assessed manually in U2-OS cells? Also, why only 19 bodies analyzed, which correspond to 3 cells, according to "6.7±2,2 bodies per nucleus" (out to 13, according to the supplementary figure S5's legend).

**Response**: We increased the sample size in the DEK bodies – PCNA analysis (see 2.11 above). We assessed 37 DEK bodies and 254 PCNA foci in 7 cells from two independent experiments, which resulted with the 78% ± 15% colocalization fraction from object-based analysis JaCoP plugin approach. The difference in techniques applied results from the fact that the analyses were performed in different labs with own established procedures. The analysis of DEK bodies and PCNA was performed based on 2D confocal imaging, while the number of DEK bodies in MCF10A cells was obtained from 3D confocal z-stack projection.

2.24 Page 10, "Of the three substances, aphidicolin showed the most prominent effect reducing DEK body number by 47%, and prolonging DEK body lifetime by 28%." The number of foci being relatively low in the control, the authors should give numbers for the different conditions and compare them instead of indicating by which percentage they differ. Also, a statistical analysis should be performed in figure 6B (and at several other places) to address the significance of the observed differences. Between 15 and 132 cells were analyzed, which is a very broad window. The exact number for each condition should be indicated somewhere (in figure, legend or methods).

**Response**: We thank this reviewer for commenting on this issue. As outlined in the response to reviewer 1 (point 1.4 and 1.5) we now provide more detailed statistical analysis between the conditions.

2.25 Page 10, " Camptothecin had a similar but more moderate effect, and hydroxyurea did not alter DEK body dynamics". The experiment was done once and HU did not seem to have much effect on the duration of G2, as opposed to the other inhibitors. How can the authors be sure that they used HU in the proper condition? I think that the data provided here are not sufficient for the author to conclude that their results "strongly suggests that DEK bodies are implicated in the replication of DNA structures particularly susceptible to aphidicolin". The significance of the differences is important to assess, as APH and CPT are further used in the positive controls for the siRNA screen.

**Response**: We thank the reviewer for this comment. We have removed data relating to HU treatment from the text and figure.

**Down-regulation of the SUMO pathway increases DEK body number in an siRNA screen for DEK**

© 2023. Published by The Company of Biologists under the terms of the Creative Commons Attribution License (https://creativecommons.org/licenses/by/4.0/).

**body regulators**

2.26 Supplementay Matetrial and Methods, Page 27. What is COC? Where are the COC imaging from? Supplementay Matetrial and Methods, Page 29. [...] these track identifiers were used to summarize relevant measurements such as DEK body lifetime, number of DEK bodies per track etc." The DEK body lifetimes and number of DEK bodies were previously used by the authors. What were the other relevant parameters? Only the cited ones are shown in Supplemental Figure S6.

Response: We thank this reviewer for this comment. COC stands for cyclic olefin copolymer (COC) which was used to coat the plates to allow for imaging plates (Greiner). This is part of an established solid-phase lipid-based procedure developed at the EMBL by (Neumann et al., 2006): Briefly, imaging plates or microarrays are coated with a mixture of siRNA, medium, sucrose, gelatin and transfection reagent (Lipofectamine 2000, Thermo Fisher). The transfection mix is then lyophilized and plates can be stored in plastic boxes. Upon seeding of cultured cells, the dried transfection complexes resolve and are competent for siRNA delivery. For the screening assays, 96-well cyclic olefin copolymer (COC) imaging plates (Greiner) and locked nucleic acids (LNA)-modified 21-bp duplex siRNAs with overhang (Silencer Select, Ambion) were used.

In our image analysis pipeline, 23 DEK body (DB) parameters were calculated and evaluated.

These were: 1) mean DB duration in DB sequences; 2) mean DB lifetime; 3) mean DB sequence length; 4) highest number of DB; 5) mean number of DB; 6) mean ratio of cells with 1 DB; 7) mean ratio of cells with 2 DB; 8) mean ratio of cells with 3 DB; 9) mean ratio of cells with 4 DB; 10) mean ratio of cells with 5 DB; 11) mean ratio of DEK sequences with > 1 DB; 12) mean ratio of DEK sequences with > 2 DB; 13) mean ratio of DEK sequences with > 3 DB; 14) mean ratio of DEK sequences with > 4 DB; 15) mean ratio of DEK sequences with > 5 DB; 16) mean ratio of DEK sequences with >0 DB; 17) mean ratio of DEK sequences with 1 DB; 18) mean ratio of DEK sequences with 2 DB, 19) mean ratio of DEK sequences with 3 DB; 20) mean ratio of DEK sequences with 4 DB; 21) mean ratio of DEK sequences with 5 DB; 22) median DB lifetime; 23) median DB sequence length.

The parameter which was impacted the most by the positive controls of the screen (siPOLD, APH, CPH) was mean number of DEK bodies and hence this parameter was chosen as final readout for this screen. We also expanded within the Methods section, which should now comprehensively describe the screen.

2.27 Supplementay Matetrial and Methods, Page 30, "[...] and the mean (XsNEG1)...". Should be $\bar{X}$ sNEG1. Also Xi should be defined.

Response: This has been changed accordingly. Xi is the mean number of DEK bodies for a given siRNA and calculated for every imaging position.

2.28 Page 11, "[...] corresponding to 44 and 28 different genes, respectively (Fig. 7B, C)" and Figure 7B. The authors should provide an exhaustive list of all up-regulators.

Response: We thank the reviewer for this comment. We added the z-score for every siRNA used in the high- and low-throughput screen to Supplementary Table 1 and 3, which now contains all relevant information.

2.29 Page 11, " we conducted a low-throughput secondary screen with 28 siRNAs (Fig. 7D)". Only 20 siRNAs are shown in the figure. Where do the authors show the effects of the remaining 8? Since it is a validation screen, one would expect to see them all.

Response: In Fig 7D (now Fig. 6D) we compared the results of the strongest hits from the primary and secondary screen, 10 siRNA each, showing that the positive hits from the SUMO pathway are found in both screens. All data from this screen can be found in Supplementary Table 3.

2.30 Page 11, " Still, the effect of up- and down-regulation of DEK body number was confirmed (Fig. 7D)". Not for SUMO3, based on Figure 7D, right graph. The authors should mention it and

© 2023. Published by The Company of Biologists under the terms of the Creative Commons Attribution License (https://creativecommons.org/licenses/by/4.0/).

comment, since they focus on SUMO3 later on. Actually, the statement "the secondary screen validated four top DEK body up-regulators from the primary screen belonging to the SUMO pathway, among them the E1 activating enzyme SAE1 and the E2 conjugating" is incorrect, if SUMO3 is part of them (which I believe it is, considering the last part of the results section). The authors should refine the analysis of their validation screen. Statistics?

**Response:** We have amended the respective section in the manuscript.

**2.31** Page 11, " Our screening approach thus determined that the SUMO pathway plays a major role in the regulation of DEK body formation.". In my opinion, this statement is too strong at this stage and should be tuned down.

**Response:** Based on the comments of this reviewer we have changed the wording in the relevant sections.

**DEK is a SUMO target and SUMOylation is required for the formation of DEK bodies**

**2.32** Page 11, " we focused on SUMO 1 and 3, as both were strong hits in the screens." As for my previous comments, SUMO 3 is not a strong hit based on the secondary (validation) screen.

**Response:** see response to the point above (2.31).

**2.33** Figure 8A. The authors should indicate on the figure or in the legend which antibody was used for IP. Should be against DEK, according to the main text and the material and methods. As it stands now in the legend, one cannot know what antibody was used for IP and/or detection. Also, I find it striking how the three immunoblots look alike, with a main band around 50 kDa in the IP lane and a faint one around 35 kDa for all of them. Is the faint band due to the secondary antibody used? Or a degradation product? Finally, why the authors do not detect any SUMO in the inputs?

**Response:** We thank this reviewer for pointing this out. We added more details to the Figure legend and now clearly explain which antibody was used for IP and for immunoblotting. We used the Veriblot detection system from Abcam, which only detects native antibodies. Thus, cross-reactivity with immunoglobulin chains of antibodies used for IP can be excluded.

For increased visibility of the input signals the blots from Figure 7A (former Fig. 8A) are shown below with increased contrast and reduced brightness. SUMO-3 is 11 kDa and a faint band at around 50 kDa can be detected in the SUMO-3 blot, this band would represent mono- SUMOylated-DEK. Unbound SUMO-3 will be detected by this antibody at 23 kDa and a faint band can be detected in the SUMO-2/3/4 blot below at around 23 kDa. These findings show the presence of unconjugated and conjugated SUMO-2/3/4 protein in the input. The employed anti- SUMO-1 antibody to a large extent detects SUMO-1 heterodimer with a molecular weight of 90 kDa. Faint bands can be detected in the input lane of the SUMO-1 blot in figure 8A as well as in the IP lane.

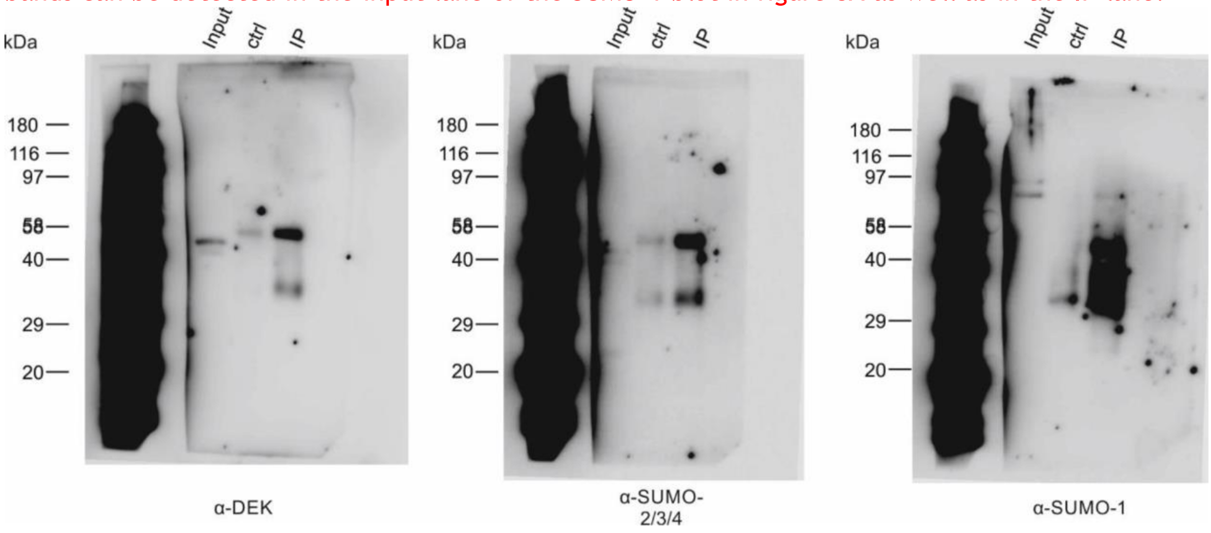


© 2023. Published by The Company of Biologists under the terms of the Creative Commons Attribution License (https://creativecommons.org/licenses/by/4.0/). 

**Fig. R 10**: Immunoblot analysis of co-immunoprecipitations performed using polyclonal rabbit anti-DEK antibody from U2-OS whole cell lysates. Shown are higher contrast images of the immunoblots from Figure 7A.

2.34 Page 11, "showing direct interaction of DEK with SUMO1 and SUMO2/3 (Fig. 8A)". If I read the figure correctly, the band that the authors detect using SUMO1 and SUMO2/3/4 antibodies correspond to DEK. Then it is not really a direct interaction, as we would expect from an interaction between the SIM (SUMO-interacting motif) of a protein and SUMO. The authors should rephrase this sentence. Also, according to the immunoblot performed after DEK IP and using antibodies against DEK for detection, there is only one band corresponding to DEK. Do the authors believe that DEK is always SUMOylated? Should we not expect several bands according to the level of SUMOylation otherwise? And when looking at the next panel (B), I am not sure to understand what we are looking at in these immunoblots (see my next comment).

**Response:** We thank the reviewer for this comment and agree that one would expect more than one band in the IP samples representing DEK modified with different levels of SUMO modification, unless DEK is only monoSUMOylated which we have not further investigated. As we see an interaction between DEK and SUMO after performing CO-IP we decided to try another approach: pulldown of SUMO and detection of DEK as interacting protein afterwards.

As SUMO-1 is only 12 kDa and SUMO-3 only 11 kDa the expected shift is not that big and indeed a slight shift can be detected in the IP signal on all shown blots independent of the antibody used.

2.35 Figure 8B. After spending a substantial amount of time on it, I do not understand the differences in terms of observed bands between these immunoblots and the ones presented in Figure 8A. Does the reader have to make their own interpretations of these results? What are we looking at in the middle and right immunoblots? Shouldn't we see several bands for SUMO in Figure 8A as well after DEK pull down? This series of experiments and their interpretations (corresponding to Figure 8A and 8B) should be better explained.

**Response:** See 2.34 above.

2.36 Legend of Figure 8C, "Densitometric quantification of mono-SUMOylated DEK signals from (B, left panel). DEK signals in pulldown samples were normalized to the corresponding input samples." How was this done? Which signals correspond to "mono-SUMOylated DEK". No information is provided anywhere as far as I could see. Also, there are some statistics provided. What are they? How was this done? How many experiments were performed?

**Response:** We thank this reviewer for pointing this out. We have added relevant information to the figure legend and also to the methods section.

2.37 Page 12, "we screened the DEK sequence for additional potential SUMOylation sites, which revealed amino acids 61, 62 and 144, 145 as suitable SUMO attachment sites." How did the authors identify these sites? This should be indicated.

**Response:** We used a prediction server http://jassa.fr/ (Joined Advanced Sumoylation Site and Sim Analyser) with the following settings: *Analyze with*: best predictions; *Results Sumo*: only “interesting” results; *Motifs*: all types w/o H&M; *Results SIM*: only “interesting” results; *Special environment*: none; *Output setting*: all lines. This returned a number of putative SUMO sites in DEK. We only focused on amino acids that were presented with the highest score (Best PS: Low) as outlined below.

© 2023. Published by The Company of Biologists under the terms of the Creative Commons Attribution License (https://creativecommons.org/licenses/by/4.0/). 

**Results for putatifs SUMO site**

| Position K | Sequence | Best PS | Consensus direct: Type | PSd | DB Hit | Consensus Inverted: Type | PSi | DB Hit |
|---|---|---|---|---|---|---|---|---|
| K48 | EDDEEEEEEEKEKSLIVEGKR | None | None | None | 1 | None | None | 1 |
| K50 | DEEEEEEEKEKSLIVEGKREK | None | None | None | 1 | None | None | 2 |
| K57 | EKEKSLIVEGKREKKKVERLT | None | Weak Consensus | None | | None | None | |
| K61 | SLIVEGKREKKKVERLTMQVS | Low | None | None | | None | Low | |
| K62 | LIVEGKREKKKVERLTMQVSS | Low | None | Low | | None | None | |
| K100 | EIERIHFFLSKKKTDELRNLH | None | None | None | 1 | None | None | 1 |
| K137 | VGQFSGFPFEKGSVQYKKKEE | None | None | None | | None | None | 1 |
| K144 | PFEKGSVQYKKKEEMLKKFRN | Low | None | Low | 5 | None | None | |
| K145 | FEKGSVQYKKKEEMLKKFRNA | Low | None | Low | 3 | None | None | 1 |
| K150 | VQYKKKEEMLKKFRNAMLKSI | None | None | None | 1 | None | None | 1 |
| K187 | KRILNFLMHPKPSGKPLPKSK | None | None | None | | None | None | 1 |
| K197 | KPSGKPLPKSKKTCSKGSKKE | None | None | None | | None | None | 2 |
| K205 | KSKKTCSKGSKKERNSSGMAR | None | None | None | 1 | None | None | 1 |
| K206 | SKKTCSKGSKKERNSSGMARK | None | None | None | 2 | None | None | 1 |
| K218 | RNSSGMARKAKRTKCPEILSD | None | None | None | | None | None | 1 |
| K237 | SDESSSDEDEKKNKEESSDDE | None | None | None | | None | None | 1 |
| K257 | EDKESEEEPPKKTAKREKPKQ | None | None | None | 1 | None | None | |
| K258 | DKESEEEPPKKTAKREKPKQK | None | None | None | | None | None | 1 |
| K261 | SEEEPPKKTAKREKPKQKATS | Low | Consensus | Low | 2 | None | None | |
| K264 | EPPKKTAKREKPKQKATSKSK | None | None | None | 1 | None | None | |
| K274 | KPKQKATSKSKKSVKSANVKK | None | None | None | | None | None | 2 |
| K278 | KATSKSKKSVKSANVKKADSS | None | None | None | 1 | None | None | 1 |
| K283 | SKKSVKSANVKKADSSTTKKN | None | None | None | 1 | None | None | 2 |
| K284 | KKSVKSANVKKADSSTTKKNQ | None | None | None | | None | None | 1 |
| K298 | STTKKNQNSSKKESESEDSSD | None | None | None | 1 | None | None | |
| K299 | TTKKNQNSSKKESESEDSSDD | None | None | None | 2 | None | None | 1 |
| K314 | EDSSDDEPLIKKLKKPPTDEE | None | None | None | | None | None | 2 |
| K315 | DSSDDEPLIKKLKKPPTDEEL | None | None | None | 1 | None | None | |
| K317 | SDDEPLIKKLKKPPTDEELKE | None | None | None | | None | None | 1 |
| K326 | LKKPPTDEELKETIKKLLASA | None | None | None | | None | None | 1 |
| K330 | PTDEELKETIKKLLASANLEE | None | None | None | | None | None | 1 |
| K367 | YDLTERKDFIKTTVKELIS-- | None | None | None | | None | None | 2 |
| K371 | ERKDFIKTTVKELIS------ | None | None | None | 1 | None | None | 3 |

**Results for putatifs SIM** [PSmax=38.183 | Cut-off=0.269]

| Position site | Sequence | Type | α/S stretch | PS | DB Hit |
|---|---|---|---|---|---|
| AA 163-166 | AMLKSICEVLDLERSGVNSE | SIM Type 1 | [N][SIM][Y] | 4.404 | 1 |

**Fig. R 11**. Output file of the SUMO prediction server http://jassa.fr/ with amino acids with higher SUMO probability indicated (accessed originally in 2018 and re-accessed on 2023-10-23). PS: putative site.

2.38 Figure 8D. The authors should show immunoblots with anti-SUMO-1 and anti-SUMO-2/3 as they did in Supplementary Figure 8.

**Response**: These have been added to panel D to Supplementary Figure 7.

2.39 Page 12, "[...] highlighting that most of the potential SUMOylation sites were captured by our mutational approach". Difficult to assess, since the authors do not provide information about how they did identify the other SUMOylation sites (see previous comment). There are maybe more sites, which are SUMOylated under specific conditions. This statement should be tuned down.

**Response**: We thank this reviewer for pointing this out. We revised this statement accordingly.

2.40 Page 12, " No DEK body formation was observed in cells expressing SUMO-less DEK (GST- DEK SUMO mut, Fig. 8F)." Actually, the whole DEK distribution pattern within the nucleus seems to be different between the WT and the mutant DEK. Indeed, even at this level of resolution, it seems that the mutant DEK is not really bound to anything anymore and accumulate in sub- compartment which I would instinctively identify as nucleolus. The fact that the authors do not observe DEK bodies with this mutant maybe due to something more general than just SUMOylation. The sites that the authors have identified as potential SUMO targets may be involved in other mechanisms that are independent of SUMO. Maybe these sites are not directly SUMOylated but rather involved in protein-protein (or protein-DNA?) interactions which are necessary for the SUMOylation of DEK? Is the mutant DEK even able to bind chromatin? Is it still "functional"? Based on the images in Figure 8F, I suspect a large fraction of the DEK mutant to be soluble. Several ways to verify this exist and should be easy to put in place, including a FRAP experiment in HeLa cells expressing wt or mutant DEK to look at the "chromatin" fraction, or a simple cell fractionation to look at the soluble fraction of DEK compared to the insoluble one. The authors should also provide pictures corresponding to DNA staining in Figure 8F, so one can rule out (or not) the accumulation of the DEK mutant in the nucleolus.

© 2023. Published by The Company of Biologists under the terms of the Creative Commons Attribution License (https://creativecommons.org/licenses/by/4.0/).

**Response**: We thank the reviewer for this comment, which was also raised by reviewer 1 (point 1.7). In addition to EMSA, where no reduced DNA binding of GST-DEK-SUMOmut was found, we performed new cell fractionation studies, which showed altered extractability of the GFP-DEK-SUMO mutant. In the immunoblot shown below a DEK-specific signal appears in the nucleosolic and cytosolic fraction, as it is released from chromatin at a lower NaCl concentration, indicative of altered chromatin binding as compared to WT. No non-modified GFP-DEK signal is visible in these fractions. We have included this new data in Supplemental Fig.8 and have commented on them in the Results and Discussion sections.

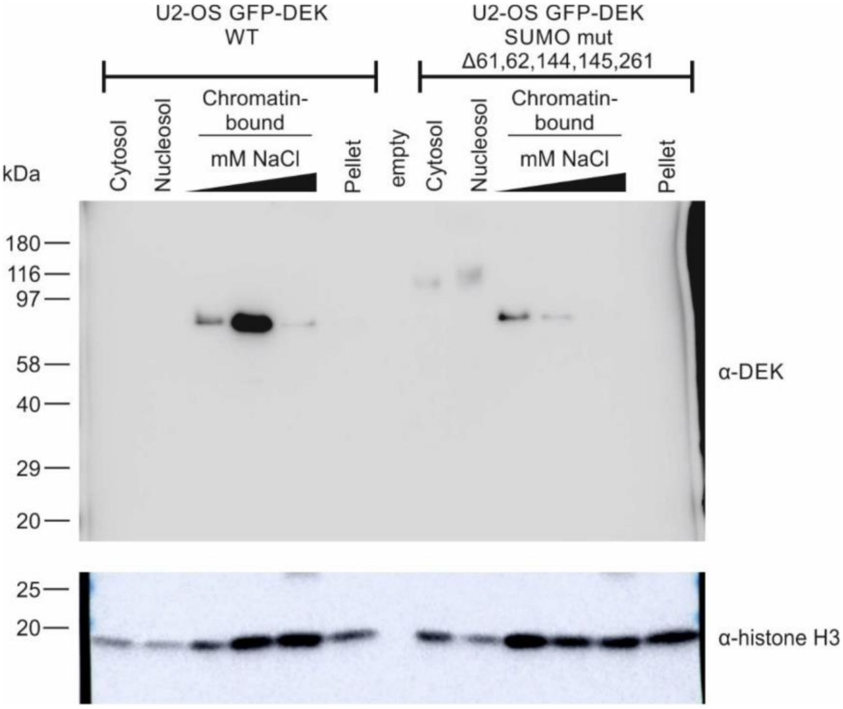


**Fig R 12**: Cell fractionation of U2-OS cells expressing GFP-DEK WT and DEK-SUMOmut. The cytosolic fraction was obtained by dounce homogenization of cell suspensions. The nucleosolic fraction was obtained by incubating cells with 0.5 % NP-40. The pellet containing nuclei and chromatin-bound proteins was treated with increasing concentrations of NaCl (100 mM, 250 mM, 450 mM). The remaining pellet was solubilized with RIPA buffer. Cell fractions were subjected to SDS-PAGE and immunoblotting. GFP-DEK was detected with DEK-specific polyclonal antibody (K-877 Ab) and as a loading control histone H3-specific antibody was used.

Finally, what about the formation of endogenous DEK bodies when the mutant is expressed? (in other words, is there a dominant negative effect?)

**Response**: This has been addressed in response to Reviewer 1 in point 1.7 b.

2.41 Page 12, " where SUMOylation of DEK seems to be required for DEK body formation". For the reasons I mentioned above, I think that more evidence should be provided to show that it is really the lack of DEK SUMOylation that is responsible for the absence of DEK bodies in the last experiment.

**Response**: We rephrased relevant sections in the manuscript.

**Discussion**

I found the discussion interesting to read and informative. However, I would like the authors to answer my comments regarding the role of SUMOylation of DEK in the process of DEK body formation before commenting on the last part of the discussion dealing with SUMO.

© 2023. Published by The Company of Biologists under the terms of the Creative Commons Attribution License (https://creativecommons.org/licenses/by/4.0/).

2.42 Page 15, " DEK body up-regulating siRNAs show a remarkable enrichment in proteins involved in post-translational modification pathways. Histone acetyltransferases (HATs) ACTL6A, KAT2B, TAF5L and TAF6L, histone deacetylase HDAC2, histone methyltransferases SUV39H1/2 and SUMOylation-related proteins UBE2I, SUMO1 and SAE1 are among the top hits." Yes, this is interesting. But why did the authors not mention it in the results section? As mentioned before, the presentation of results should be improved at several places.

**Response:** We thank this reviewer for pointing this out. We modified the results and discussion section accordingly.

2.43 Page 16, " SUMO-less DEK does not form bodies." I have already commented on that earlier, and I am not convinced that one can conclude this at the moment.

**Response:** see 2.42 above

2.44 Page 16, " This knowledge aligns well with our screening data, where downregulation of both SUV39H1 and SUMO increase the number of DEK bodies". Sorry, but I do not understand how. Also why is SUV39H1 not mentioned in the results part at all? Back to previous comments regarding the way some results are presented.

**Response:** see 2.42 above.

***************************************************************************************************************

**Reviewer 3 Advance Summary and Potential Significance to Field:**

The authors have investigated the subcellular localization of the DEK oncoprotein. Using immunofluorescence microscopy and cultured mammalian cells, they found that DEK localizes to distinct nuclear focal assemblies specifically in early to mid S-phase cells, which they term DEK bodies. Using co-localization studies, they demonstrated that DEK bodies exist in close proximity to active replisomes and are associated with markers of constitutive heterochromatin. Using a high-throughput screen, they dentified factors affecting DEK body formation, including multiple regulators of sumoylation. Using in vivo and in vitro assays, they demonstrated that DEK is sumoylated at multiple lysine residues. A mutant of DEK with reduced sumoylation failed to form DEK bodies when expressed in cells. The authors conclude that DEK is important for heterochromatin integrity and that its function is regulated through sumoylation. DEK is an oncoprotein with functions associated with DNA replication and replication stress. Its precise functions are not fully characterized, and associations with discrete chromatin domains and nuclear foci has not been previously described. The findings reported in this study are therefore novel and provide new insights into DEK function that may be relevant to better understanding its role in oncogenesis. Overall, the manuscript is well written and the data are mostly of high quality.

**Response:** We would like to thank this reviewer for the positive comments on our work and the very thoughtful and helpful suggestions which we addressed below.

**Reviewer 3 Comments for the Author:**

There are two major issues that need to be addressed:

3.1 Much of the fluorescence microscopy data is presented as a single cell. Quantitative, population-based data would strengthen the conclusions.

**Response:** We thank this reviewer for pointing out this shortcoming, which was also raised by reviewers 1 and 2. As outlined in 1.4 we have now increased the cell numbers and replicates for many experiments and indicated statistical analyses at the respective positions. See also points 1.5, 2.3, 2.4, 2.11, 2.13, 2.23, 2.24 and 2.26 in responses to Reviewers 1 and 2.

3.2 Specific questions that should be addressed include:

Figure 1 - (1) what fraction of S-phase cells show DEK bodies?,

© 2023. Published by The Company of Biologists under the terms of the Creative Commons Attribution License (https://creativecommons.org/licenses/by/4.0/). 

**Response**: From our high-throughput screening data with high cell numbers, we conclude that every cell traversing through S-phase shows DEK bodies. However, as the screen was performed by high-throughput, low resolution microscopy, faint DEK bodies could occasionally escape detection. In contrast, our high-resolution imaging never failed to detect DEK bodies in late S- phase in MCF-10 and U2-OS cell lines.

3.3 what fraction of Edu and PCNA positive foci are associated with DEK bodies?

**Response**: We thank this reviewer for this comment, which was also raised by reviewer 2. Please see our response under point 2.11.

3.4 Figure 2 - (1) is the data supported by analysis of multiple DEK bodies analyzed across multiple cells?

**Response**: We analyzed a total of 24 MCF10A cells using SMLM and STED, however super-resolution techniques were not applied in other cell types. Please see Table in 1.4 for more details. Below, we show more examples of DEK bodies immunolabelled in MCF10A cells and imaged using STED.

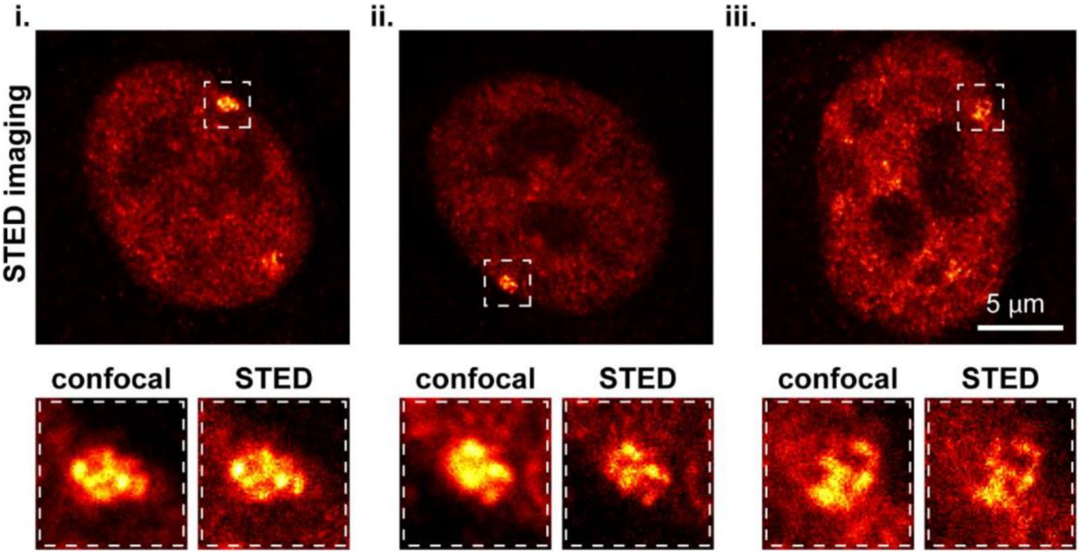


**Figure R 13:** Additional examples of super-resolution imaging of DEK bodies in MCF10A cells. The insets show the comparison between confocal and STED imaged DEK structures visualized thanks to immunostaining against DEK.

3.5 Figure 3 - (1) with each marker (H3K9ac, H3K27me3, and H3K9me3) what fraction of DEK foci show no overlap, spatial juxtaposition or colocalization? This question is relevant given that in the image in 3A showing DEK:H3K9me3 there are DEK foci showing colocalization (the one analyzed) and juxtaposition.

**Response**: The presented Figure 3B with the Manders' coefficient partially answers for this subject. To analyze the lack of overlap, spatial juxtaposition or colocalization more quantitatively, we performed a thorough analysis of each DEK body region signal overlapping with the histone modification signal. We calculated Manders' coefficient for each DEK body signal (101 cases), defining as three cases: (A) "not overlapping signals" as $M1 < 0.25$, (B) "juxtaposing signal" as $0.25 < M1 < 0.75$, and (C) "colocalizing signal" as $M1 > 0.75$. In sample with DEK and H3K9ac immunostained these fractions were: (A) 62%, (B) 21%, and (C) 17%. For DEK and H3K27me3 it was: (A) 46%, (B) 49% and (C) 5%. For DEK and H3K9me3 we obtained: (A) 14%, (B) 29%, and (C) 57%.

3.6 (2) The Manders' coefficients analysis should be explained.

**Response**: We thank the reviewer for pointing this out. We explained Manders's analysis in point 2.13 in response to reviewer 2 and also added more details to the methods section of the revised manuscript.

© 2023. Published by The Company of Biologists under the terms of the Creative Commons Attribution License (https://creativecommons.org/licenses/by/4.0/).

3.7 Figure 4 - (1) what fraction of DEK bodies associate with centromeres, lamin invaginations and Xist, and vice versa?

**Response**: We thank this reviewer for pointing this out, which was also raised by reviewer 2. Please find detailed answers to this question in points 2.16 to 2.21 in the response to reviewer 2. The former Fig. 4 is now presented as Supplementary Fig. S5.

3.8 There are a number of issues with the sumoylation data in Figure 8. (1) In Figure 8A, here are no, or barely detectable, SUMO signals in the input lanes for the anti-SUMO1 and SUMO2/3/4 blots, raising a concern about the effectiveness of these antibodies. In addition, it is odd that the bands in the IP lanes for the anti-DEK, SUMO1 and SUMO2/3/4 blots are all of similar molecular weights (sumoylation at one site should cause a ~15 kDa shift in DEK, but this does not appear to be the case). There is a concern that the signals in the IP lane correspond to antibody heavy and light chains. Were similar amounts of control and DEK antibodies used - Ponceau S stained blots showing antibody chains in control and DEK antibody IPs would help. (s2)

In Figure 2B, there are no or barely detectable high molecular weight signals corresponding to SUMO2 or SUMO3 conjugated proteins seen in the anti-SUMO2/3/4 blot in either the input or the pulldowns. This raises suspicions about the high molecular weight DEK bands detected in the anti-DEK blot. The presence of such high molecular weight bands is also inconsistent with results presented in 8A.

**Response**: The high molecular weight bands are hard to detect but there are signals in the pulldown lanes. For better detection we increased brightness and contrast of the blot below.

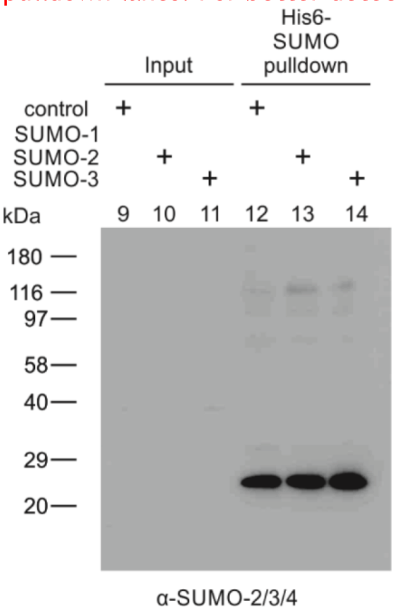


**Fig. R14**: Immunoblot analysis of His-SUMO pulldown experiments from HeLa cells expressing 6xHis- SUMO-1, 6xHis-SUMO-2, 6xHis-SUMO-3, or control cells after nickel affinity chromatography. Shown are higher contrast images of the immunoblots from Figure 7B.

In contrast to SUMO-1, SUMO-2/3 is found mostly unconjugated under physiological conditions (*Saitoh, H., & Hinchey, J. (2000). Functional heterogeneity of small ubiquitin-related protein modifiers SUMO-1 versus SUMO-2/3. J Biol Chem, 275(9), 6252-6258. doi:10.1074/jbc.275.9.6252*), explaining the thick band at 23 kDa. As only a small fraction of cellular proteins are conjugated to SUMO-2/3 under our experimental conditions, detection with DEK-specific antibody shows that DEK has to be one of them. The missing input signal could be based on the low amount that was loaded as input (1 % of total lysate) and maybe the signal is below the threshold level that can be detected by the used SUMO-2/3/4-antibody. See also point 2.33 in response to reviewer 2.

Results in Figure 8F should include quantification across multiple cells. It would also be helpful to do co-localization with Edu or PCNA to verify that there are S phase cells present in the mutant

© 2023. Published by The Company of Biologists under the terms of the Creative Commons Attribution License (https://creativecommons.org/licenses/by/4.0/).

expressing cells. It also appears that the mutant protein is concentrated in nucleoli, unlike the wild type protein. This should also be noted and quantified.

**Response**: Please see response to reviewer 1 in point 1.7b.

**3.9** Lastly, it is a stretch to conclude that the observed defect in DEK body formation is due to a defect in DEK sumoylation. Lysine to arginine substitutions are also expected to disrupt possible ubiquitylation, acelylation and methylation at these sites. Can the defect be rescued with a DEKmutant-SUMO fusion protein? Collectively, these issues raise serious concerns about the role that DEK sumoylation may play in affecting DEK body formation. Moreover, the findings that more DEK bodies are formed when sumoylation is inhibited is inconsistent with the DEK SUMO mutant result and suggests that other substrates may be more relevant.

**Response**: We agree with the reviewer that a rescue experiment would be the most unequivocal demonstration that SUMOylation of DEK is directly required for DEK body formation. As we presently can´t provide these data, we have performed additional experiments that in our view support the conclusion that 1. The SUMO pathway is involved in the regulation of DEK body formation and 2. SUMOylation acts in concert with other posttranslational modifications of DEK to determine its chromatin association behavior and thus DEK body formation. We have rephrased several passages of the manuscript to account for this shift in perspective as detailed in this point-by-point rebuttal.

---

Second decision letter

MS ID#: JOCES/2023/261329

MS TITLE: DEK oncoprotein participates in heterochromatin replication via SUMO-dependent nuclear bodies

AUTHORS: Agnieszka Pierzynska-Mach, Christina Czada, Christopher Vogel, Eva Gwosch, Xenia Osswald, Denis Bartoschek, Alberto Diaspro, Ferdinand Kappes, and Elisa Ferrando-May
ARTICLE TYPE: Research Article

I am happy to tell you that your manuscript has been accepted for publication in Journal of Cell Science, pending standard ethics checks.

Reviewer 1

*Advance summary and potential significance to field*

This manuscript is the first to report, and characterize, the subcellular distribution of the chromatin remodeling DEK protein into “DEK bodies” within the nucleus. DEK is an under-studied protein with little known about its post-
translational regulation and functions. Aberrant DEK expression or localization has been linked to cancers, autoimmune diseases, and neurodegernative diseases.
Therefore, understanding its biochemical functions and regulation have broad impacts for understanding the molecular pathogenesis of disease.

*Comments for the author*

The authors have adequately addressed this reviewer’s concerns.

Reviewer 2

*Advance summary and potential significance to field*

© 2023. Published by The Company of Biologists under the terms of the Creative Commons Attribution License (https://creativecommons.org/licenses/by/4.0/).

As mentioned in my previous review, the authors identified an accumulation of the oncoprotein DEK at late DNA replication sites, which correspond to constitutive heterochromatin. How DNA embedded in the complex structure of heterochromatin is replicated is a very active field of research, as it is also associated to the epigenetic memory of these regions. Finding a new factor potentially involved in the replication of heterochromatin is, therefore, of interest for the field, especially since its accumulation at late replicating sites seems to be modulated by SUMO, a protein already found to accumulate at these sites in other systems. The findings from this study will definitely participate to a better understanding of the mechanisms underlying the replication of heterochromatin and give new directions for investigating this process. In my opinion it is now, after revisions, suitable for publication in Journal of Cell Science and should be of great interest for its readers, especially for those interested in chromatin dynamics.

*Comments for the author*

I thank the authors for their tremendous work to make their manuscript clearer and much more convincing. They did tune down some of their interpretations/conclusions, which make the whole manuscript more accurate and let the door open for more investigations. All my comments have been thoroughly and satisfactorily addressed, and the authors did, indeed, satisfied my curiosity. I have no further comments and find the present manuscript suitable for publication in Journal of Cell Science as it is.
All the best.
Erwan Delbarre

Reviewer 3

*Advance summary and potential significance to field*

The findings provide new insights into functions of DEK and its role in oncogenesis. The findings also provide evidence of a role for sumoylation in regulating formation of nuclear bodies that may have broad significance.

*Comments for the author*

The authors have made considerable revisions to the manuscript based on the comments of three previous reviewers. Overall, the work is significantly improved. Concerns over results in revised Figures 7A and B, however, still remain. It remains unclear how anti-DEK and anti-SUMO antibodies detect a protein of the same molecular weight in the DEK IP fraction. The anti-DEK western should detect both unmodified DEK as well as modified DEK in the IP fraction, but only a single band is observed. The authors appear to be arguing that this represents mono-sumoylated DEK. This would have to mean that DEK is 100% sumoylated, but this is not supported by the lower molecular weight band detected in the input lane. There really seems to be something off with these 7A western blots. In these blots and the blots in 7B, there is still a concern that no, or very minimal SUMO signals, are detect in the input lanes (or the pulldown lanes from his-SUMO2 and his-SUMO3 expressing cell lysates). The argument that SUMO2/3 are largely not conjugated to proteins in HeLa cells is really not correct (high molecular weight conjugates can be detected in whole HeLa cell lysates in many published studies). Altogether these western blots do not make a satisfactory case for DEK sumoylation. The results of the siRNA screen make a good case for a role of SUMO in DEK body formation. The in vitro DEK modification data and mutant protein analysis data make a suggestive case for DEK being a direct and functionally relevant substrate. The authors can make their case based on these results and also make the suggestion that sumoylation of other factors in addition to DEK may be involved in formation of DEK bodies (which would be in line with the finding that multiple proteins are sumoylated in PML nuclear bodies).

© 2023. Published by The Company of Biologists under the terms of the Creative Commons Attribution License (https://creativecommons.org/licenses/by/4.0/).
